# Exploratory and discriminant analysis of plant phenolic profiles obtained by UV–vis scanning spectroscopy

**DOI:** 10.1515/jib-2019-0056

**Published:** 2021-06-04

**Authors:** Monique Souza, Jucinei José Comin, Rodolfo Moresco, Marcelo Maraschin, Claudinei Kurtz, Paulo Emílio Lovato, Cledimar Rogério Lourenzi, Fernanda Kokowicz Pilatti, Arcângelo Loss, Shirley Kuhnen

**Affiliations:** Universidade Federal de Santa Catarina, Florianopolis, Brazil; EPAGRI, Ituporanga, Santa Catarina, Brazil; Instituto Federal Sul-rio-grandense, Pelotas, RS, Brazil; Escola do Mar, Ciência e Tecnologia da Universidade do Vale do Itajaí, (UNIVALI), Itajaí, Brazil

**Keywords:** chemometrics, cover crops, multivariate analysis, R language, specmine

## Abstract

Some species of cover crops produce phenolic compounds with allelopathic potential. The use of math, statistical and computational tools to analyze data obtained with spectrophotometry can assist in the chemical profile discrimination to choose which species and cultivation are the best for weed management purposes. The aim of this study was to perform exploratory and discriminant analysis using R package specmine on the phenolic profile of *Secale cereale* L., *Avena strigosa* L. and *Raphanus sativus* L. shoots obtained by UV–vis scanning spectrophotometry. Plants were collected at 60, 80 and 100 days after sowing and at 15 and 30 days after rolling in experiment in Brazil. Exploratory and discriminant analysis, namely principal component analysis, hierarchical clustering analysis, *t*-test, fold-change, analysis of variance and supervised machine learning analysis were performed. Results showed a stronger tendency to cluster phenolic profiles according to plant species rather than crop management system, period of sampling or plant phenologic stage. PCA analysis showed a strong distinction of *S. cereale* L. and *A. strigosa* L. 30 days after rolling. Due to the fast analysis and friendly use, the R package specmine can be recommended as a supporting tool to exploratory and discriminatory analysis of multivariate data.

## 1 Introduction

Scanning UV–vis spectrophotometry has many advantages on the analysis of plant extracts. Among them, it can be highlighted the small amount of sample required, easy preparation of the samples and fast data acquisition, especially that related to specific classes of secondary metabolites [1, 2], such as phenolic compounds [3–5].

Some species of angiosperms used as cover crops, like black oat (*Avena strigosa* L.) and rye (*Secale cereale* L.), both from Poaceae family, and oilseed radish (*Raphanus sativus* L.) from Brassicaceae family, are known for producing phenolic compounds, particularly related to weeds control [4–8].

Most of the studies addressing these compounds aim on their quantification [9], [10], but few explore chemical data from UV–vis profiles to discriminate species according to their phenolic composition.

Spectroscopic methods that use wavelengths, like UV–vis and infrared, are faster and require less or no chemical processing, compared to chromatographic methods. With the support of mathematical and statistical tools, it is possible to process spectra and identify patterns of metabolic fingerprints, enabling to discriminate samples according to their similar or disparate characteristics [11].

Spectra obtained by UV–vis scanning spectrophotometry usually have many peaks the occasional similarity of sample profiles make essential the use of bioinformatic tools, such as discriminatory analysis, in order to obtain important and additional information [2], [12], [13]. Using the datasets of spectrophotometric profiles, it is possible to build descriptive and classification models that enable to explore those profiles. The application of mathematical and statistical methods, such as univariate and multivariate analysis, in association with complementary techniques for the detection of compounds, for example, spectrometry (UV–vis, NIR, NMR), are useful tools to assist in the characterization and discrimination of samples in the Chemistry field [14–18].

In this context, with the application of chemometric methods it is possible to investigate, interpret, classify and separate spectra profiles of complex matrices, pre-processed or not, within any range of the spectrum, acquired with UV–vis spectrophotometry, infrared spectroscopy or nuclear magnetic resonance [15], [19], [20]. Due to the volume of information and the complexity of data, the most popular approaches to identify global differences between the samples include non-supervised methods, like principal component analysis (PCA) and hierarchical clustering analysis (HCA) [16], [21].

Aiming to simplify the analysis of spectral profiles and the prospecting of big datasets, the use of free computational environments, like the R language and its biostatistics packages and tools, can help to comprehend the relations between the variables under analysis. Our hypothesis is that the use of these tools will enable to identify spectral regions that discriminate the phenolic profiles according to features such as plant species, phenological stage, and farming conditions.

In this sense, the aim of this work was to perform exploratory and discriminatory analysis on phenolic profiles of *S. cereale* L. *A. strigosa* L. and *R. sativus* L. shoots obtained by UV–vis scanning spectrophotometry.

## 2 Materials and methods

### 2.1 Samples

The experiment was conducted at the Experimental Station of the Company of Agriculture Research and Rural Extension of Santa Catarina (EPAGRI) in the city of Ituporanga, Santa Catarina State (27° 24′ 52″, 49° 36′ 9″ and altitude 475 m). The climate of the region is humid subtropical (Cfa), according to the Köppen classification, with a mean annual temperature of 17.6 °C and a mean annual rainfall of 1.400 mm. The soil of the area was classified as Humic Dystrudept [22].

The experiment was installed in an area with a 20-year history of onion cultivation under conventional tillage (CT) (plowing, harrowing, and scarification) until 1996. From 1996 to 2007, a minimum cultivation system was implemented for onion in rotation with the following cover crop species: black oat (*A. strigosa* Schreb), velvet bean (*Mucuna aterima* Piper & Tracy), millet (*Pennisetum glaucum* L.), sunn hemp (*Crotalaria juncea* L.), and vetch (*Vicia sativa* L.). The area was then grown with sweet potato (*Ipomoea batatas* (L.) Lam.) until 2009, when the non-tillage (NT) experiment with onion was installed. The weeds were desiccated with glyphosate at the beginning of the experiment (April 2009).

Five treatments were used for winter cover crop, considering plant species and crop management system (single or intercropped): black oat (120 kg of seeds ha^−1^); rye (120 kg of seeds ha^−1^); oilseed radish (20 kg of seeds ha^−1^) (NF); oilseed radish (10 kg of seeds ha^−1^) + rye (60 kg of seeds ha^−1^); oilseed radish (10 kg of seeds ha^−1^) + black oat (60 kg of seeds ha^−1^). The seeds of these winter species were sown every year in April. The seeds were sown by hand on the soil surface. There was no fertilization, irrigation, or crop management during the cover crop cycles. In 2014, single and intercropped rye shoots were collected at five periods: 60, 80 and 100 days after sowing (DAS) and 15 and 30 days after rolling (DAR). Oilseed radish single and intercropped (rye and black oat) were collected at four periods: 60, 80 and 100 days after sowing (DAS) and 15 days after rolling (DAR). Black oat, single and intercropped, was collected only at three periods: 100 DAS and 15 and 30 DAR. Three subsamples were randomly collected from each plot to make one composed sample. Each species was separately collected and later evaluated, including those from the intercrops. The experimental design was randomized blocks with three replicates. The area of each experimental plot was 5 × 5 m^2^.

A total of 84 samples were collected from the field experiment, identified, put in falcon tubes (50 mL) and kept in a Styrofoam thermal box with dry ice during the transport to the laboratory. Samples were lyophilized at −54 °C (model L101, Liotop, São Paulo, Brazil) until total removal of moisture, pulverized, sieved (0.42 mm) and kept at −20 °C until analysis.

### 2.2 Extraction and analysis through scanning UV–vis spectrophotometry

For the extracts, the plant material was macerated with methanol (Vetec) 80% (v/v) (1:50, w/v), shaken for 2 h and filtered under vacuum. The extracts were then centrifuged at 4000 rpm for 15 min. The supernatants were collected and subjected to UV–vis scanning spectrophotometry (model UV-5300PC, Power Supply, China), for the acquisition of spectral profiles (200–800 nm). Each sample was scanned three times, resulting in a dataset with 252 spectral profiles.

### 2.3 Chemometric analysis

All the analyses were performed using R language (R^©^ v. 3.3.1) [23] with R Studio, and tools and functions available in the package ‘specmine’ [24]. All the scripts were written with the package R Markdown and the reports were automatically generated. Two data sheets were created, one named “metadata”, holding information about treatments (plant species and crop management system) and periods of sampling, and other named “data”, containing data from the spectral profiles in the range of 200–800 nm, both saved in the “xlsx” format. To import those data sheets into the R environment, both files were converted to “csv” format.

‘specmine’ is an R package with functions that enable to perform univariate analysis to big data set with hundreds of variables, in example, the wavelengths from our data. Thus, data obtained from the UV–vis spectral profiles was subjected to univariate statistics *t* test, fold-change test and analysis of variance (ANOVA), and to the multivariate unsupervised methods principal components analysis (PCA) and hierarchical cluster analysis from correlated matrices. Supervised machine learning based on partial least squares regression (PLS) and k-nearest neighbor (knn) models were applied to the dataset to determine the best predictive model for sample classification. The error estimation method was repeated by 10-fold cross-validation and 10 repetitions.

## 3 Results and discussion

**Figure 1:**
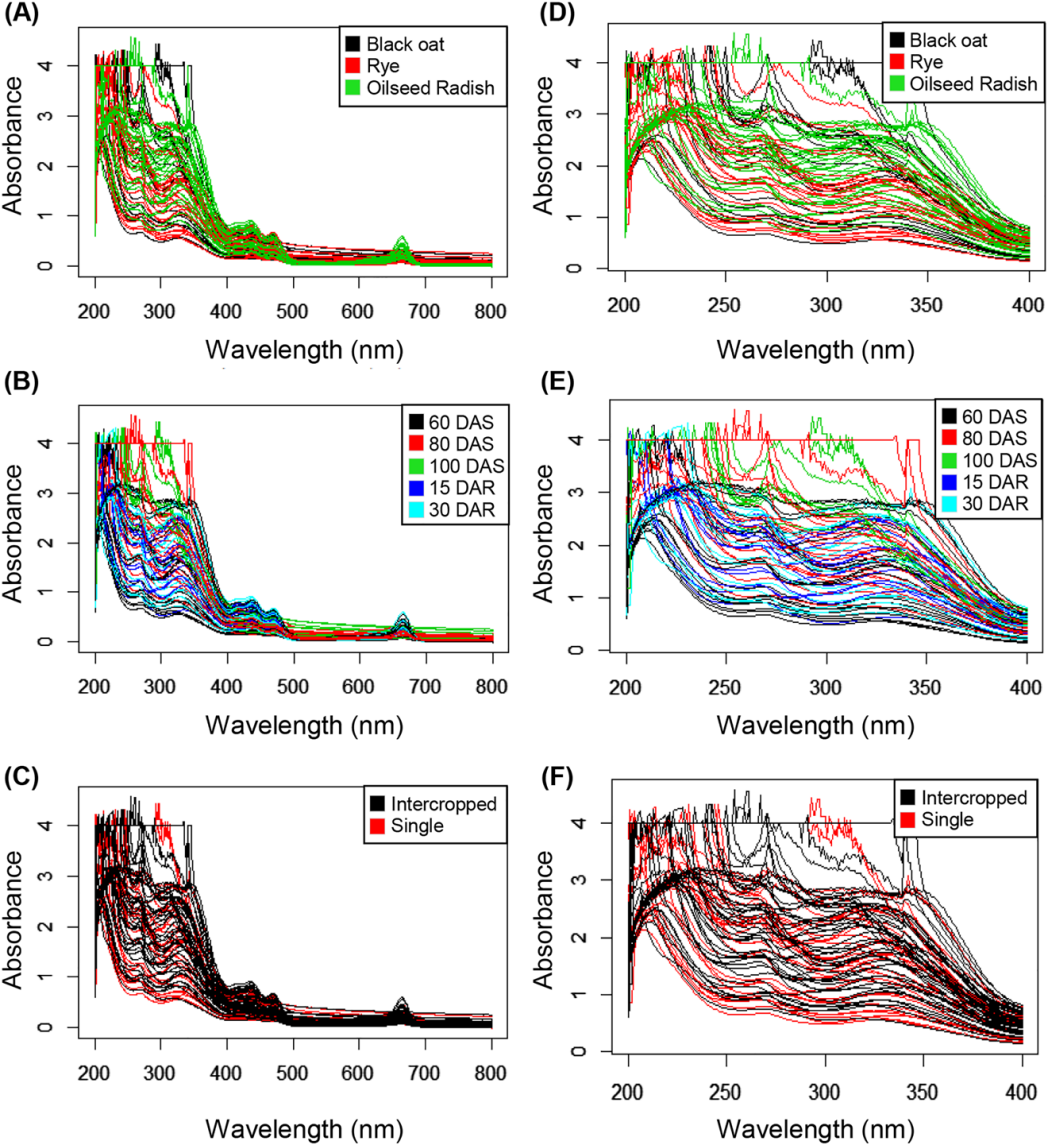
UV–vis spectra profiles of the methanolic extract of plant shoots in the region of 200–800 nm before pre-processing (A–C) and 200–400 nm (D–F). The colors of the spectra indicate the plant species black oat (*Avena strigosa* L.), rye (*Secale cereale* L.) and oilseed radish (*Raphanus sativus* L.) (A and D), the periods of sampling (days after sowing-DAS and days after rolling-DAR) (B and E) and crop management system (single or intercropped) (C and F).

With the tools from the package specmine it is possible to explore the whole spectral region obtained via UV–vis spectroscopy in the dataset or to crop specific regions, depending on the compounds to be analyzed and the aim of the research. In the present work, the spectral profiles in the region 200–800 nm from the shoots of rye, black oat and oilseed radish, grown single or intercropped, at 60, 80 and 100 DAS and 15 and 30 DAR were evaluated (Figure 1A–C) and the region of 200–400 nm (Figure 1D–F) was cropped to be used in some analysis.

**Figure 2:**
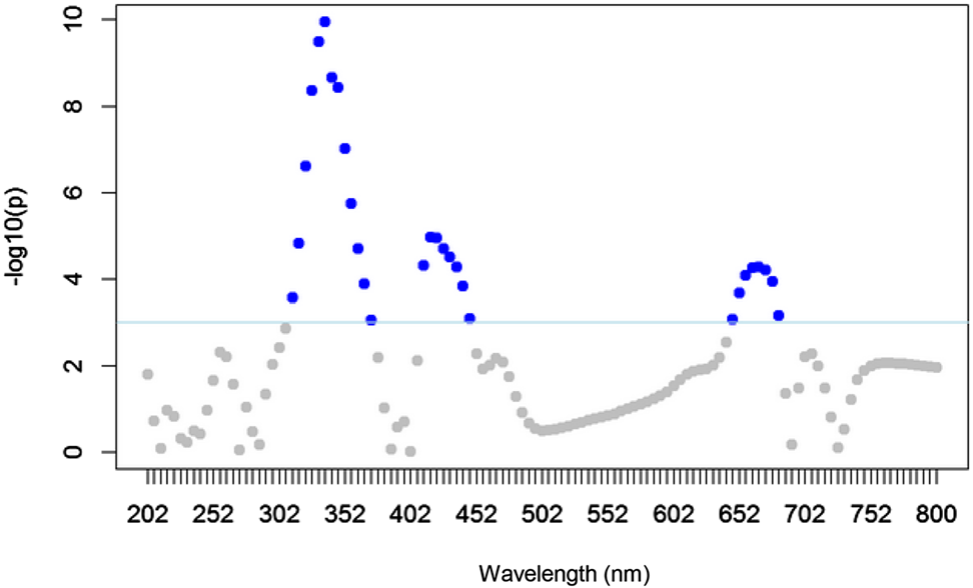
Fold-change analysis of the UV–vis spectra dataset (200–800 nm). Blue dots indicate wavelengths with significant differences (*p* < 0.05). Samples were statistically different in the region between 300 and 450 nm (phenolic compounds and carotenoids) and 650–700 nm (chlorophylls).

Initially the analysis of the 84 spectra showed similar profiles between the samples and the highest values of absorbance in the region of 200–400 nm (Figure 1A–C), which is the region attributed to the phenolic compounds. Most of the phenolic compounds and flavonoids have their absorption range around 280 nm [25], [26]. This was supported by the fold-change analysis (Figure 2).

The fold-change analysis is usually used in analysis of profiles and when multiple measurements are being analyzed [2], [11], [27]. In this study, the fold-change analysis indicated difference between rye, oilseed radish and black oat in the region of 300–450 nm, the typical region of absorbance of phenolic compounds and carotenoids. In addition, some signals in the region of 650–700 nm, typical region of absorbance of the chlorophylls, had significant signal intensity (Figure 2).

This approach corroborates the univariate analysis performed on the spectra, expressed via statistical significance of the model by the descriptive *p*-value. In fact, the wavelengths with the lowest *p*-values were observed in the spectral window of 300–420 nm (data available in the supplementary material). Samples showed intense absorbance in the wavelength regions assigned to the phenolic compounds (Figure 1A), corroborating literature that reports that these compounds as commonly present in black oat, rye and oilseed radish [7], [8], [28], [29]. Thus, although small discrepancies between the profiles exist, they are difficult to be identified. At first sight, the spectra overlaps might suggest similarity in the chemical composition of the species, so it is necessary to use tools that help to interpret data.

**Figure 3:**
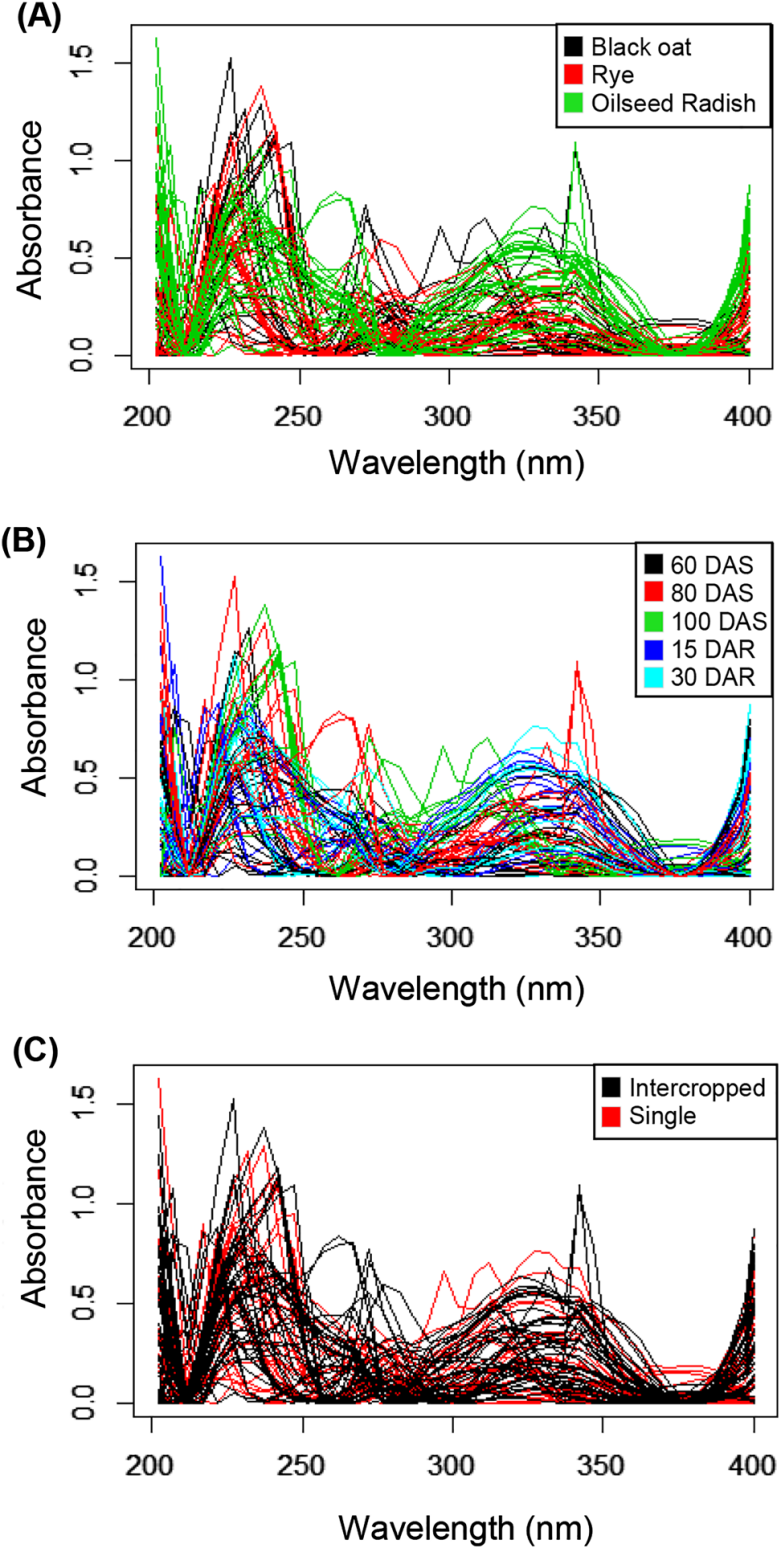
UV–vis spectra profiles of the methanolic extract of plant shoots in the region of 200–400 nm after pre-processing. The colors of the spectra indicate the plant species black oat (*Avena strigosa* L.), rye (*Secale cereale* L.) and oilseed radish (*Raphanus sativus* L.) (A), the periods of sampling (days after sowing-DAS and days after rolling-DAR) (B) and crop management system (single or intercropped) (C).

Language R has packages and functions with mathematical tools to transform, pre-process signals and spectra, like specmine. In this study the pre-processing was used to baseline correction, smoothing and cropping (200–400 nm) to the spectra (Figure 3A–C). Although it was possible to identify statistical differences and discriminate samples when the dataset was analyzed globally or cropped, the clusters obtained after pre-processing can reinforce the similarity between the samples previously observed or bring new information for subsequent analysis [11].

**Figure 4:**
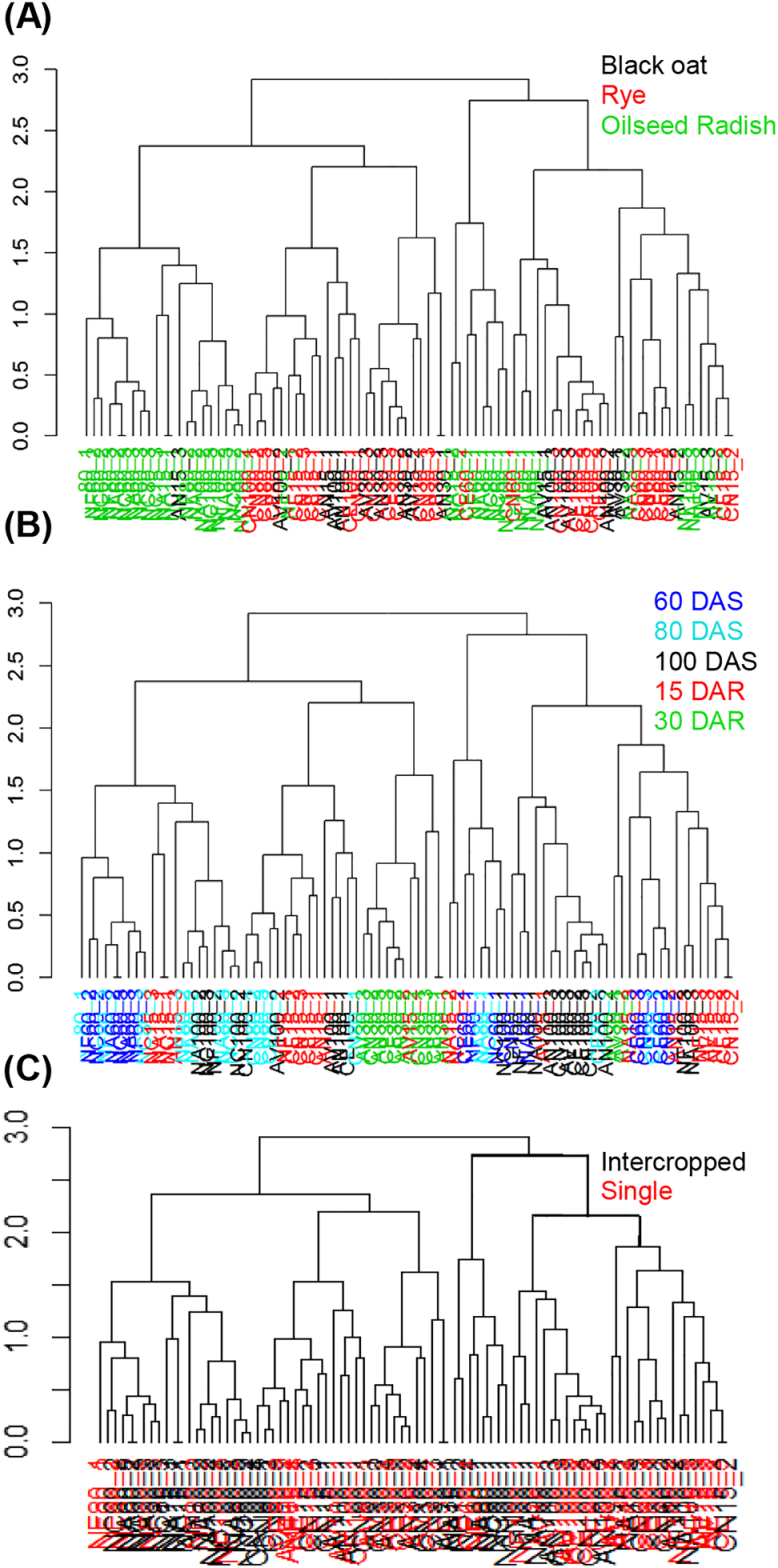
Dendrograms of UV–vis spectra in the region of 200–400 nm after hierarchical clustering analysis using the Euclidean distance. Colors indicate plant species (A), period of sampling (days after sowing-DAS and days after rolling-DAR) (B) and crop management system (C).

Based on the information obtained observing the plots, different statistical analysis, like multivariate analysis and hierarchical clustering, were performed on the entire spectral region and in the region between 200 and 400 nm, aiming to detect differences between the profiles in the spectral region related to the phenolic compounds [30]. The spectral profile of oilseed radish was different from those of rye and black oat, which were similar to each other (Figure 3A). Regarding to the time of cropping when the samples were collected, only the samples collected at 30 DAR segregated in the hierarchical clustering (Figure 4B).

**Figure 5:**
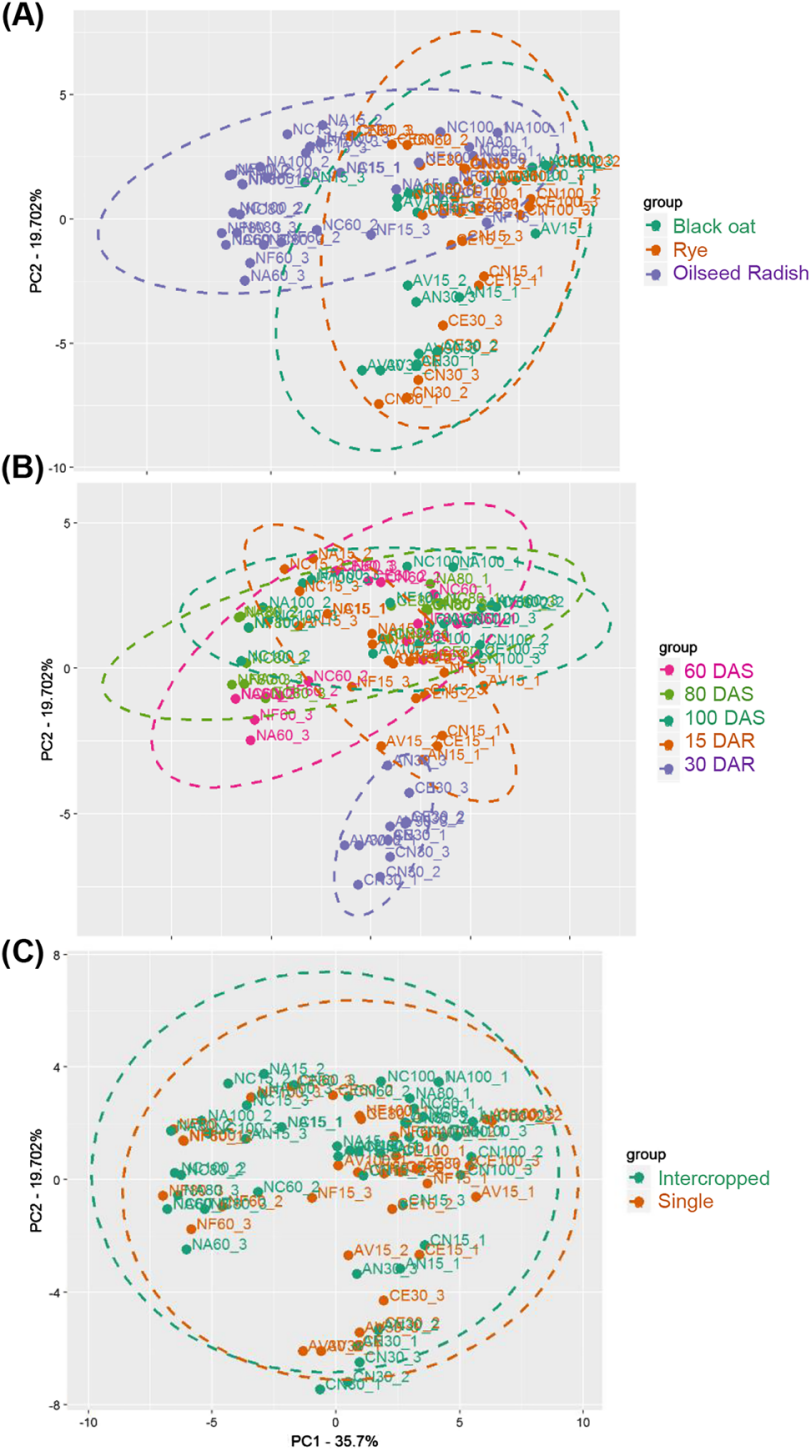
Factorial distribution of principal components 1 (PC1) and 2 (PC2) of the UV–vis spectral profiles of plant methanolic extracts in the region of absorbance of phenolic compounds (200–400 nm) after pre-processing. Colors indicate plant species (A), periods of sampling (days after sowing-DAS and days after rolling-DAR) (B) and crop management system (single or intercropped) (C).

Next, with the principal component analysis, it was possible to see how the samples clustered according to their differences and similarities based on UV–vis spectral data, with dimensionality reduction of the dataset but preserving the information and calculating linear combinations between the original variables [31]. Thus, the present study performed PCA on the UV–vis spectral profiles after pre-processing the region between 200 and 400 nm to assess clusters based on differences or similarities in the phenolic composition of the samples. Figure 5 shows the score plot from the PCA performed on the same dataset used in the hierarchical clustering analysis (Figure 4) for rye, black oat and oilseed radish samples.

**Figure 6:**
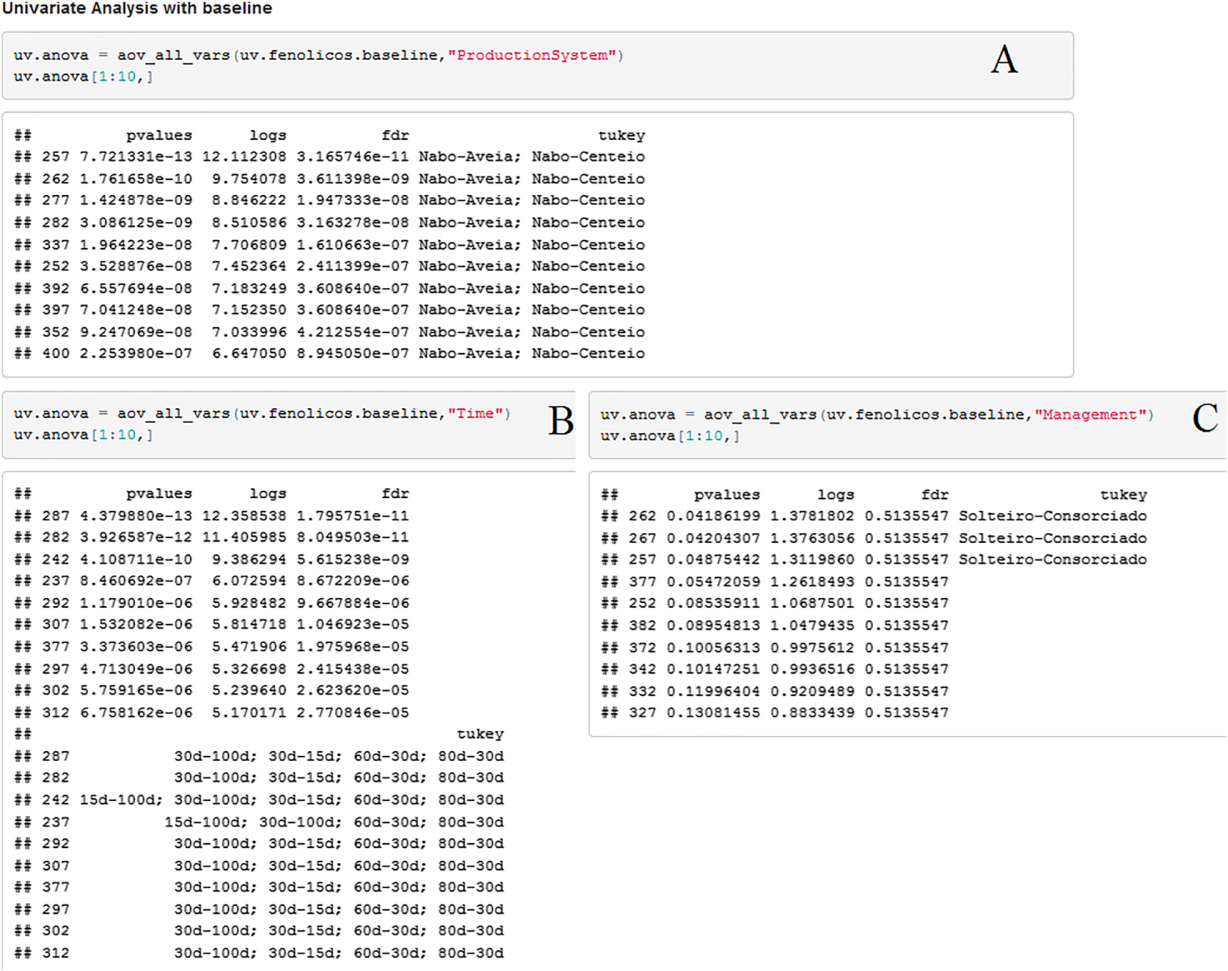
Univariate analysis performed on the UV–vis spectra and the wavelengths that contributed the most for the clustering according to plant species (A), period of sampling (B) and crop management system (C).

In the PCA of the spectral profiles, PC1 and PC2 explained 55.4% of the variance in the data, segregating single and intercropped oilseed radish from the Poaceae black oat and rye. These results point to a distinction between these species concerning to their phenolic composition as well as other compounds that absorb in the UV range (Figure 5A). Rye samples, for all the evaluation times, are situated at the score plot PC1(−), while rye and black oat samples at 60, 80 and 100 DAS are at PC1(+). Rye and black oat samples after rolling are at PC1(+) and PC2(−) (Figure 5A). The wavelengths that had greater contribution for the clustering were 252, 257, 282, 352, 392 and 397 nm (*p* < 0.05) (Figure 6A). The oilseed radish, a member of the Brassicaceae family, is known for its allelopathic activity and phenolic compounds production [29], [32], which are mainly concentrated in the shoots [33], in contrast to members of Poaceae, such as black oat and rye, which concentrates those compounds in their roots and other organs, for example, the grains [28], [34], [35]. Concerning to the periods when the samples were collected, species collected at 30 DAR segregated from those collected at 60, 80 and 100 DAS and 15 DAR (Figure 5B). The wavelengths that contributed the most for that segregation were 237, 242, 287, 297, 302 and 312 nm (Figure 6B). The overlapping of samples collected at different periods at the score plot PC2(+) indicate similarity between those and distinction from the samples collected at 30 DAR, which segregated at PC2(−) (Figure 5B), suggesting that black oat, rye and oilseed radish phenolic profile at 60, 80 and 100 DAS share more similarities then the profiles of these plants after rolling. This result due to the changes in the chemical composition of the plant material after longer time for sampling, 30 days after rolling, when plant material have longer period of degradation and the releasing of phenolic compounds [36]. Secondary metabolites profiles, such as phenolic compounds, vary in quality and quantity depending on many factors, including weather condition, plant age, and methods for extractions and analysis [37]. In a study on total phenolic compounds in rye [34], reported differences related to the phenological stage in the content of five phenolic acids, with the highest contents at 22 days after blooming and the lowest contents during grain maturation. Furthermore, some phenolic acids are probably related to lignification process, which occurs after rolling. Due to its lower C/N ratio and higher mineralization rate, the oilseed radish is broken down within 15 days after rolling. In contrast, rye and black oat, due to their higher fiber content, take around 20 days to be broken down [36], [38], [39]. On the whole, the three species have started breaking down 15 days after rolling. Therefore, the phenolic compounds with allelopathic potential detected in plant tissues at 60, 80 and 100 DAS may have been released into the soil [36] by many ways, such as leaching, volatilization and dry matter decomposition by soil microorganisms [40], leading to the spectral profile changes observed.

On the other hand, when samples were analyzed considering the management system, no discrimination between single crop or intercrop was observed (Figures 4 and 5C), except in the wavelengths 257, 263 and 267 nm (*p* < 0.05) (Figure 6C). The phenolic compounds produced by one plant species may interfere on the growth of other plant species, thus we aimed to investigate if intercropping would restrict one or both intercropped species, and if intercropping would result in distinct spectral profiles and phenolic compounds content. Our results suggest that intercropping species is a good option, as the sum of the phenolic compounds released by intercropped species could magnify their allelopathic potential, in addition to the physical barrier effect they play on the soil [36], [39], [41].

Besides PCA, supervised machine learning models PLS and knn were applied to spectral region 200–800 nm aiming to check the accuracy to classify samples according to species, time of cultivation and management system (Table 1). The prediction accuracy reached 75% when spectra were classified according to the species, which was higher than the accuracy for the classification according to the time of cultivation (62%) and the management system (52%) (Table 1). The wavelengths in the region of phenolic compounds’ absorption (200–400 nm) were the most relevant for that discrimination (Table 2). The results indicate that phenolic profiles are more distinguishable due to the species rather than phenological stage, time of cultivation or management system. By building predictive models such as PLS and knn, it is possible to assess how accurate an arbitrary previous classification is [42].

**Table 1:** Classification accuracy of predictive models PLS and knn according to plant species, time of cultivations and crop management system of UV–vis spectra from *Secale cereale* L, *Avena strigosa* L. and *Raphanus sativus* L. extracts.

**Accuracy**	**Classification**		
	**Species**	**Time**	**Management systems**
pls	75.68	66.19	56.46
kmn	72.23	67.84	52.98

**Table 2:** Individual relative contribution (%) of the 10 wavelengths, in UV–vis, most relevant to the classification by species, of the samples the cover crops *Avena strigosa* L. (black oat), *Secale cereale* L. (rye) and *Raphanus sativus* L. (oilseed radish).

**Wavelength (nm)**	**Black oat**	**Rye**	**Oilseed radish**
200	53.84	86.96	100.00
201	26.30	82.85	76.55
202	15.25	84.69	70.64
203	11.95	69.57	57.16
204	12.49	62.18	53.24
275	32.70	47.08	56.30
276	34.67	49.30	59.05
277	34.67	49.30	59.90
278	33.09	47.14	57.35
279	32.28	45.36	55.91

## 4 Conclusion

The analytical approach used in this work, i.e. the spectral data obtained via UV–vis spectroscopy in association with chemometric methods in R language with package specmine, enabled to discriminate and classify the samples based on their biochemical features.

The exploratory analysis of the spectra by multivariate analysis was efficient to show a trend of clustering between the treatments, discriminating the chemical profile of oilseed radish from black oat and rye.

Differences between the phenolic profiles were more evident regarding to the plant species rather than the period of cropping, phenologic stage or crop system, i.e. single or intercropped. This is corroborated by pls and knn results, which discriminated species with 75% accuracy, especially in the spectral region of phenolic compounds.

Regarding the periods of cropping, PCA analysis showed that the most evident discrimination occurred for rye and black oat at 30 days after rolling, in contrast to other sampling periods when black oat and rye were in the phenological stages of elongation, flowering and vegetative development, and the oilseed radish blossoming and grain ripening.
