# Supplementary material for: Exploratory and discriminant analysis of plant phenolic profiles obtained by UV–vis scanning spectroscopy
Source: J Integr Bioinform. 2021 Jun 4;18(3):20190056. doi: 10.1515/jib-2019-0056 (PMC8573236; doi:10.1515/jib-2019-0056)
Supplement: Supplementary file 1 [file jib-18-20190056-s001.docx]

FTIR (Pós-Doctorade data)

Souza,M

11 maio 2020

*Data analysis report (R Markdown) - Infrared Spectroscopic Data*

setwd("D:/UFSC/PNPD/NIR_Management")
sink("Management_PNPD_NIR")
#install.packages("specmine")
library(specmine)

#install.packages("https://cran.r-project.org/src/contrib/Archive/ChemoSpec/ChemoSpec_4.3.17.tar.gz", repos=NULL, type="source")
#install.packages("specmine")
library (gsubfn)
library(ChemoSpec)

#source("read_dx.R")
file.metadata = "metadata/metadata.csv"
description = " "
label.x = "Numero de onda cm-1"
label.val = "Absorbancia"


ir.management.ds = read_dataset_dx("data", filename.meta = file.metadata, type = "ir-spectra",
 description = description, label.x = label.x, label.values = label.val)

ir.management.ds = convert_to_factor(ir.management.ds,"Profundidade")
ir.management.ds = convert_to_factor(ir.management.ds,"Manejo")
ir.management.ds = convert_to_factor(ir.management.ds,"Especie")

Preliminary Inspection of Data

sum_dataset(ir.management.ds)

## Dataset summary:
## Valid dataset
## Description:
## Type of data: ir-spectra
## Number of samples: 1263
## Number of data points 1154
## Number of metadata variables: 3
## Label of x-axis values: Numero de onda cm-1
## Label of data points: Absorbancia
## Number of missing values in data: 0
## Mean of data values: 0.6953507
## Median of data values: 0.6345485
## Standard deviation: 0.2089858
## Range of values: 0.3864508 2.308383
## Quantiles:
## 0% 25% 50% 75% 100%
## 0.3864508 0.5408374 0.6345485 0.8337569 2.3083832

**Using full spectral data** Plotting the Spectra

plot_spectra_simple(ir.management.ds)


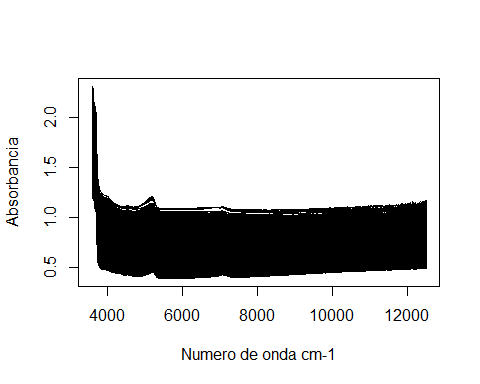


plot_spectra(ir.management.ds, "Profundidade", legend.place = "topright", cex = 0.76)


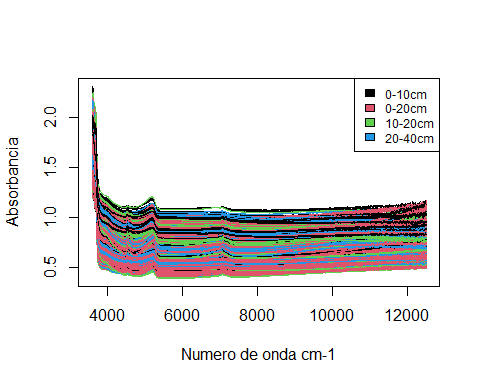


plot_spectra(ir.management.ds, "Manejo", legend.place = "topright", cex = 0.76)


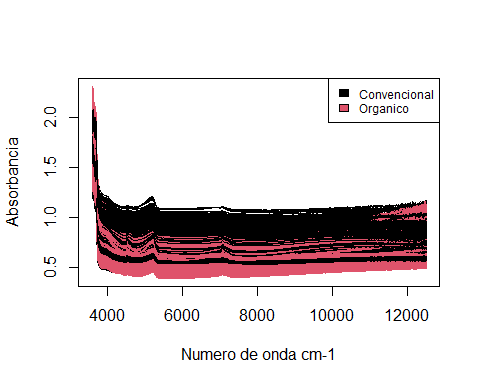


plot_spectra(ir.management.ds, "Especie", legend.place = "topright", cex = 0.76)


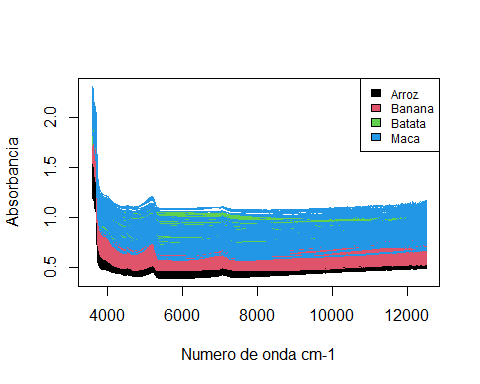


**UNIVARIATE ANALYSIS**

ir.anova = aov_all_vars(ir.management.ds,"Profundidade")
ir.anova[1:10,]
ir.anova = aov_all_vars(ir.management.ds,"Manejo")
ir.anova[1:10,]
ir.anova = aov_all_vars(ir.management.ds,"Especie")
ir.anova[1:10,]

**T test**

ir.convencional.organico = subset_samples_by_metadata_values(ir.management.ds, "Manejo", values = c("Convencional","Organico"))
ir.ttest = tTests_dataset(ir.convencional.organico, "Manejo")
ir.ttest[1:10,]

## p.value -log10 fdr
## 5947.72504128361 1.069980e-44 43.97062 5.746187e-43
## 5955.43934076323 1.079041e-44 43.96696 5.746187e-43
## 5940.01074180399 1.082447e-44 43.96559 5.746187e-43
## 5963.15364024284 1.100388e-44 43.95845 5.746187e-43
## 5932.29644232437 1.105821e-44 43.95632 5.746187e-43
## 5970.86793972246 1.119556e-44 43.95095 5.746187e-43
## 5924.58214284475 1.120817e-44 43.95047 5.746187e-43
## 5978.58223920208 1.131758e-44 43.94625 5.746187e-43
## 5916.86784336513 1.132047e-44 43.94614 5.746187e-43
## 5986.2965386817 1.146076e-44 43.94079 5.746187e-43

plot_ttests(ir.convencional.organico, ir.ttest, tt.threshold = 0.05)


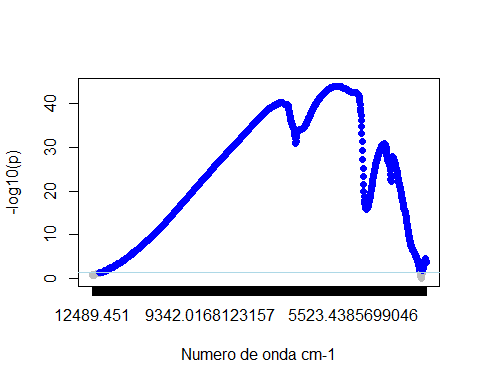


ir.convencional.organico = subset_samples_by_metadata_values(ir.management.ds, "Manejo", values = c("Convencional","Organico"))

ir.banana.batata = subset_samples_by_metadata_values(ir.management.ds, "Especie", values = c("Banana","Batata"))
ir.ttest = tTests_dataset(ir.banana.batata, "Especie")
ir.ttest[1:10,]

## p.value -log10 fdr
## 10059.4466639202 1.018884e-157 156.9919 1.186242e-155
## 10051.7323644406 1.395143e-157 156.8554 1.186242e-155
## 10067.1609633998 1.413678e-157 156.8496 1.186242e-155
## 10028.5894660017 1.650033e-157 156.7825 1.186242e-155
## 10020.8751665221 1.856637e-157 156.7313 1.186242e-155
## 9982.30366912402 1.971142e-157 156.7053 1.186242e-155
## 10036.3037654814 2.107725e-157 156.6762 1.186242e-155
## 9905.16067432784 2.186244e-157 156.6603 1.186242e-155
## 9990.01796860364 2.205073e-157 156.6566 1.186242e-155
## 10044.018064961 2.259248e-157 156.6460 1.186242e-155

plot_ttests(ir.banana.batata, ir.ttest, tt.threshold = 0.05)


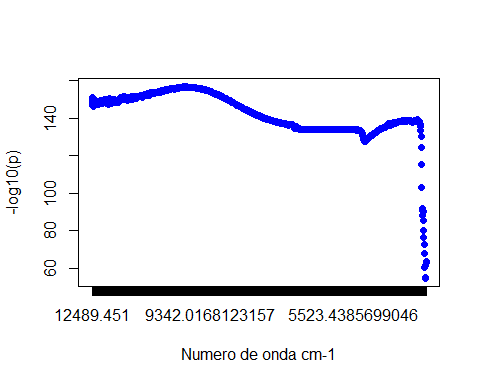


ir.banana.batata = subset_samples_by_metadata_values(ir.management.ds, "Especie", values = c("Banana","Batata"))

ir.maca.batata = subset_samples_by_metadata_values(ir.management.ds, "Especie", values = c("Maca","Batata"))
ir.ttest = tTests_dataset(ir.maca.batata, "Especie")
ir.ttest[1:10,]

## p.value -log10 fdr
## 3602.57799947962 4.060792e-97 96.39139 4.686153e-94
## 3594.8637 1.761298e-96 95.75417 1.016269e-93
## 3610.29229895924 1.277174e-94 93.89375 4.912862e-92
## 3633.43519739809 7.544386e-89 88.12238 2.176555e-86
## 3618.00659843885 1.297162e-88 87.88701 2.993850e-86
## 3641.14949687771 1.133379e-87 86.94562 2.179865e-85
## 3625.72089791847 1.927520e-87 86.71500 3.177654e-85
## 3648.86379635733 6.809324e-81 80.16690 9.822450e-79
## 3656.57809583695 2.829555e-72 71.54828 3.628118e-70
## 3664.29239531656 3.180152e-65 64.49755 3.669895e-63

plot_ttests(ir.banana.batata, ir.ttest, tt.threshold = 0.05)


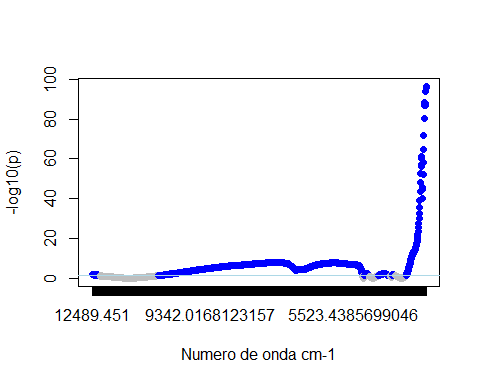


ir.maca.batata = subset_samples_by_metadata_values(ir.management.ds, "Especie", values = c("Maca","Batata"))

ir.banana.arroz = subset_samples_by_metadata_values(ir.management.ds, "Especie", values = c("Banana","Batata"))
ir.ttest = tTests_dataset(ir.banana.arroz, "Especie")
ir.ttest[1:10,]

## p.value -log10 fdr
## 10059.4466639202 1.018884e-157 156.9919 1.186242e-155
## 10051.7323644406 1.395143e-157 156.8554 1.186242e-155
## 10067.1609633998 1.413678e-157 156.8496 1.186242e-155
## 10028.5894660017 1.650033e-157 156.7825 1.186242e-155
## 10020.8751665221 1.856637e-157 156.7313 1.186242e-155
## 9982.30366912402 1.971142e-157 156.7053 1.186242e-155
## 10036.3037654814 2.107725e-157 156.6762 1.186242e-155
## 9905.16067432784 2.186244e-157 156.6603 1.186242e-155
## 9990.01796860364 2.205073e-157 156.6566 1.186242e-155
## 10044.018064961 2.259248e-157 156.6460 1.186242e-155

plot_ttests(ir.banana.arroz, ir.ttest, tt.threshold = 0.05)


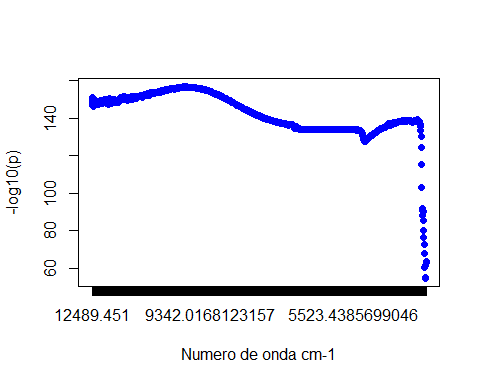


ir.banana.arroz = subset_samples_by_metadata_values(ir.management.ds, "Especie", values = c("Banana","Batata"))

**FoldChange Analysis with normalized data**

ir.fc = fold_change(ir.convencional.organico, "Manejo", ref.value = "Convencional")
plot_fold_change(ir.management.ds, ir.fc, fc.threshold = 2)


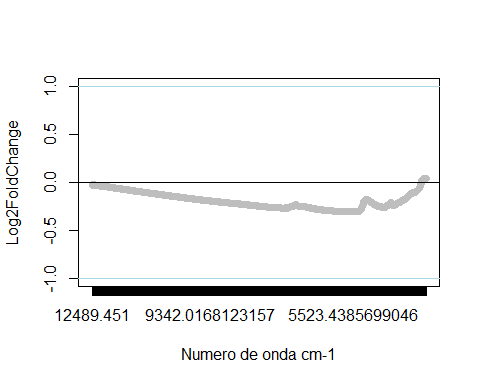


ir.fc = fold_change(ir.convencional.organico, "Manejo", ref.value = "Organico")
plot_fold_change(ir.management.ds, ir.fc, fc.threshold = 2)


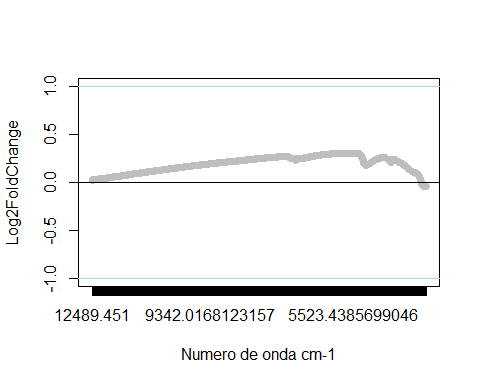


**Principal Components Analysis** Importance of components: Proportion of Variance explained in each component

ir.pca = pca_analysis_dataset(ir.management.ds)
summary(ir.pca, num.pcs = 10)

## Warning: In summary.prcomp(ir.pca, num.pcs = 10) :
## extra argument 'num.pcs' will be disregarded

## Importance of components:
## PC1 PC2 PC3 PC4 PC5 PC6 PC7
## Standard deviation 33.2753 5.59533 3.65018 0.9613 0.74012 0.46800 0.3334
## Proportion of Variance 0.9595 0.02713 0.01155 0.0008 0.00047 0.00019 0.0001
## Cumulative Proportion 0.9595 0.98661 0.99816 0.9990 0.99944 0.99962 0.9997
## PC8 PC9 PC10 PC11 PC12 PC13 PC14
## Standard deviation 0.31911 0.14038 0.11313 0.08341 0.08253 0.07973 0.07819
## Proportion of Variance 0.00009 0.00002 0.00001 0.00001 0.00001 0.00001 0.00001
## Cumulative Proportion 0.99981 0.99983 0.99984 0.99984 0.99985 0.99985 0.99986
## PC15 PC16 PC17 PC18 PC19 PC20 PC21
## Standard deviation 0.07474 0.07433 0.07129 0.06919 0.0681 0.06637 0.06472
## Proportion of Variance 0.00000 0.00000 0.00000 0.00000 0.0000 0.00000 0.00000
## Cumulative Proportion 0.99987 0.99987 0.99987 0.99988 0.9999 0.99989 0.99989
## PC22 PC23 PC24 PC25 PC26 PC27 PC28
## Standard deviation 0.06384 0.0603 0.05973 0.05887 0.0564 0.05483 0.05453
## Proportion of Variance 0.00000 0.0000 0.00000 0.00000 0.0000 0.00000 0.00000
## Cumulative Proportion 0.99989 0.9999 0.99990 0.99990 0.9999 0.99991 0.99991
## PC29 PC30 PC31 PC32 PC33 PC34 PC35
## Standard deviation 0.05426 0.05191 0.05141 0.05063 0.05037 0.04862 0.0483
## Proportion of Variance 0.00000 0.00000 0.00000 0.00000 0.00000 0.00000 0.0000
## Cumulative Proportion 0.99991 0.99992 0.99992 0.99992 0.99992 0.99992 0.9999
## PC36 PC37 PC38 PC39 PC40 PC41 PC42
## Standard deviation 0.04759 0.04665 0.04604 0.04494 0.04416 0.04391 0.04333
## Proportion of Variance 0.00000 0.00000 0.00000 0.00000 0.00000 0.00000 0.00000
## Cumulative Proportion 0.99993 0.99993 0.99993 0.99993 0.99994 0.99994 0.99994
## PC43 PC44 PC45 PC46 PC47 PC48 PC49
## Standard deviation 0.04241 0.04133 0.04011 0.03973 0.03927 0.03896 0.03854
## Proportion of Variance 0.00000 0.00000 0.00000 0.00000 0.00000 0.00000 0.00000
## Cumulative Proportion 0.99994 0.99994 0.99994 0.99994 0.99995 0.99995 0.99995
## PC50 PC51 PC52 PC53 PC54 PC55 PC56
## Standard deviation 0.03794 0.03742 0.03699 0.03602 0.03563 0.03509 0.03479
## Proportion of Variance 0.00000 0.00000 0.00000 0.00000 0.00000 0.00000 0.00000
## Cumulative Proportion 0.99995 0.99995 0.99995 0.99995 0.99995 0.99996 0.99996
## PC57 PC58 PC59 PC60 PC61 PC62 PC63
## Standard deviation 0.03427 0.03386 0.03333 0.0326 0.03222 0.03193 0.03165
## Proportion of Variance 0.00000 0.00000 0.00000 0.0000 0.00000 0.00000 0.00000
## Cumulative Proportion 0.99996 0.99996 0.99996 1.0000 0.99996 0.99996 0.99996
## PC64 PC65 PC66 PC67 PC68 PC69 PC70
## Standard deviation 0.03126 0.03073 0.03044 0.03012 0.02922 0.02916 0.02864
## Proportion of Variance 0.00000 0.00000 0.00000 0.00000 0.00000 0.00000 0.00000
## Cumulative Proportion 0.99996 0.99996 0.99997 0.99997 0.99997 0.99997 0.99997
## PC71 PC72 PC73 PC74 PC75 PC76 PC77
## Standard deviation 0.02843 0.02802 0.02764 0.02761 0.02707 0.02695 0.02631
## Proportion of Variance 0.00000 0.00000 0.00000 0.00000 0.00000 0.00000 0.00000
## Cumulative Proportion 0.99997 0.99997 0.99997 0.99997 0.99997 0.99997 0.99997
## PC78 PC79 PC80 PC81 PC82 PC83 PC84
## Standard deviation 0.02607 0.0258 0.02562 0.02546 0.02508 0.02454 0.02436
## Proportion of Variance 0.00000 0.0000 0.00000 0.00000 0.00000 0.00000 0.00000
## Cumulative Proportion 0.99997 1.0000 0.99997 0.99998 0.99998 0.99998 0.99998
## PC85 PC86 PC87 PC88 PC89 PC90 PC91
## Standard deviation 0.02416 0.02411 0.02348 0.02323 0.02259 0.02232 0.02213
## Proportion of Variance 0.00000 0.00000 0.00000 0.00000 0.00000 0.00000 0.00000
## Cumulative Proportion 0.99998 0.99998 0.99998 0.99998 0.99998 0.99998 0.99998
## PC92 PC93 PC94 PC95 PC96 PC97 PC98
## Standard deviation 0.02209 0.02194 0.02171 0.02136 0.0212 0.02091 0.02067
## Proportion of Variance 0.00000 0.00000 0.00000 0.00000 0.0000 0.00000 0.00000
## Cumulative Proportion 0.99998 0.99998 0.99998 0.99998 1.0000 0.99998 0.99998
## PC99 PC100 PC101 PC102 PC103 PC104 PC105
## Standard deviation 0.02041 0.01995 0.01956 0.0194 0.01922 0.01896 0.01872
## Proportion of Variance 0.00000 0.00000 0.00000 0.0000 0.00000 0.00000 0.00000
## Cumulative Proportion 0.99998 0.99998 0.99998 1.0000 0.99998 0.99998 0.99999
## PC106 PC107 PC108 PC109 PC110 PC111 PC112
## Standard deviation 0.01845 0.01836 0.01812 0.01785 0.01773 0.01746 0.0172
## Proportion of Variance 0.00000 0.00000 0.00000 0.00000 0.00000 0.00000 0.0000
## Cumulative Proportion 0.99999 0.99999 0.99999 0.99999 0.99999 0.99999 1.0000
## PC113 PC114 PC115 PC116 PC117 PC118 PC119
## Standard deviation 0.01709 0.01696 0.01683 0.01658 0.01646 0.01625 0.01605
## Proportion of Variance 0.00000 0.00000 0.00000 0.00000 0.00000 0.00000 0.00000
## Cumulative Proportion 0.99999 0.99999 0.99999 0.99999 0.99999 0.99999 0.99999
## PC120 PC121 PC122 PC123 PC124 PC125 PC126
## Standard deviation 0.01567 0.01555 0.01546 0.01536 0.015 0.01489 0.01483
## Proportion of Variance 0.00000 0.00000 0.00000 0.00000 0.000 0.00000 0.00000
## Cumulative Proportion 0.99999 0.99999 0.99999 0.99999 1.000 0.99999 0.99999
## PC127 PC128 PC129 PC130 PC131 PC132 PC133
## Standard deviation 0.01472 0.01461 0.01437 0.01429 0.01411 0.01408 0.01371
## Proportion of Variance 0.00000 0.00000 0.00000 0.00000 0.00000 0.00000 0.00000
## Cumulative Proportion 0.99999 0.99999 0.99999 0.99999 0.99999 0.99999 0.99999
## PC134 PC135 PC136 PC137 PC138 PC139 PC140
## Standard deviation 0.01358 0.01341 0.01329 0.01317 0.01297 0.0129 0.01273
## Proportion of Variance 0.00000 0.00000 0.00000 0.00000 0.00000 0.0000 0.00000
## Cumulative Proportion 0.99999 0.99999 0.99999 0.99999 0.99999 1.0000 0.99999
## PC141 PC142 PC143 PC144 PC145 PC146 PC147
## Standard deviation 0.01257 0.01236 0.01226 0.01207 0.01202 0.01199 0.01186
## Proportion of Variance 0.00000 0.00000 0.00000 0.00000 0.00000 0.00000 0.00000
## Cumulative Proportion 0.99999 0.99999 0.99999 0.99999 0.99999 0.99999 0.99999
## PC148 PC149 PC150 PC151 PC152 PC153 PC154
## Standard deviation 0.01167 0.01163 0.01148 0.01131 0.0112 0.01106 0.01099
## Proportion of Variance 0.00000 0.00000 0.00000 0.00000 0.0000 0.00000 0.00000
## Cumulative Proportion 0.99999 0.99999 0.99999 0.99999 1.0000 0.99999 0.99999
## PC155 PC156 PC157 PC158 PC159 PC160 PC161
## Standard deviation 0.01087 0.01071 0.01062 0.01053 0.01037 0.0103 0.01027
## Proportion of Variance 0.00000 0.00000 0.00000 0.00000 0.00000 0.0000 0.00000
## Cumulative Proportion 0.99999 0.99999 0.99999 0.99999 0.99999 1.0000 0.99999
## PC162 PC163 PC164 PC165 PC166 PC167
## Standard deviation 0.01013 0.01009 0.009949 0.009899 0.009766 0.009722
## Proportion of Variance 0.00000 0.00000 0.000000 0.000000 0.000000 0.000000
## Cumulative Proportion 0.99999 1.00000 1.000000 1.000000 1.000000 1.000000
## PC168 PC169 PC170 PC171 PC172 PC173
## Standard deviation 0.009592 0.009503 0.009428 0.009321 0.009283 0.009106
## Proportion of Variance 0.000000 0.000000 0.000000 0.000000 0.000000 0.000000
## Cumulative Proportion 1.000000 1.000000 1.000000 1.000000 1.000000 1.000000
## PC174 PC175 PC176 PC177 PC178 PC179
## Standard deviation 0.009051 0.008902 0.008869 0.008755 0.008732 0.008566
## Proportion of Variance 0.000000 0.000000 0.000000 0.000000 0.000000 0.000000
## Cumulative Proportion 1.000000 1.000000 1.000000 1.000000 1.000000 1.000000
## PC180 PC181 PC182 PC183 PC184 PC185
## Standard deviation 0.008506 0.008398 0.008295 0.008266 0.008184 0.008086
## Proportion of Variance 0.000000 0.000000 0.000000 0.000000 0.000000 0.000000
## Cumulative Proportion 1.000000 1.000000 1.000000 1.000000 1.000000 1.000000
## PC186 PC187 PC188 PC189 PC190 PC191
## Standard deviation 0.007998 0.007929 0.007882 0.007874 0.00771 0.007661
## Proportion of Variance 0.000000 0.000000 0.000000 0.000000 0.00000 0.000000
## Cumulative Proportion 1.000000 1.000000 1.000000 1.000000 1.00000 1.000000
## PC192 PC193 PC194 PC195 PC196 PC197
## Standard deviation 0.007645 0.007544 0.007484 0.007421 0.007356 0.007309
## Proportion of Variance 0.000000 0.000000 0.000000 0.000000 0.000000 0.000000
## Cumulative Proportion 1.000000 1.000000 1.000000 1.000000 1.000000 1.000000
## PC198 PC199 PC200 PC201 PC202 PC203
## Standard deviation 0.007231 0.007187 0.007085 0.007035 0.006985 0.00692
## Proportion of Variance 0.000000 0.000000 0.000000 0.000000 0.000000 0.00000
## Cumulative Proportion 1.000000 1.000000 1.000000 1.000000 1.000000 1.00000
## PC204 PC205 PC206 PC207 PC208 PC209
## Standard deviation 0.006807 0.006767 0.006686 0.006627 0.006616 0.006564
## Proportion of Variance 0.000000 0.000000 0.000000 0.000000 0.000000 0.000000
## Cumulative Proportion 1.000000 1.000000 1.000000 1.000000 1.000000 1.000000
## PC210 PC211 PC212 PC213 PC214 PC215
## Standard deviation 0.006549 0.006451 0.006408 0.006325 0.006312 0.006249
## Proportion of Variance 0.000000 0.000000 0.000000 0.000000 0.000000 0.000000
## Cumulative Proportion 1.000000 1.000000 1.000000 1.000000 1.000000 1.000000
## PC216 PC217 PC218 PC219 PC220 PC221
## Standard deviation 0.006144 0.006137 0.006042 0.005998 0.005935 0.005916
## Proportion of Variance 0.000000 0.000000 0.000000 0.000000 0.000000 0.000000
## Cumulative Proportion 1.000000 1.000000 1.000000 1.000000 1.000000 1.000000
## PC222 PC223 PC224 PC225 PC226 PC227
## Standard deviation 0.005893 0.00577 0.005729 0.00569 0.005655 0.005634
## Proportion of Variance 0.000000 0.00000 0.000000 0.00000 0.000000 0.000000
## Cumulative Proportion 1.000000 1.00000 1.000000 1.00000 1.000000 1.000000
## PC228 PC229 PC230 PC231 PC232 PC233
## Standard deviation 0.005603 0.005512 0.005462 0.005401 0.00537 0.005342
## Proportion of Variance 0.000000 0.000000 0.000000 0.000000 0.00000 0.000000
## Cumulative Proportion 1.000000 1.000000 1.000000 1.000000 1.00000 1.000000
## PC234 PC235 PC236 PC237 PC238 PC239
## Standard deviation 0.00529 0.005212 0.005162 0.005128 0.005068 0.00502
## Proportion of Variance 0.00000 0.000000 0.000000 0.000000 0.000000 0.00000
## Cumulative Proportion 1.00000 1.000000 1.000000 1.000000 1.000000 1.00000
## PC240 PC241 PC242 PC243 PC244 PC245
## Standard deviation 0.004986 0.004966 0.004942 0.004892 0.004835 0.004816
## Proportion of Variance 0.000000 0.000000 0.000000 0.000000 0.000000 0.000000
## Cumulative Proportion 1.000000 1.000000 1.000000 1.000000 1.000000 1.000000
## PC246 PC247 PC248 PC249 PC250 PC251
## Standard deviation 0.004774 0.004753 0.004692 0.004676 0.004636 0.004577
## Proportion of Variance 0.000000 0.000000 0.000000 0.000000 0.000000 0.000000
## Cumulative Proportion 1.000000 1.000000 1.000000 1.000000 1.000000 1.000000
## PC252 PC253 PC254 PC255 PC256 PC257
## Standard deviation 0.00454 0.004515 0.004502 0.004448 0.004428 0.004375
## Proportion of Variance 0.00000 0.000000 0.000000 0.000000 0.000000 0.000000
## Cumulative Proportion 1.00000 1.000000 1.000000 1.000000 1.000000 1.000000
## PC258 PC259 PC260 PC261 PC262 PC263
## Standard deviation 0.004326 0.004299 0.004229 0.004202 0.004173 0.004118
## Proportion of Variance 0.000000 0.000000 0.000000 0.000000 0.000000 0.000000
## Cumulative Proportion 1.000000 1.000000 1.000000 1.000000 1.000000 1.000000
## PC264 PC265 PC266 PC267 PC268 PC269
## Standard deviation 0.004099 0.004065 0.004032 0.004018 0.003995 0.003963
## Proportion of Variance 0.000000 0.000000 0.000000 0.000000 0.000000 0.000000
## Cumulative Proportion 1.000000 1.000000 1.000000 1.000000 1.000000 1.000000
## PC270 PC271 PC272 PC273 PC274 PC275
## Standard deviation 0.00393 0.003858 0.003825 0.003812 0.003772 0.00372
## Proportion of Variance 0.00000 0.000000 0.000000 0.000000 0.000000 0.00000
## Cumulative Proportion 1.00000 1.000000 1.000000 1.000000 1.000000 1.00000
## PC276 PC277 PC278 PC279 PC280 PC281
## Standard deviation 0.003717 0.003683 0.003677 0.003666 0.003615 0.00359
## Proportion of Variance 0.000000 0.000000 0.000000 0.000000 0.000000 0.00000
## Cumulative Proportion 1.000000 1.000000 1.000000 1.000000 1.000000 1.00000
## PC282 PC283 PC284 PC285 PC286 PC287
## Standard deviation 0.003543 0.003538 0.003535 0.003474 0.003463 0.003438
## Proportion of Variance 0.000000 0.000000 0.000000 0.000000 0.000000 0.000000
## Cumulative Proportion 1.000000 1.000000 1.000000 1.000000 1.000000 1.000000
## PC288 PC289 PC290 PC291 PC292 PC293
## Standard deviation 0.003425 0.003372 0.003348 0.003335 0.003315 0.003272
## Proportion of Variance 0.000000 0.000000 0.000000 0.000000 0.000000 0.000000
## Cumulative Proportion 1.000000 1.000000 1.000000 1.000000 1.000000 1.000000
## PC294 PC295 PC296 PC297 PC298 PC299
## Standard deviation 0.003253 0.003229 0.003202 0.003175 0.003169 0.003124
## Proportion of Variance 0.000000 0.000000 0.000000 0.000000 0.000000 0.000000
## Cumulative Proportion 1.000000 1.000000 1.000000 1.000000 1.000000 1.000000
## PC300 PC301 PC302 PC303 PC304 PC305
## Standard deviation 0.003101 0.00307 0.003046 0.003012 0.003002 0.002992
## Proportion of Variance 0.000000 0.00000 0.000000 0.000000 0.000000 0.000000
## Cumulative Proportion 1.000000 1.00000 1.000000 1.000000 1.000000 1.000000
## PC306 PC307 PC308 PC309 PC310 PC311
## Standard deviation 0.002958 0.002952 0.002927 0.002919 0.002881 0.002862
## Proportion of Variance 0.000000 0.000000 0.000000 0.000000 0.000000 0.000000
## Cumulative Proportion 1.000000 1.000000 1.000000 1.000000 1.000000 1.000000
## PC312 PC313 PC314 PC315 PC316 PC317
## Standard deviation 0.002849 0.002824 0.002804 0.002793 0.002781 0.00274
## Proportion of Variance 0.000000 0.000000 0.000000 0.000000 0.000000 0.00000
## Cumulative Proportion 1.000000 1.000000 1.000000 1.000000 1.000000 1.00000
## PC318 PC319 PC320 PC321 PC322 PC323
## Standard deviation 0.002721 0.002693 0.002672 0.002666 0.002652 0.002627
## Proportion of Variance 0.000000 0.000000 0.000000 0.000000 0.000000 0.000000
## Cumulative Proportion 1.000000 1.000000 1.000000 1.000000 1.000000 1.000000
## PC324 PC325 PC326 PC327 PC328 PC329
## Standard deviation 0.002614 0.002599 0.002581 0.002561 0.002552 0.002534
## Proportion of Variance 0.000000 0.000000 0.000000 0.000000 0.000000 0.000000
## Cumulative Proportion 1.000000 1.000000 1.000000 1.000000 1.000000 1.000000
## PC330 PC331 PC332 PC333 PC334 PC335
## Standard deviation 0.002495 0.002472 0.002455 0.002439 0.002428 0.002407
## Proportion of Variance 0.000000 0.000000 0.000000 0.000000 0.000000 0.000000
## Cumulative Proportion 1.000000 1.000000 1.000000 1.000000 1.000000 1.000000
## PC336 PC337 PC338 PC339 PC340 PC341
## Standard deviation 0.002386 0.00238 0.002371 0.002343 0.002334 0.002311
## Proportion of Variance 0.000000 0.00000 0.000000 0.000000 0.000000 0.000000
## Cumulative Proportion 1.000000 1.00000 1.000000 1.000000 1.000000 1.000000
## PC342 PC343 PC344 PC345 PC346 PC347
## Standard deviation 0.002289 0.002267 0.00225 0.002239 0.002223 0.002212
## Proportion of Variance 0.000000 0.000000 0.00000 0.000000 0.000000 0.000000
## Cumulative Proportion 1.000000 1.000000 1.00000 1.000000 1.000000 1.000000
## PC348 PC349 PC350 PC351 PC352 PC353
## Standard deviation 0.0022 0.002168 0.002149 0.002146 0.002131 0.002125
## Proportion of Variance 0.0000 0.000000 0.000000 0.000000 0.000000 0.000000
## Cumulative Proportion 1.0000 1.000000 1.000000 1.000000 1.000000 1.000000
## PC354 PC355 PC356 PC357 PC358 PC359
## Standard deviation 0.002112 0.002102 0.002091 0.002068 0.002052 0.002047
## Proportion of Variance 0.000000 0.000000 0.000000 0.000000 0.000000 0.000000
## Cumulative Proportion 1.000000 1.000000 1.000000 1.000000 1.000000 1.000000
## PC360 PC361 PC362 PC363 PC364 PC365
## Standard deviation 0.002031 0.002018 0.001995 0.001982 0.00197 0.001966
## Proportion of Variance 0.000000 0.000000 0.000000 0.000000 0.00000 0.000000
## Cumulative Proportion 1.000000 1.000000 1.000000 1.000000 1.00000 1.000000
## PC366 PC367 PC368 PC369 PC370 PC371
## Standard deviation 0.001934 0.001932 0.001923 0.001911 0.0019 0.001876
## Proportion of Variance 0.000000 0.000000 0.000000 0.000000 0.0000 0.000000
## Cumulative Proportion 1.000000 1.000000 1.000000 1.000000 1.0000 1.000000
## PC372 PC373 PC374 PC375 PC376 PC377
## Standard deviation 0.00187 0.001843 0.001832 0.00181 0.001799 0.00178
## Proportion of Variance 0.00000 0.000000 0.000000 0.00000 0.000000 0.00000
## Cumulative Proportion 1.00000 1.000000 1.000000 1.00000 1.000000 1.00000
## PC378 PC379 PC380 PC381 PC382 PC383
## Standard deviation 0.001767 0.001759 0.001751 0.001738 0.001727 0.001708
## Proportion of Variance 0.000000 0.000000 0.000000 0.000000 0.000000 0.000000
## Cumulative Proportion 1.000000 1.000000 1.000000 1.000000 1.000000 1.000000
## PC384 PC385 PC386 PC387 PC388 PC389
## Standard deviation 0.001704 0.001692 0.001681 0.001677 0.001667 0.001657
## Proportion of Variance 0.000000 0.000000 0.000000 0.000000 0.000000 0.000000
## Cumulative Proportion 1.000000 1.000000 1.000000 1.000000 1.000000 1.000000
## PC390 PC391 PC392 PC393 PC394 PC395
## Standard deviation 0.001635 0.001621 0.001617 0.001601 0.00159 0.001582
## Proportion of Variance 0.000000 0.000000 0.000000 0.000000 0.00000 0.000000
## Cumulative Proportion 1.000000 1.000000 1.000000 1.000000 1.00000 1.000000
## PC396 PC397 PC398 PC399 PC400 PC401
## Standard deviation 0.001568 0.001559 0.001549 0.001532 0.001524 0.00152
## Proportion of Variance 0.000000 0.000000 0.000000 0.000000 0.000000 0.00000
## Cumulative Proportion 1.000000 1.000000 1.000000 1.000000 1.000000 1.00000
## PC402 PC403 PC404 PC405 PC406 PC407
## Standard deviation 0.001506 0.001502 0.00149 0.001475 0.001458 0.001455
## Proportion of Variance 0.000000 0.000000 0.00000 0.000000 0.000000 0.000000
## Cumulative Proportion 1.000000 1.000000 1.00000 1.000000 1.000000 1.000000
## PC408 PC409 PC410 PC411 PC412 PC413
## Standard deviation 0.001441 0.001432 0.001426 0.001416 0.001398 0.001394
## Proportion of Variance 0.000000 0.000000 0.000000 0.000000 0.000000 0.000000
## Cumulative Proportion 1.000000 1.000000 1.000000 1.000000 1.000000 1.000000
## PC414 PC415 PC416 PC417 PC418 PC419
## Standard deviation 0.001382 0.001377 0.001372 0.001367 0.001351 0.001343
## Proportion of Variance 0.000000 0.000000 0.000000 0.000000 0.000000 0.000000
## Cumulative Proportion 1.000000 1.000000 1.000000 1.000000 1.000000 1.000000
## PC420 PC421 PC422 PC423 PC424 PC425
## Standard deviation 0.001337 0.001329 0.001321 0.001312 0.001308 0.001296
## Proportion of Variance 0.000000 0.000000 0.000000 0.000000 0.000000 0.000000
## Cumulative Proportion 1.000000 1.000000 1.000000 1.000000 1.000000 1.000000
## PC426 PC427 PC428 PC429 PC430 PC431
## Standard deviation 0.001282 0.001275 0.001264 0.001255 0.001244 0.001236
## Proportion of Variance 0.000000 0.000000 0.000000 0.000000 0.000000 0.000000
## Cumulative Proportion 1.000000 1.000000 1.000000 1.000000 1.000000 1.000000
## PC432 PC433 PC434 PC435 PC436 PC437
## Standard deviation 0.001233 0.001226 0.001219 0.00121 0.001201 0.001197
## Proportion of Variance 0.000000 0.000000 0.000000 0.00000 0.000000 0.000000
## Cumulative Proportion 1.000000 1.000000 1.000000 1.00000 1.000000 1.000000
## PC438 PC439 PC440 PC441 PC442 PC443
## Standard deviation 0.001191 0.001184 0.001178 0.001167 0.001156 0.001154
## Proportion of Variance 0.000000 0.000000 0.000000 0.000000 0.000000 0.000000
## Cumulative Proportion 1.000000 1.000000 1.000000 1.000000 1.000000 1.000000
## PC444 PC445 PC446 PC447 PC448 PC449
## Standard deviation 0.001145 0.001138 0.001132 0.001123 0.001111 0.001106
## Proportion of Variance 0.000000 0.000000 0.000000 0.000000 0.000000 0.000000
## Cumulative Proportion 1.000000 1.000000 1.000000 1.000000 1.000000 1.000000
## PC450 PC451 PC452 PC453 PC454 PC455
## Standard deviation 0.001095 0.001093 0.001087 0.001078 0.001072 0.001068
## Proportion of Variance 0.000000 0.000000 0.000000 0.000000 0.000000 0.000000
## Cumulative Proportion 1.000000 1.000000 1.000000 1.000000 1.000000 1.000000
## PC456 PC457 PC458 PC459 PC460 PC461
## Standard deviation 0.001057 0.001053 0.001043 0.001035 0.001031 0.001026
## Proportion of Variance 0.000000 0.000000 0.000000 0.000000 0.000000 0.000000
## Cumulative Proportion 1.000000 1.000000 1.000000 1.000000 1.000000 1.000000
## PC462 PC463 PC464 PC465 PC466 PC467
## Standard deviation 0.00102 0.001016 0.001001 0.000999 0.0009898 0.000984
## Proportion of Variance 0.00000 0.000000 0.000000 0.000000 0.0000000 0.000000
## Cumulative Proportion 1.00000 1.000000 1.000000 1.000000 1.0000000 1.000000
## PC468 PC469 PC470 PC471 PC472
## Standard deviation 0.0009828 0.0009765 0.0009707 0.000959 0.0009575
## Proportion of Variance 0.0000000 0.0000000 0.0000000 0.000000 0.0000000
## Cumulative Proportion 1.0000000 1.0000000 1.0000000 1.000000 1.0000000
## PC473 PC474 PC475 PC476 PC477
## Standard deviation 0.0009555 0.0009462 0.0009458 0.0009348 0.0009281
## Proportion of Variance 0.0000000 0.0000000 0.0000000 0.0000000 0.0000000
## Cumulative Proportion 1.0000000 1.0000000 1.0000000 1.0000000 1.0000000
## PC478 PC479 PC480 PC481 PC482
## Standard deviation 0.0009217 0.0009167 0.0009148 0.0009091 0.0008987
## Proportion of Variance 0.0000000 0.0000000 0.0000000 0.0000000 0.0000000
## Cumulative Proportion 1.0000000 1.0000000 1.0000000 1.0000000 1.0000000
## PC483 PC484 PC485 PC486 PC487
## Standard deviation 0.0008979 0.0008907 0.0008856 0.0008783 0.0008742
## Proportion of Variance 0.0000000 0.0000000 0.0000000 0.0000000 0.0000000
## Cumulative Proportion 1.0000000 1.0000000 1.0000000 1.0000000 1.0000000
## PC488 PC489 PC490 PC491 PC492
## Standard deviation 0.0008683 0.0008638 0.0008613 0.0008588 0.0008486
## Proportion of Variance 0.0000000 0.0000000 0.0000000 0.0000000 0.0000000
## Cumulative Proportion 1.0000000 1.0000000 1.0000000 1.0000000 1.0000000
## PC493 PC494 PC495 PC496 PC497
## Standard deviation 0.0008467 0.0008408 0.0008387 0.0008322 0.0008277
## Proportion of Variance 0.0000000 0.0000000 0.0000000 0.0000000 0.0000000
## Cumulative Proportion 1.0000000 1.0000000 1.0000000 1.0000000 1.0000000
## PC498 PC499 PC500 PC501 PC502
## Standard deviation 0.0008182 0.0008118 0.0008098 0.0008046 0.0008017
## Proportion of Variance 0.0000000 0.0000000 0.0000000 0.0000000 0.0000000
## Cumulative Proportion 1.0000000 1.0000000 1.0000000 1.0000000 1.0000000
## PC503 PC504 PC505 PC506 PC507
## Standard deviation 0.0007991 0.0007934 0.0007893 0.0007853 0.0007779
## Proportion of Variance 0.0000000 0.0000000 0.0000000 0.0000000 0.0000000
## Cumulative Proportion 1.0000000 1.0000000 1.0000000 1.0000000 1.0000000
## PC508 PC509 PC510 PC511 PC512
## Standard deviation 0.0007763 0.0007722 0.0007693 0.0007626 0.0007605
## Proportion of Variance 0.0000000 0.0000000 0.0000000 0.0000000 0.0000000
## Cumulative Proportion 1.0000000 1.0000000 1.0000000 1.0000000 1.0000000
## PC513 PC514 PC515 PC516 PC517
## Standard deviation 0.0007555 0.0007509 0.00075 0.0007442 0.0007389
## Proportion of Variance 0.0000000 0.0000000 0.00000 0.0000000 0.0000000
## Cumulative Proportion 1.0000000 1.0000000 1.00000 1.0000000 1.0000000
## PC518 PC519 PC520 PC521 PC522
## Standard deviation 0.0007355 0.0007284 0.0007249 0.0007195 0.0007167
## Proportion of Variance 0.0000000 0.0000000 0.0000000 0.0000000 0.0000000
## Cumulative Proportion 1.0000000 1.0000000 1.0000000 1.0000000 1.0000000
## PC523 PC524 PC525 PC526 PC527
## Standard deviation 0.0007141 0.0007081 0.0007047 0.0006986 0.0006954
## Proportion of Variance 0.0000000 0.0000000 0.0000000 0.0000000 0.0000000
## Cumulative Proportion 1.0000000 1.0000000 1.0000000 1.0000000 1.0000000
## PC528 PC529 PC530 PC531 PC532
## Standard deviation 0.0006929 0.0006922 0.0006855 0.0006811 0.0006773
## Proportion of Variance 0.0000000 0.0000000 0.0000000 0.0000000 0.0000000
## Cumulative Proportion 1.0000000 1.0000000 1.0000000 1.0000000 1.0000000
## PC533 PC534 PC535 PC536 PC537
## Standard deviation 0.0006757 0.0006734 0.0006669 0.0006649 0.0006599
## Proportion of Variance 0.0000000 0.0000000 0.0000000 0.0000000 0.0000000
## Cumulative Proportion 1.0000000 1.0000000 1.0000000 1.0000000 1.0000000
## PC538 PC539 PC540 PC541 PC542
## Standard deviation 0.0006583 0.0006569 0.0006469 0.0006437 0.0006419
## Proportion of Variance 0.0000000 0.0000000 0.0000000 0.0000000 0.0000000
## Cumulative Proportion 1.0000000 1.0000000 1.0000000 1.0000000 1.0000000
## PC543 PC544 PC545 PC546 PC547
## Standard deviation 0.0006366 0.0006314 0.0006294 0.0006239 0.0006204
## Proportion of Variance 0.0000000 0.0000000 0.0000000 0.0000000 0.0000000
## Cumulative Proportion 1.0000000 1.0000000 1.0000000 1.0000000 1.0000000
## PC548 PC549 PC550 PC551 PC552
## Standard deviation 0.0006142 0.0006115 0.0006088 0.0006067 0.0005999
## Proportion of Variance 0.0000000 0.0000000 0.0000000 0.0000000 0.0000000
## Cumulative Proportion 1.0000000 1.0000000 1.0000000 1.0000000 1.0000000
## PC553 PC554 PC555 PC556 PC557
## Standard deviation 0.0005973 0.0005928 0.0005897 0.000583 0.000581
## Proportion of Variance 0.0000000 0.0000000 0.0000000 0.000000 0.000000
## Cumulative Proportion 1.0000000 1.0000000 1.0000000 1.000000 1.000000
## PC558 PC559 PC560 PC561 PC562
## Standard deviation 0.0005785 0.0005778 0.0005722 0.0005708 0.0005663
## Proportion of Variance 0.0000000 0.0000000 0.0000000 0.0000000 0.0000000
## Cumulative Proportion 1.0000000 1.0000000 1.0000000 1.0000000 1.0000000
## PC563 PC564 PC565 PC566 PC567
## Standard deviation 0.0005644 0.000561 0.0005599 0.0005553 0.0005534
## Proportion of Variance 0.0000000 0.000000 0.0000000 0.0000000 0.0000000
## Cumulative Proportion 1.0000000 1.000000 1.0000000 1.0000000 1.0000000
## PC568 PC569 PC570 PC571 PC572
## Standard deviation 0.0005517 0.000544 0.0005395 0.0005381 0.0005302
## Proportion of Variance 0.0000000 0.000000 0.0000000 0.0000000 0.0000000
## Cumulative Proportion 1.0000000 1.000000 1.0000000 1.0000000 1.0000000
## PC573 PC574 PC575 PC576 PC577
## Standard deviation 0.0005275 0.0005259 0.000523 0.0005209 0.0005185
## Proportion of Variance 0.0000000 0.0000000 0.000000 0.0000000 0.0000000
## Cumulative Proportion 1.0000000 1.0000000 1.000000 1.0000000 1.0000000
## PC578 PC579 PC580 PC581 PC582
## Standard deviation 0.0005147 0.0005105 0.000508 0.0005065 0.0005005
## Proportion of Variance 0.0000000 0.0000000 0.000000 0.0000000 0.0000000
## Cumulative Proportion 1.0000000 1.0000000 1.000000 1.0000000 1.0000000
## PC583 PC584 PC585 PC586 PC587
## Standard deviation 0.0004977 0.0004965 0.0004931 0.0004909 0.0004873
## Proportion of Variance 0.0000000 0.0000000 0.0000000 0.0000000 0.0000000
## Cumulative Proportion 1.0000000 1.0000000 1.0000000 1.0000000 1.0000000
## PC588 PC589 PC590 PC591 PC592
## Standard deviation 0.0004846 0.0004832 0.0004793 0.0004773 0.0004732
## Proportion of Variance 0.0000000 0.0000000 0.0000000 0.0000000 0.0000000
## Cumulative Proportion 1.0000000 1.0000000 1.0000000 1.0000000 1.0000000
## PC593 PC594 PC595 PC596 PC597
## Standard deviation 0.0004685 0.0004672 0.0004643 0.0004623 0.0004608
## Proportion of Variance 0.0000000 0.0000000 0.0000000 0.0000000 0.0000000
## Cumulative Proportion 1.0000000 1.0000000 1.0000000 1.0000000 1.0000000
## PC598 PC599 PC600 PC601 PC602
## Standard deviation 0.000454 0.0004534 0.0004486 0.0004438 0.0004407
## Proportion of Variance 0.000000 0.0000000 0.0000000 0.0000000 0.0000000
## Cumulative Proportion 1.000000 1.0000000 1.0000000 1.0000000 1.0000000
## PC603 PC604 PC605 PC606 PC607
## Standard deviation 0.0004393 0.0004374 0.0004348 0.0004327 0.0004292
## Proportion of Variance 0.0000000 0.0000000 0.0000000 0.0000000 0.0000000
## Cumulative Proportion 1.0000000 1.0000000 1.0000000 1.0000000 1.0000000
## PC608 PC609 PC610 PC611 PC612
## Standard deviation 0.0004267 0.0004235 0.0004228 0.0004198 0.0004171
## Proportion of Variance 0.0000000 0.0000000 0.0000000 0.0000000 0.0000000
## Cumulative Proportion 1.0000000 1.0000000 1.0000000 1.0000000 1.0000000
## PC613 PC614 PC615 PC616 PC617
## Standard deviation 0.0004145 0.0004118 0.00041 0.0004049 0.0004035
## Proportion of Variance 0.0000000 0.0000000 0.00000 0.0000000 0.0000000
## Cumulative Proportion 1.0000000 1.0000000 1.00000 1.0000000 1.0000000
## PC618 PC619 PC620 PC621 PC622
## Standard deviation 0.0003983 0.0003959 0.0003949 0.0003915 0.0003904
## Proportion of Variance 0.0000000 0.0000000 0.0000000 0.0000000 0.0000000
## Cumulative Proportion 1.0000000 1.0000000 1.0000000 1.0000000 1.0000000
## PC623 PC624 PC625 PC626 PC627
## Standard deviation 0.0003891 0.0003867 0.0003854 0.0003827 0.0003809
## Proportion of Variance 0.0000000 0.0000000 0.0000000 0.0000000 0.0000000
## Cumulative Proportion 1.0000000 1.0000000 1.0000000 1.0000000 1.0000000
## PC628 PC629 PC630 PC631 PC632
## Standard deviation 0.0003761 0.0003734 0.0003714 0.0003684 0.0003663
## Proportion of Variance 0.0000000 0.0000000 0.0000000 0.0000000 0.0000000
## Cumulative Proportion 1.0000000 1.0000000 1.0000000 1.0000000 1.0000000
## PC633 PC634 PC635 PC636 PC637
## Standard deviation 0.0003633 0.0003592 0.0003563 0.0003545 0.0003529
## Proportion of Variance 0.0000000 0.0000000 0.0000000 0.0000000 0.0000000
## Cumulative Proportion 1.0000000 1.0000000 1.0000000 1.0000000 1.0000000
## PC638 PC639 PC640 PC641 PC642
## Standard deviation 0.0003516 0.0003475 0.0003458 0.0003454 0.0003417
## Proportion of Variance 0.0000000 0.0000000 0.0000000 0.0000000 0.0000000
## Cumulative Proportion 1.0000000 1.0000000 1.0000000 1.0000000 1.0000000
## PC643 PC644 PC645 PC646 PC647
## Standard deviation 0.0003392 0.000338 0.000334 0.0003335 0.0003315
## Proportion of Variance 0.0000000 0.000000 0.000000 0.0000000 0.0000000
## Cumulative Proportion 1.0000000 1.000000 1.000000 1.0000000 1.0000000
## PC648 PC649 PC650 PC651 PC652
## Standard deviation 0.0003283 0.0003279 0.0003243 0.0003231 0.0003196
## Proportion of Variance 0.0000000 0.0000000 0.0000000 0.0000000 0.0000000
## Cumulative Proportion 1.0000000 1.0000000 1.0000000 1.0000000 1.0000000
## PC653 PC654 PC655 PC656 PC657
## Standard deviation 0.0003187 0.0003178 0.0003153 0.0003114 0.0003106
## Proportion of Variance 0.0000000 0.0000000 0.0000000 0.0000000 0.0000000
## Cumulative Proportion 1.0000000 1.0000000 1.0000000 1.0000000 1.0000000
## PC658 PC659 PC660 PC661 PC662
## Standard deviation 0.0003096 0.0003046 0.0003035 0.0003003 0.0002946
## Proportion of Variance 0.0000000 0.0000000 0.0000000 0.0000000 0.0000000
## Cumulative Proportion 1.0000000 1.0000000 1.0000000 1.0000000 1.0000000
## PC663 PC664 PC665 PC666 PC667
## Standard deviation 0.0002939 0.0002934 0.0002902 0.0002894 0.0002883
## Proportion of Variance 0.0000000 0.0000000 0.0000000 0.0000000 0.0000000
## Cumulative Proportion 1.0000000 1.0000000 1.0000000 1.0000000 1.0000000
## PC668 PC669 PC670 PC671 PC672
## Standard deviation 0.0002859 0.000283 0.0002805 0.0002784 0.0002754
## Proportion of Variance 0.0000000 0.000000 0.0000000 0.0000000 0.0000000
## Cumulative Proportion 1.0000000 1.000000 1.0000000 1.0000000 1.0000000
## PC673 PC674 PC675 PC676 PC677
## Standard deviation 0.0002744 0.0002727 0.0002702 0.0002695 0.0002675
## Proportion of Variance 0.0000000 0.0000000 0.0000000 0.0000000 0.0000000
## Cumulative Proportion 1.0000000 1.0000000 1.0000000 1.0000000 1.0000000
## PC678 PC679 PC680 PC681 PC682
## Standard deviation 0.0002663 0.0002645 0.0002626 0.0002606 0.0002574
## Proportion of Variance 0.0000000 0.0000000 0.0000000 0.0000000 0.0000000
## Cumulative Proportion 1.0000000 1.0000000 1.0000000 1.0000000 1.0000000
## PC683 PC684 PC685 PC686 PC687
## Standard deviation 0.0002553 0.0002528 0.0002521 0.0002497 0.0002478
## Proportion of Variance 0.0000000 0.0000000 0.0000000 0.0000000 0.0000000
## Cumulative Proportion 1.0000000 1.0000000 1.0000000 1.0000000 1.0000000
## PC688 PC689 PC690 PC691 PC692
## Standard deviation 0.0002457 0.0002447 0.0002434 0.0002411 0.0002399
## Proportion of Variance 0.0000000 0.0000000 0.0000000 0.0000000 0.0000000
## Cumulative Proportion 1.0000000 1.0000000 1.0000000 1.0000000 1.0000000
## PC693 PC694 PC695 PC696 PC697
## Standard deviation 0.0002376 0.0002362 0.0002343 0.000233 0.0002319
## Proportion of Variance 0.0000000 0.0000000 0.0000000 0.000000 0.0000000
## Cumulative Proportion 1.0000000 1.0000000 1.0000000 1.000000 1.0000000
## PC698 PC699 PC700 PC701 PC702
## Standard deviation 0.000228 0.0002267 0.0002221 0.0002217 0.0002189
## Proportion of Variance 0.000000 0.0000000 0.0000000 0.0000000 0.0000000
## Cumulative Proportion 1.000000 1.0000000 1.0000000 1.0000000 1.0000000
## PC703 PC704 PC705 PC706 PC707
## Standard deviation 0.0002184 0.0002168 0.000216 0.0002131 0.0002126
## Proportion of Variance 0.0000000 0.0000000 0.000000 0.0000000 0.0000000
## Cumulative Proportion 1.0000000 1.0000000 1.000000 1.0000000 1.0000000
## PC708 PC709 PC710 PC711 PC712 PC713
## Standard deviation 0.000211 0.0002096 0.000209 0.000205 0.0002038 0.0002023
## Proportion of Variance 0.000000 0.0000000 0.000000 0.000000 0.0000000 0.0000000
## Cumulative Proportion 1.000000 1.0000000 1.000000 1.000000 1.0000000 1.0000000
## PC714 PC715 PC716 PC717 PC718
## Standard deviation 0.000199 0.0001985 0.0001977 0.0001938 0.0001931
## Proportion of Variance 0.000000 0.0000000 0.0000000 0.0000000 0.0000000
## Cumulative Proportion 1.000000 1.0000000 1.0000000 1.0000000 1.0000000
## PC719 PC720 PC721 PC722 PC723 PC724
## Standard deviation 0.0001923 0.000191 0.0001897 0.000186 0.000185 0.0001843
## Proportion of Variance 0.0000000 0.000000 0.0000000 0.000000 0.000000 0.0000000
## Cumulative Proportion 1.0000000 1.000000 1.0000000 1.000000 1.000000 1.0000000
## PC725 PC726 PC727 PC728 PC729
## Standard deviation 0.0001819 0.0001797 0.0001792 0.0001772 0.000176
## Proportion of Variance 0.0000000 0.0000000 0.0000000 0.0000000 0.000000
## Cumulative Proportion 1.0000000 1.0000000 1.0000000 1.0000000 1.000000
## PC730 PC731 PC732 PC733 PC734
## Standard deviation 0.0001733 0.0001714 0.0001709 0.00017 0.0001691
## Proportion of Variance 0.0000000 0.0000000 0.0000000 0.00000 0.0000000
## Cumulative Proportion 1.0000000 1.0000000 1.0000000 1.00000 1.0000000
## PC735 PC736 PC737 PC738 PC739
## Standard deviation 0.0001669 0.0001651 0.0001639 0.000162 0.0001609
## Proportion of Variance 0.0000000 0.0000000 0.0000000 0.000000 0.0000000
## Cumulative Proportion 1.0000000 1.0000000 1.0000000 1.000000 1.0000000
## PC740 PC741 PC742 PC743 PC744
## Standard deviation 0.0001595 0.0001585 0.0001571 0.0001557 0.0001548
## Proportion of Variance 0.0000000 0.0000000 0.0000000 0.0000000 0.0000000
## Cumulative Proportion 1.0000000 1.0000000 1.0000000 1.0000000 1.0000000
## PC745 PC746 PC747 PC748 PC749
## Standard deviation 0.0001534 0.0001512 0.0001502 0.0001493 0.0001488
## Proportion of Variance 0.0000000 0.0000000 0.0000000 0.0000000 0.0000000
## Cumulative Proportion 1.0000000 1.0000000 1.0000000 1.0000000 1.0000000
## PC750 PC751 PC752 PC753 PC754
## Standard deviation 0.000146 0.0001447 0.0001437 0.0001415 0.0001413
## Proportion of Variance 0.000000 0.0000000 0.0000000 0.0000000 0.0000000
## Cumulative Proportion 1.000000 1.0000000 1.0000000 1.0000000 1.0000000
## PC755 PC756 PC757 PC758 PC759
## Standard deviation 0.0001395 0.0001379 0.0001355 0.0001351 0.0001342
## Proportion of Variance 0.0000000 0.0000000 0.0000000 0.0000000 0.0000000
## Cumulative Proportion 1.0000000 1.0000000 1.0000000 1.0000000 1.0000000
## PC760 PC761 PC762 PC763 PC764
## Standard deviation 0.0001337 0.0001319 0.0001312 0.0001291 0.0001287
## Proportion of Variance 0.0000000 0.0000000 0.0000000 0.0000000 0.0000000
## Cumulative Proportion 1.0000000 1.0000000 1.0000000 1.0000000 1.0000000
## PC765 PC766 PC767 PC768 PC769
## Standard deviation 0.000127 0.0001256 0.0001252 0.0001233 0.0001226
## Proportion of Variance 0.000000 0.0000000 0.0000000 0.0000000 0.0000000
## Cumulative Proportion 1.000000 1.0000000 1.0000000 1.0000000 1.0000000
## PC770 PC771 PC772 PC773 PC774
## Standard deviation 0.0001215 0.0001206 0.0001179 0.0001171 0.0001146
## Proportion of Variance 0.0000000 0.0000000 0.0000000 0.0000000 0.0000000
## Cumulative Proportion 1.0000000 1.0000000 1.0000000 1.0000000 1.0000000
## PC775 PC776 PC777 PC778 PC779
## Standard deviation 0.0001135 0.000113 0.0001116 0.000111 0.0001093
## Proportion of Variance 0.0000000 0.000000 0.0000000 0.000000 0.0000000
## Cumulative Proportion 1.0000000 1.000000 1.0000000 1.000000 1.0000000
## PC780 PC781 PC782 PC783 PC784
## Standard deviation 0.0001084 0.000108 0.0001069 0.0001058 0.0001054
## Proportion of Variance 0.0000000 0.000000 0.0000000 0.0000000 0.0000000
## Cumulative Proportion 1.0000000 1.000000 1.0000000 1.0000000 1.0000000
## PC785 PC786 PC787 PC788 PC789
## Standard deviation 0.0001045 0.0001033 0.0001027 0.0001009 9.957e-05
## Proportion of Variance 0.0000000 0.0000000 0.0000000 0.0000000 0.000e+00
## Cumulative Proportion 1.0000000 1.0000000 1.0000000 1.0000000 1.000e+00
## PC790 PC791 PC792 PC793 PC794
## Standard deviation 9.802e-05 9.763e-05 9.643e-05 9.557e-05 9.481e-05
## Proportion of Variance 0.000e+00 0.000e+00 0.000e+00 0.000e+00 0.000e+00
## Cumulative Proportion 1.000e+00 1.000e+00 1.000e+00 1.000e+00 1.000e+00
## PC795 PC796 PC797 PC798 PC799
## Standard deviation 9.378e-05 9.295e-05 9.204e-05 9.121e-05 9.036e-05
## Proportion of Variance 0.000e+00 0.000e+00 0.000e+00 0.000e+00 0.000e+00
## Cumulative Proportion 1.000e+00 1.000e+00 1.000e+00 1.000e+00 1.000e+00
## PC800 PC801 PC802 PC803 PC804
## Standard deviation 8.885e-05 8.821e-05 8.748e-05 8.637e-05 8.481e-05
## Proportion of Variance 0.000e+00 0.000e+00 0.000e+00 0.000e+00 0.000e+00
## Cumulative Proportion 1.000e+00 1.000e+00 1.000e+00 1.000e+00 1.000e+00
## PC805 PC806 PC807 PC808 PC809
## Standard deviation 8.435e-05 8.37e-05 8.308e-05 8.224e-05 8.139e-05
## Proportion of Variance 0.000e+00 0.00e+00 0.000e+00 0.000e+00 0.000e+00
## Cumulative Proportion 1.000e+00 1.00e+00 1.000e+00 1.000e+00 1.000e+00
## PC810 PC811 PC812 PC813 PC814
## Standard deviation 8.083e-05 7.971e-05 7.851e-05 7.78e-05 7.628e-05
## Proportion of Variance 0.000e+00 0.000e+00 0.000e+00 0.00e+00 0.000e+00
## Cumulative Proportion 1.000e+00 1.000e+00 1.000e+00 1.00e+00 1.000e+00
## PC815 PC816 PC817 PC818 PC819
## Standard deviation 7.561e-05 7.454e-05 7.368e-05 7.265e-05 7.18e-05
## Proportion of Variance 0.000e+00 0.000e+00 0.000e+00 0.000e+00 0.00e+00
## Cumulative Proportion 1.000e+00 1.000e+00 1.000e+00 1.000e+00 1.00e+00
## PC820 PC821 PC822 PC823 PC824
## Standard deviation 7.129e-05 7.021e-05 6.967e-05 6.866e-05 6.816e-05
## Proportion of Variance 0.000e+00 0.000e+00 0.000e+00 0.000e+00 0.000e+00
## Cumulative Proportion 1.000e+00 1.000e+00 1.000e+00 1.000e+00 1.000e+00
## PC825 PC826 PC827 PC828 PC829
## Standard deviation 6.744e-05 6.71e-05 6.584e-05 6.529e-05 6.454e-05
## Proportion of Variance 0.000e+00 0.00e+00 0.000e+00 0.000e+00 0.000e+00
## Cumulative Proportion 1.000e+00 1.00e+00 1.000e+00 1.000e+00 1.000e+00
## PC830 PC831 PC832 PC833 PC834
## Standard deviation 6.381e-05 6.304e-05 6.242e-05 6.199e-05 6.104e-05
## Proportion of Variance 0.000e+00 0.000e+00 0.000e+00 0.000e+00 0.000e+00
## Cumulative Proportion 1.000e+00 1.000e+00 1.000e+00 1.000e+00 1.000e+00
## PC835 PC836 PC837 PC838 PC839
## Standard deviation 6.055e-05 5.973e-05 5.903e-05 5.889e-05 5.791e-05
## Proportion of Variance 0.000e+00 0.000e+00 0.000e+00 0.000e+00 0.000e+00
## Cumulative Proportion 1.000e+00 1.000e+00 1.000e+00 1.000e+00 1.000e+00
## PC840 PC841 PC842 PC843 PC844
## Standard deviation 5.776e-05 5.713e-05 5.61e-05 5.473e-05 5.409e-05
## Proportion of Variance 0.000e+00 0.000e+00 0.00e+00 0.000e+00 0.000e+00
## Cumulative Proportion 1.000e+00 1.000e+00 1.00e+00 1.000e+00 1.000e+00
## PC845 PC846 PC847 PC848 PC849
## Standard deviation 5.379e-05 5.307e-05 5.247e-05 5.224e-05 5.211e-05
## Proportion of Variance 0.000e+00 0.000e+00 0.000e+00 0.000e+00 0.000e+00
## Cumulative Proportion 1.000e+00 1.000e+00 1.000e+00 1.000e+00 1.000e+00
## PC850 PC851 PC852 PC853 PC854
## Standard deviation 5.077e-05 5.035e-05 4.95e-05 4.821e-05 4.794e-05
## Proportion of Variance 0.000e+00 0.000e+00 0.00e+00 0.000e+00 0.000e+00
## Cumulative Proportion 1.000e+00 1.000e+00 1.00e+00 1.000e+00 1.000e+00
## PC855 PC856 PC857 PC858 PC859
## Standard deviation 4.771e-05 4.732e-05 4.643e-05 4.564e-05 4.513e-05
## Proportion of Variance 0.000e+00 0.000e+00 0.000e+00 0.000e+00 0.000e+00
## Cumulative Proportion 1.000e+00 1.000e+00 1.000e+00 1.000e+00 1.000e+00
## PC860 PC861 PC862 PC863 PC864
## Standard deviation 4.483e-05 4.432e-05 4.402e-05 4.333e-05 4.239e-05
## Proportion of Variance 0.000e+00 0.000e+00 0.000e+00 0.000e+00 0.000e+00
## Cumulative Proportion 1.000e+00 1.000e+00 1.000e+00 1.000e+00 1.000e+00
## PC865 PC866 PC867 PC868 PC869
## Standard deviation 4.208e-05 4.199e-05 4.12e-05 4.05e-05 4.036e-05
## Proportion of Variance 0.000e+00 0.000e+00 0.00e+00 0.00e+00 0.000e+00
## Cumulative Proportion 1.000e+00 1.000e+00 1.00e+00 1.00e+00 1.000e+00
## PC870 PC871 PC872 PC873 PC874
## Standard deviation 3.938e-05 3.881e-05 3.858e-05 3.797e-05 3.689e-05
## Proportion of Variance 0.000e+00 0.000e+00 0.000e+00 0.000e+00 0.000e+00
## Cumulative Proportion 1.000e+00 1.000e+00 1.000e+00 1.000e+00 1.000e+00
## PC875 PC876 PC877 PC878 PC879
## Standard deviation 3.648e-05 3.641e-05 3.597e-05 3.559e-05 3.508e-05
## Proportion of Variance 0.000e+00 0.000e+00 0.000e+00 0.000e+00 0.000e+00
## Cumulative Proportion 1.000e+00 1.000e+00 1.000e+00 1.000e+00 1.000e+00
## PC880 PC881 PC882 PC883 PC884
## Standard deviation 3.413e-05 3.392e-05 3.351e-05 3.308e-05 3.288e-05
## Proportion of Variance 0.000e+00 0.000e+00 0.000e+00 0.000e+00 0.000e+00
## Cumulative Proportion 1.000e+00 1.000e+00 1.000e+00 1.000e+00 1.000e+00
## PC885 PC886 PC887 PC888 PC889
## Standard deviation 3.231e-05 3.195e-05 3.161e-05 3.081e-05 3.061e-05
## Proportion of Variance 0.000e+00 0.000e+00 0.000e+00 0.000e+00 0.000e+00
## Cumulative Proportion 1.000e+00 1.000e+00 1.000e+00 1.000e+00 1.000e+00
## PC890 PC891 PC892 PC893 PC894
## Standard deviation 3.01e-05 3.007e-05 2.956e-05 2.945e-05 2.869e-05
## Proportion of Variance 0.00e+00 0.000e+00 0.000e+00 0.000e+00 0.000e+00
## Cumulative Proportion 1.00e+00 1.000e+00 1.000e+00 1.000e+00 1.000e+00
## PC895 PC896 PC897 PC898 PC899
## Standard deviation 2.839e-05 2.774e-05 2.739e-05 2.7e-05 2.656e-05
## Proportion of Variance 0.000e+00 0.000e+00 0.000e+00 0.0e+00 0.000e+00
## Cumulative Proportion 1.000e+00 1.000e+00 1.000e+00 1.0e+00 1.000e+00
## PC900 PC901 PC902 PC903 PC904
## Standard deviation 2.649e-05 2.604e-05 2.547e-05 2.508e-05 2.494e-05
## Proportion of Variance 0.000e+00 0.000e+00 0.000e+00 0.000e+00 0.000e+00
## Cumulative Proportion 1.000e+00 1.000e+00 1.000e+00 1.000e+00 1.000e+00
## PC905 PC906 PC907 PC908 PC909
## Standard deviation 2.462e-05 2.418e-05 2.363e-05 2.323e-05 2.288e-05
## Proportion of Variance 0.000e+00 0.000e+00 0.000e+00 0.000e+00 0.000e+00
## Cumulative Proportion 1.000e+00 1.000e+00 1.000e+00 1.000e+00 1.000e+00
## PC910 PC911 PC912 PC913 PC914
## Standard deviation 2.232e-05 2.219e-05 2.173e-05 2.134e-05 2.109e-05
## Proportion of Variance 0.000e+00 0.000e+00 0.000e+00 0.000e+00 0.000e+00
## Cumulative Proportion 1.000e+00 1.000e+00 1.000e+00 1.000e+00 1.000e+00
## PC915 PC916 PC917 PC918 PC919
## Standard deviation 2.084e-05 2.052e-05 2.036e-05 2.014e-05 2.012e-05
## Proportion of Variance 0.000e+00 0.000e+00 0.000e+00 0.000e+00 0.000e+00
## Cumulative Proportion 1.000e+00 1.000e+00 1.000e+00 1.000e+00 1.000e+00
## PC920 PC921 PC922 PC923 PC924
## Standard deviation 1.95e-05 1.887e-05 1.88e-05 1.826e-05 1.795e-05
## Proportion of Variance 0.00e+00 0.000e+00 0.00e+00 0.000e+00 0.000e+00
## Cumulative Proportion 1.00e+00 1.000e+00 1.00e+00 1.000e+00 1.000e+00
## PC925 PC926 PC927 PC928 PC929
## Standard deviation 1.777e-05 1.748e-05 1.718e-05 1.686e-05 1.674e-05
## Proportion of Variance 0.000e+00 0.000e+00 0.000e+00 0.000e+00 0.000e+00
## Cumulative Proportion 1.000e+00 1.000e+00 1.000e+00 1.000e+00 1.000e+00
## PC930 PC931 PC932 PC933 PC934
## Standard deviation 1.661e-05 1.625e-05 1.575e-05 1.562e-05 1.543e-05
## Proportion of Variance 0.000e+00 0.000e+00 0.000e+00 0.000e+00 0.000e+00
## Cumulative Proportion 1.000e+00 1.000e+00 1.000e+00 1.000e+00 1.000e+00
## PC935 PC936 PC937 PC938 PC939
## Standard deviation 1.52e-05 1.513e-05 1.455e-05 1.434e-05 1.402e-05
## Proportion of Variance 0.00e+00 0.000e+00 0.000e+00 0.000e+00 0.000e+00
## Cumulative Proportion 1.00e+00 1.000e+00 1.000e+00 1.000e+00 1.000e+00
## PC940 PC941 PC942 PC943 PC944
## Standard deviation 1.38e-05 1.356e-05 1.342e-05 1.308e-05 1.281e-05
## Proportion of Variance 0.00e+00 0.000e+00 0.000e+00 0.000e+00 0.000e+00
## Cumulative Proportion 1.00e+00 1.000e+00 1.000e+00 1.000e+00 1.000e+00
## PC945 PC946 PC947 PC948 PC949
## Standard deviation 1.269e-05 1.248e-05 1.241e-05 1.206e-05 1.167e-05
## Proportion of Variance 0.000e+00 0.000e+00 0.000e+00 0.000e+00 0.000e+00
## Cumulative Proportion 1.000e+00 1.000e+00 1.000e+00 1.000e+00 1.000e+00
## PC950 PC951 PC952 PC953 PC954
## Standard deviation 1.159e-05 1.15e-05 1.128e-05 1.119e-05 1.088e-05
## Proportion of Variance 0.000e+00 0.00e+00 0.000e+00 0.000e+00 0.000e+00
## Cumulative Proportion 1.000e+00 1.00e+00 1.000e+00 1.000e+00 1.000e+00
## PC955 PC956 PC957 PC958 PC959
## Standard deviation 1.083e-05 1.049e-05 1.04e-05 1.003e-05 9.888e-06
## Proportion of Variance 0.000e+00 0.000e+00 0.00e+00 0.000e+00 0.000e+00
## Cumulative Proportion 1.000e+00 1.000e+00 1.00e+00 1.000e+00 1.000e+00
## PC960 PC961 PC962 PC963 PC964
## Standard deviation 9.669e-06 9.556e-06 9.255e-06 9.152e-06 9.082e-06
## Proportion of Variance 0.000e+00 0.000e+00 0.000e+00 0.000e+00 0.000e+00
## Cumulative Proportion 1.000e+00 1.000e+00 1.000e+00 1.000e+00 1.000e+00
## PC965 PC966 PC967 PC968 PC969
## Standard deviation 8.836e-06 8.591e-06 8.422e-06 8.375e-06 8.131e-06
## Proportion of Variance 0.000e+00 0.000e+00 0.000e+00 0.000e+00 0.000e+00
## Cumulative Proportion 1.000e+00 1.000e+00 1.000e+00 1.000e+00 1.000e+00
## PC970 PC971 PC972 PC973 PC974
## Standard deviation 8.02e-06 8.016e-06 7.775e-06 7.744e-06 7.515e-06
## Proportion of Variance 0.00e+00 0.000e+00 0.000e+00 0.000e+00 0.000e+00
## Cumulative Proportion 1.00e+00 1.000e+00 1.000e+00 1.000e+00 1.000e+00
## PC975 PC976 PC977 PC978 PC979
## Standard deviation 7.328e-06 7.128e-06 6.957e-06 6.792e-06 6.651e-06
## Proportion of Variance 0.000e+00 0.000e+00 0.000e+00 0.000e+00 0.000e+00
## Cumulative Proportion 1.000e+00 1.000e+00 1.000e+00 1.000e+00 1.000e+00
## PC980 PC981 PC982 PC983 PC984
## Standard deviation 6.579e-06 6.34e-06 6.27e-06 6.124e-06 6.022e-06
## Proportion of Variance 0.000e+00 0.00e+00 0.00e+00 0.000e+00 0.000e+00
## Cumulative Proportion 1.000e+00 1.00e+00 1.00e+00 1.000e+00 1.000e+00
## PC985 PC986 PC987 PC988 PC989
## Standard deviation 5.902e-06 5.738e-06 5.683e-06 5.553e-06 5.354e-06
## Proportion of Variance 0.000e+00 0.000e+00 0.000e+00 0.000e+00 0.000e+00
## Cumulative Proportion 1.000e+00 1.000e+00 1.000e+00 1.000e+00 1.000e+00
## PC990 PC991 PC992 PC993 PC994
## Standard deviation 5.262e-06 5.233e-06 5.077e-06 4.932e-06 4.828e-06
## Proportion of Variance 0.000e+00 0.000e+00 0.000e+00 0.000e+00 0.000e+00
## Cumulative Proportion 1.000e+00 1.000e+00 1.000e+00 1.000e+00 1.000e+00
## PC995 PC996 PC997 PC998 PC999
## Standard deviation 4.698e-06 4.597e-06 4.431e-06 4.277e-06 4.263e-06
## Proportion of Variance 0.000e+00 0.000e+00 0.000e+00 0.000e+00 0.000e+00
## Cumulative Proportion 1.000e+00 1.000e+00 1.000e+00 1.000e+00 1.000e+00
## PC1000 PC1001 PC1002 PC1003 PC1004
## Standard deviation 4.035e-06 3.895e-06 3.799e-06 3.713e-06 3.663e-06
## Proportion of Variance 0.000e+00 0.000e+00 0.000e+00 0.000e+00 0.000e+00
## Cumulative Proportion 1.000e+00 1.000e+00 1.000e+00 1.000e+00 1.000e+00
## PC1005 PC1006 PC1007 PC1008 PC1009
## Standard deviation 3.516e-06 3.484e-06 3.454e-06 3.343e-06 3.174e-06
## Proportion of Variance 0.000e+00 0.000e+00 0.000e+00 0.000e+00 0.000e+00
## Cumulative Proportion 1.000e+00 1.000e+00 1.000e+00 1.000e+00 1.000e+00
## PC1010 PC1011 PC1012 PC1013 PC1014
## Standard deviation 3.172e-06 3.066e-06 3.025e-06 2.95e-06 2.815e-06
## Proportion of Variance 0.000e+00 0.000e+00 0.000e+00 0.00e+00 0.000e+00
## Cumulative Proportion 1.000e+00 1.000e+00 1.000e+00 1.00e+00 1.000e+00
## PC1015 PC1016 PC1017 PC1018 PC1019
## Standard deviation 2.762e-06 2.704e-06 2.632e-06 2.601e-06 2.538e-06
## Proportion of Variance 0.000e+00 0.000e+00 0.000e+00 0.000e+00 0.000e+00
## Cumulative Proportion 1.000e+00 1.000e+00 1.000e+00 1.000e+00 1.000e+00
## PC1020 PC1021 PC1022 PC1023 PC1024
## Standard deviation 2.392e-06 2.33e-06 2.224e-06 2.192e-06 2.168e-06
## Proportion of Variance 0.000e+00 0.00e+00 0.000e+00 0.000e+00 0.000e+00
## Cumulative Proportion 1.000e+00 1.00e+00 1.000e+00 1.000e+00 1.000e+00
## PC1025 PC1026 PC1027 PC1028 PC1029
## Standard deviation 2.108e-06 2.028e-06 1.989e-06 1.9e-06 1.834e-06
## Proportion of Variance 0.000e+00 0.000e+00 0.000e+00 0.0e+00 0.000e+00
## Cumulative Proportion 1.000e+00 1.000e+00 1.000e+00 1.0e+00 1.000e+00
## PC1030 PC1031 PC1032 PC1033 PC1034
## Standard deviation 1.765e-06 1.713e-06 1.67e-06 1.633e-06 1.518e-06
## Proportion of Variance 0.000e+00 0.000e+00 0.00e+00 0.000e+00 0.000e+00
## Cumulative Proportion 1.000e+00 1.000e+00 1.00e+00 1.000e+00 1.000e+00
## PC1035 PC1036 PC1037 PC1038 PC1039
## Standard deviation 1.465e-06 1.354e-06 1.297e-06 1.245e-06 1.223e-06
## Proportion of Variance 0.000e+00 0.000e+00 0.000e+00 0.000e+00 0.000e+00
## Cumulative Proportion 1.000e+00 1.000e+00 1.000e+00 1.000e+00 1.000e+00
## PC1040 PC1041 PC1042 PC1043 PC1044
## Standard deviation 1.169e-06 1.125e-06 1.089e-06 9.827e-07 9.383e-07
## Proportion of Variance 0.000e+00 0.000e+00 0.000e+00 0.000e+00 0.000e+00
## Cumulative Proportion 1.000e+00 1.000e+00 1.000e+00 1.000e+00 1.000e+00
## PC1045 PC1046 PC1047 PC1048 PC1049
## Standard deviation 9.299e-07 8.285e-07 7.881e-07 7.448e-07 7.03e-07
## Proportion of Variance 0.000e+00 0.000e+00 0.000e+00 0.000e+00 0.00e+00
## Cumulative Proportion 1.000e+00 1.000e+00 1.000e+00 1.000e+00 1.00e+00
## PC1050 PC1051 PC1052 PC1053 PC1054
## Standard deviation 6.729e-07 5.975e-07 5.585e-07 5.347e-07 5.187e-07
## Proportion of Variance 0.000e+00 0.000e+00 0.000e+00 0.000e+00 0.000e+00
## Cumulative Proportion 1.000e+00 1.000e+00 1.000e+00 1.000e+00 1.000e+00
## PC1055 PC1056 PC1057 PC1058 PC1059
## Standard deviation 5.042e-07 4.674e-07 4.548e-07 4.353e-07 4.305e-07
## Proportion of Variance 0.000e+00 0.000e+00 0.000e+00 0.000e+00 0.000e+00
## Cumulative Proportion 1.000e+00 1.000e+00 1.000e+00 1.000e+00 1.000e+00
## PC1060 PC1061 PC1062 PC1063 PC1064
## Standard deviation 4.103e-07 3.774e-07 3.673e-07 3.432e-07 3.33e-07
## Proportion of Variance 0.000e+00 0.000e+00 0.000e+00 0.000e+00 0.00e+00
## Cumulative Proportion 1.000e+00 1.000e+00 1.000e+00 1.000e+00 1.00e+00
## PC1065 PC1066 PC1067 PC1068 PC1069
## Standard deviation 3.244e-07 3.062e-07 2.898e-07 2.8e-07 2.747e-07
## Proportion of Variance 0.000e+00 0.000e+00 0.000e+00 0.0e+00 0.000e+00
## Cumulative Proportion 1.000e+00 1.000e+00 1.000e+00 1.0e+00 1.000e+00
## PC1070 PC1071 PC1072 PC1073 PC1074
## Standard deviation 2.669e-07 2.599e-07 2.476e-07 2.384e-07 2.32e-07
## Proportion of Variance 0.000e+00 0.000e+00 0.000e+00 0.000e+00 0.00e+00
## Cumulative Proportion 1.000e+00 1.000e+00 1.000e+00 1.000e+00 1.00e+00
## PC1075 PC1076 PC1077 PC1078 PC1079
## Standard deviation 2.205e-07 2.145e-07 2.134e-07 2.075e-07 2.057e-07
## Proportion of Variance 0.000e+00 0.000e+00 0.000e+00 0.000e+00 0.000e+00
## Cumulative Proportion 1.000e+00 1.000e+00 1.000e+00 1.000e+00 1.000e+00
## PC1080 PC1081 PC1082 PC1083 PC1084
## Standard deviation 1.978e-07 1.925e-07 1.906e-07 1.837e-07 1.783e-07
## Proportion of Variance 0.000e+00 0.000e+00 0.000e+00 0.000e+00 0.000e+00
## Cumulative Proportion 1.000e+00 1.000e+00 1.000e+00 1.000e+00 1.000e+00
## PC1085 PC1086 PC1087 PC1088 PC1089
## Standard deviation 1.737e-07 1.73e-07 1.697e-07 1.619e-07 1.568e-07
## Proportion of Variance 0.000e+00 0.00e+00 0.000e+00 0.000e+00 0.000e+00
## Cumulative Proportion 1.000e+00 1.00e+00 1.000e+00 1.000e+00 1.000e+00
## PC1090 PC1091 PC1092 PC1093 PC1094
## Standard deviation 1.525e-07 1.505e-07 1.488e-07 1.466e-07 1.446e-07
## Proportion of Variance 0.000e+00 0.000e+00 0.000e+00 0.000e+00 0.000e+00
## Cumulative Proportion 1.000e+00 1.000e+00 1.000e+00 1.000e+00 1.000e+00
## PC1095 PC1096 PC1097 PC1098 PC1099
## Standard deviation 1.418e-07 1.408e-07 1.38e-07 1.368e-07 1.295e-07
## Proportion of Variance 0.000e+00 0.000e+00 0.00e+00 0.000e+00 0.000e+00
## Cumulative Proportion 1.000e+00 1.000e+00 1.00e+00 1.000e+00 1.000e+00
## PC1100 PC1101 PC1102 PC1103 PC1104
## Standard deviation 1.277e-07 1.255e-07 1.247e-07 1.222e-07 1.212e-07
## Proportion of Variance 0.000e+00 0.000e+00 0.000e+00 0.000e+00 0.000e+00
## Cumulative Proportion 1.000e+00 1.000e+00 1.000e+00 1.000e+00 1.000e+00
## PC1105 PC1106 PC1107 PC1108 PC1109
## Standard deviation 1.187e-07 1.177e-07 1.142e-07 1.127e-07 1.097e-07
## Proportion of Variance 0.000e+00 0.000e+00 0.000e+00 0.000e+00 0.000e+00
## Cumulative Proportion 1.000e+00 1.000e+00 1.000e+00 1.000e+00 1.000e+00
## PC1110 PC1111 PC1112 PC1113 PC1114
## Standard deviation 1.079e-07 1.06e-07 1.039e-07 1.027e-07 1.004e-07
## Proportion of Variance 0.000e+00 0.00e+00 0.000e+00 0.000e+00 0.000e+00
## Cumulative Proportion 1.000e+00 1.00e+00 1.000e+00 1.000e+00 1.000e+00
## PC1115 PC1116 PC1117 PC1118 PC1119
## Standard deviation 9.865e-08 9.672e-08 9.546e-08 9.4e-08 9.146e-08
## Proportion of Variance 0.000e+00 0.000e+00 0.000e+00 0.0e+00 0.000e+00
## Cumulative Proportion 1.000e+00 1.000e+00 1.000e+00 1.0e+00 1.000e+00
## PC1120 PC1121 PC1122 PC1123 PC1124
## Standard deviation 9.015e-08 8.932e-08 8.794e-08 8.588e-08 8.435e-08
## Proportion of Variance 0.000e+00 0.000e+00 0.000e+00 0.000e+00 0.000e+00
## Cumulative Proportion 1.000e+00 1.000e+00 1.000e+00 1.000e+00 1.000e+00
## PC1125 PC1126 PC1127 PC1128 PC1129
## Standard deviation 8.308e-08 8.091e-08 7.976e-08 7.879e-08 7.79e-08
## Proportion of Variance 0.000e+00 0.000e+00 0.000e+00 0.000e+00 0.00e+00
## Cumulative Proportion 1.000e+00 1.000e+00 1.000e+00 1.000e+00 1.00e+00
## PC1130 PC1131 PC1132 PC1133 PC1134
## Standard deviation 7.551e-08 7.423e-08 7.326e-08 7.221e-08 6.952e-08
## Proportion of Variance 0.000e+00 0.000e+00 0.000e+00 0.000e+00 0.000e+00
## Cumulative Proportion 1.000e+00 1.000e+00 1.000e+00 1.000e+00 1.000e+00
## PC1135 PC1136 PC1137 PC1138 PC1139
## Standard deviation 6.823e-08 6.706e-08 6.493e-08 6.393e-08 6.242e-08
## Proportion of Variance 0.000e+00 0.000e+00 0.000e+00 0.000e+00 0.000e+00
## Cumulative Proportion 1.000e+00 1.000e+00 1.000e+00 1.000e+00 1.000e+00
## PC1140 PC1141 PC1142 PC1143 PC1144
## Standard deviation 6.119e-08 6.08e-08 6.007e-08 5.798e-08 5.514e-08
## Proportion of Variance 0.000e+00 0.00e+00 0.000e+00 0.000e+00 0.000e+00
## Cumulative Proportion 1.000e+00 1.00e+00 1.000e+00 1.000e+00 1.000e+00
## PC1145 PC1146 PC1147 PC1148 PC1149
## Standard deviation 5.283e-08 5.126e-08 4.977e-08 4.857e-08 4.459e-08
## Proportion of Variance 0.000e+00 0.000e+00 0.000e+00 0.000e+00 0.000e+00
## Cumulative Proportion 1.000e+00 1.000e+00 1.000e+00 1.000e+00 1.000e+00
## PC1150 PC1151 PC1152 PC1153 PC1154
## Standard deviation 4.412e-08 4.268e-08 4.14e-08 3.956e-08 3.618e-08
## Proportion of Variance 0.000e+00 0.000e+00 0.00e+00 0.000e+00 0.000e+00
## Cumulative Proportion 1.000e+00 1.000e+00 1.00e+00 1.000e+00 1.000e+00

Robust and centralized pca (3D and 2D)

pca_scoresplot2D(ir.management.ds,ir.pca, "Profundidade",labels=F,pallette=2, ellipses = TRUE)


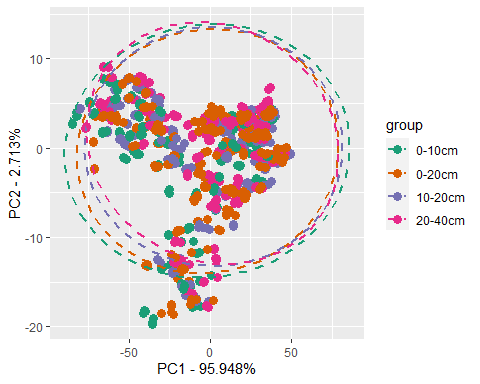


pca_scoresplot2D(ir.management.ds,ir.pca, "Manejo",labels=F,pallette=2, ellipses = T)


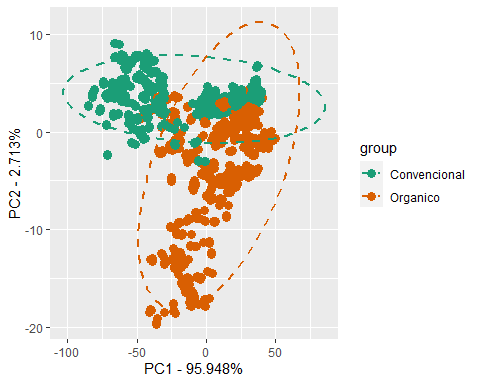


pca_scoresplot2D(ir.management.ds,ir.pca, "Especie",labels=F,pallette=2, ellipses = T)


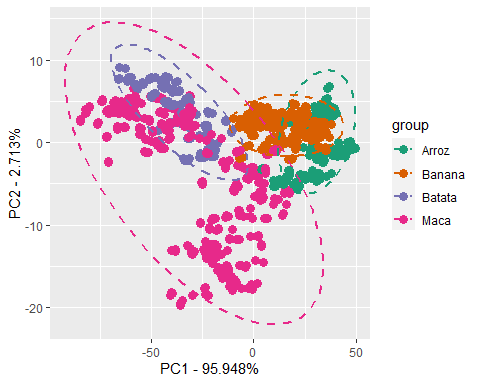


**Hierarchical Cluster Analysis**

ir.hc = clustering(ir.management.ds, method = "hc", distance = "euclidean")
dendrogram_plot(ir.management.ds, ir.hc, "Profundidade")


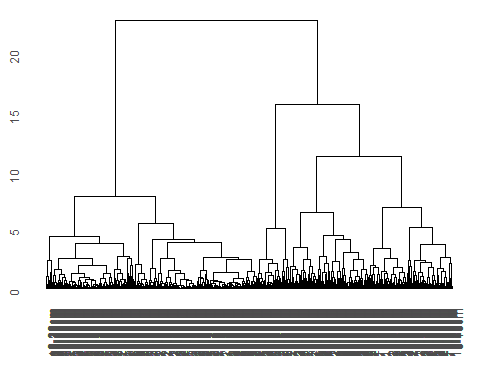


dendrogram_plot_col(ir.management.ds, ir.hc, "Profundidade")


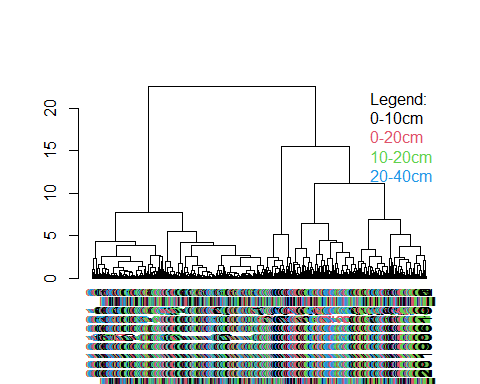


ir.management.ds.hc = clustering(ir.management.ds, method = "hc", distance = "euclidean")
dendrogram_plot(ir.management.ds, ir.management.ds.hc, "Manejo")


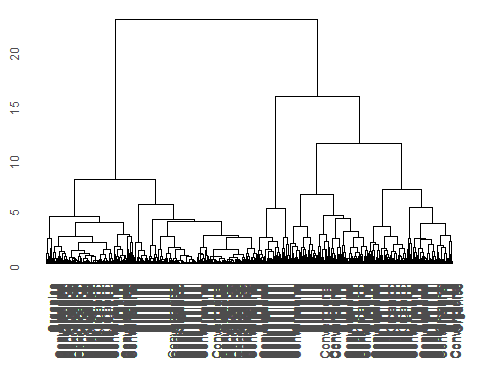


dendrogram_plot_col(ir.management.ds, ir.management.ds.hc, "Manejo")


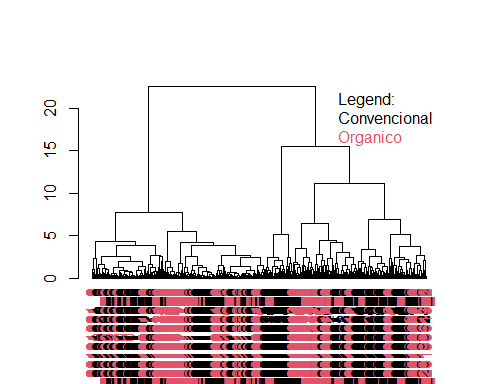


ir.management.ds.hc = clustering(ir.management.ds, method = "hc", distance = "euclidean")
dendrogram_plot(ir.management.ds, ir.management.ds.hc, "Especie")


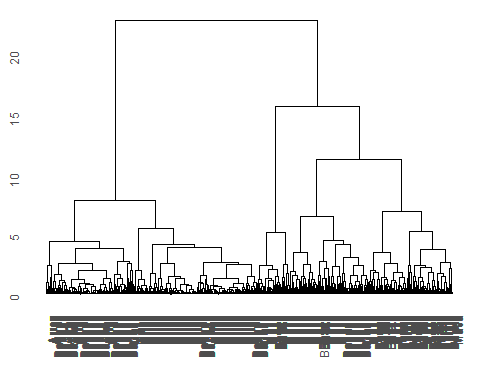


dendrogram_plot_col(ir.management.ds, ir.management.ds.hc, "Especie")


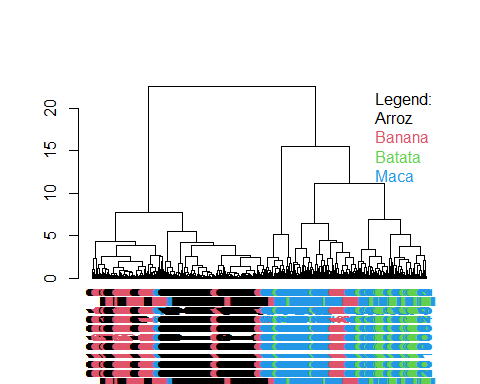


**Data Pre-Processing wiht baseline** **Smoothing and baseline correction**

ir.management.ds.smooth = smoothing_interpolation(ir.management.ds, method = "bin", reducing.factor = 5)

## Warning in hyperSpec::spc.bin(hyper.object, reducing.factor, na.rm = TRUE): Last
## data point averages only 4 points.

plot_spectra(ir.management.ds.smooth, "Profundidade", cex = 0.6)


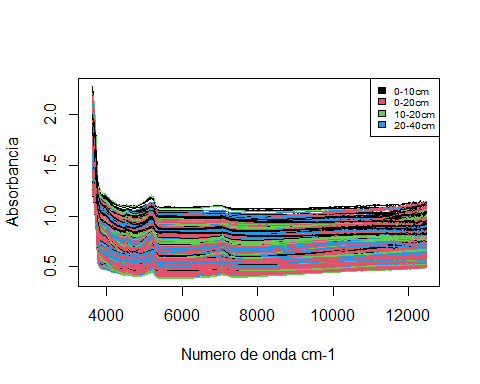


plot_spectra(ir.management.ds.smooth, "Manejo", cex = 0.6)


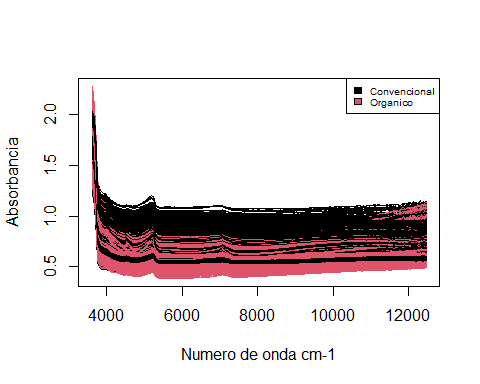


plot_spectra(ir.management.ds.smooth, "Especie", cex = 0.6)


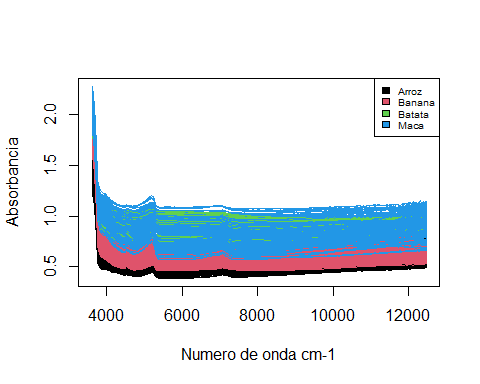


ir.management.ds.bg = data_correction(ir.management.ds.smooth,"background")
ir.management.ds.offset = data_correction(ir.management.ds.bg, "offset")
ir.management.ds.baseline = data_correction(ir.management.ds.offset, "baseline")
plot_spectra(ir.management.ds.baseline, "Profundidade", legend.place = "topright", cex = 0.76)


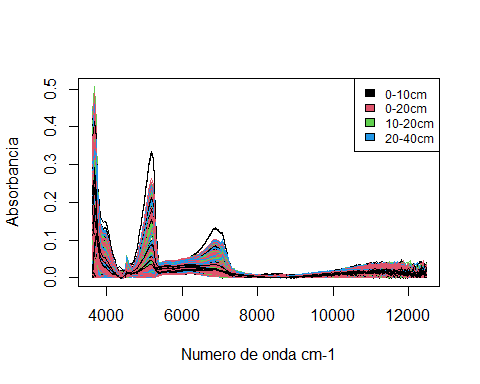


plot_spectra(ir.management.ds.baseline, "Manejo", legend.place = "topright", cex = 0.76)


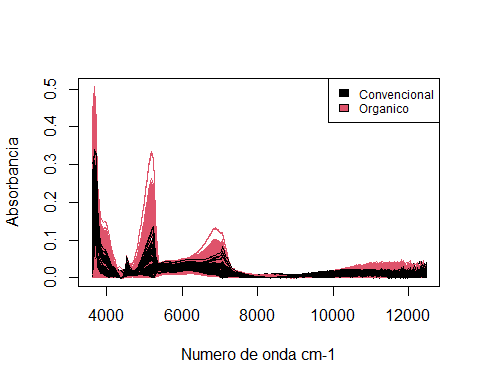


plot_spectra(ir.management.ds.baseline, "Especie", legend.place = "topright", cex = 0.76)


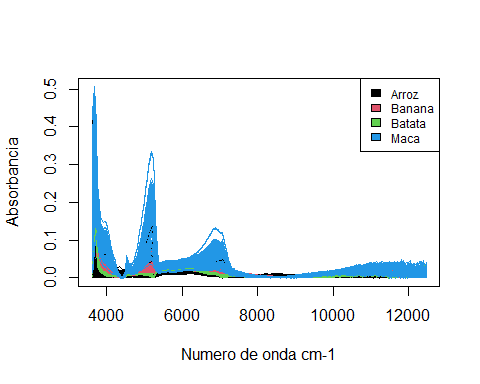


**UNIVARIATE ANALYSIS whit baseline**

ir.anova = aov_all_vars(ir.management.ds.baseline,"Profundidade")
ir.anova[1:10,]
ir.anova = aov_all_vars(ir.management.ds.baseline,"Manejo")
ir.anova[1:10,]
ir.anova = aov_all_vars(ir.management.ds.baseline,"Especie")
ir.anova[1:10,]

**T test whit baseline**

ir.convencional.organico = subset_samples_by_metadata_values(ir.management.ds.baseline, "Manejo", values = c("Convencional","Organico"))
ir.ttest = tTests_dataset(ir.convencional.organico, "Manejo")
ir.ttest[1:10,]

## p.value -log10 fdr
## 11548.3064634866 5.058085e-74 73.29601 6.314977e-72
## 11471.1634686904 5.467512e-74 73.26221 6.314977e-72
## 11509.7349660885 1.177852e-73 72.92891 9.069462e-72
## 11741.163950477 8.858206e-73 72.05265 5.115614e-71
## 3641.14949687771 1.211556e-71 70.91666 5.597390e-70
## 11586.8779608846 2.522251e-70 69.59821 9.710666e-69
## 3606.43514921943 3.270136e-70 69.48543 1.079145e-68
## 11702.5924530789 3.956551e-70 69.40268 1.142454e-68
## 11162.5914895056 4.395379e-68 67.35700 1.128147e-66
## 11625.4494582827 2.013160e-67 66.69612 4.650399e-66

plot_ttests(ir.convencional.organico, ir.ttest, tt.threshold = 0.05)


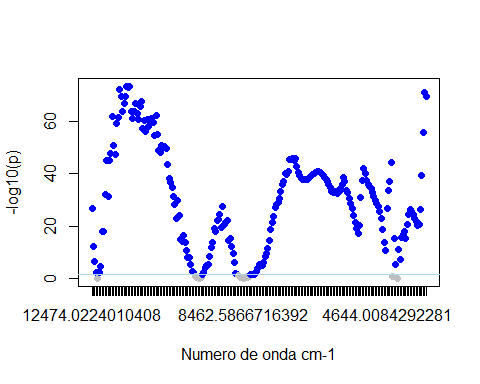


ir.convencional.organico = subset_samples_by_metadata_values(ir.management.ds.baseline, "Manejo", values = c("Convencional","Organico"))

ir.banana.batata = subset_samples_by_metadata_values(ir.management.ds.baseline, "Especie", values = c("Banana","Batata"))
ir.ttest = tTests_dataset(ir.banana.batata, "Especie")
ir.ttest[1:10,]

## p.value -log10 fdr
## 8886.87314301821 4.559327e-105 104.34110 1.053204e-102
## 8925.4446404163 1.121780e-94 93.95009 1.295656e-92
## 9079.73063000867 7.623122e-93 92.11787 5.869804e-91
## 9002.58763521249 1.018392e-90 89.99208 5.881214e-89
## 9118.30212740677 3.953935e-85 84.40297 1.826718e-83
## 8964.0161378144 2.177945e-84 83.66195 8.385087e-83
## 9156.87362480486 5.892751e-83 82.22968 1.944608e-81
## 9234.01661960104 1.651335e-80 79.78216 4.768229e-79
## 8809.73014822203 8.900109e-80 79.05060 2.284361e-78
## 9041.15913261058 1.196080e-78 77.92224 2.762944e-77

plot_ttests(ir.banana.batata, ir.ttest, tt.threshold = 0.05)


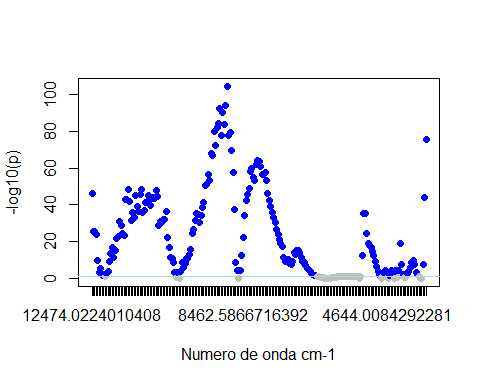


ir.banana.batata = subset_samples_by_metadata_values(ir.management.ds.baseline, "Especie", values = c("Banana","Batata"))

ir.maca.batata = subset_samples_by_metadata_values(ir.management.ds.baseline, "Especie", values = c("Maca","Batata"))
ir.ttest = tTests_dataset(ir.maca.batata, "Especie")
ir.ttest[1:10,]

## p.value -log10 fdr
## 3949.72147606244 1.211253e-81 80.91677 2.797995e-79
## 3911.14997866435 1.331763e-80 79.87557 1.538186e-78
## 3988.29297346054 3.804816e-80 79.41967 2.929708e-78
## 3795.43548647008 2.233720e-79 78.65097 1.111968e-77
## 3834.00698386817 2.406857e-79 78.61855 1.111968e-77
## 3872.57848126626 8.464697e-79 78.07239 3.258908e-77
## 4026.86447085863 1.973043e-77 76.70486 6.511040e-76
## 4065.43596825672 5.479367e-76 75.26127 1.582167e-74
## 4142.57896305291 2.733832e-75 74.56323 7.016834e-74
## 4104.00746565481 3.372766e-75 74.47201 7.791090e-74

plot_ttests(ir.banana.batata, ir.ttest, tt.threshold = 0.05)


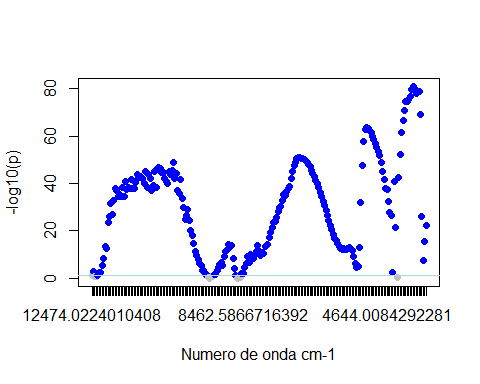


ir.maca.batata = subset_samples_by_metadata_values(ir.management.ds.baseline, "Especie", values = c("Maca","Batata"))

ir.banana.arroz = subset_samples_by_metadata_values(ir.management.ds.baseline, "Especie", values = c("Banana","Batata"))
ir.ttest = tTests_dataset(ir.banana.arroz, "Especie")
ir.ttest[1:10,]

## p.value -log10 fdr
## 8886.87314301821 4.559327e-105 104.34110 1.053204e-102
## 8925.4446404163 1.121780e-94 93.95009 1.295656e-92
## 9079.73063000867 7.623122e-93 92.11787 5.869804e-91
## 9002.58763521249 1.018392e-90 89.99208 5.881214e-89
## 9118.30212740677 3.953935e-85 84.40297 1.826718e-83
## 8964.0161378144 2.177945e-84 83.66195 8.385087e-83
## 9156.87362480486 5.892751e-83 82.22968 1.944608e-81
## 9234.01661960104 1.651335e-80 79.78216 4.768229e-79
## 8809.73014822203 8.900109e-80 79.05060 2.284361e-78
## 9041.15913261058 1.196080e-78 77.92224 2.762944e-77

plot_ttests(ir.banana.arroz, ir.ttest, tt.threshold = 0.05)


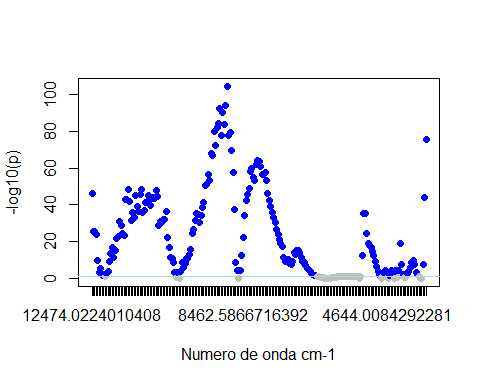


ir.banana.arroz = subset_samples_by_metadata_values(ir.management.ds.baseline, "Especie", values = c("Banana","Batata"))

**Principal Components Analysis whit baseline** Importance of components: Proportion of Variance explained in each component

ir.pca = pca_analysis_dataset(ir.management.ds.baseline)
summary(ir.pca, num.pcs = 10)

## Warning: In summary.prcomp(ir.pca, num.pcs = 10) :
## extra argument 'num.pcs' will be disregarded

## Importance of components:
## PC1 PC2 PC3 PC4 PC5 PC6 PC7
## Standard deviation 12.0170 5.4936 4.9048 3.20489 1.94492 1.37188 1.17902
## Proportion of Variance 0.6251 0.1306 0.1041 0.04446 0.01638 0.00815 0.00602
## Cumulative Proportion 0.6251 0.7558 0.8599 0.90440 0.92077 0.92892 0.93494
## PC8 PC9 PC10 PC11 PC12 PC13 PC14
## Standard deviation 1.01609 0.93789 0.8864 0.86230 0.80969 0.76621 0.75163
## Proportion of Variance 0.00447 0.00381 0.0034 0.00322 0.00284 0.00254 0.00245
## Cumulative Proportion 0.93941 0.94321 0.9466 0.94983 0.95267 0.95521 0.95766
## PC15 PC16 PC17 PC18 PC19 PC20 PC21
## Standard deviation 0.72428 0.68771 0.67424 0.65053 0.63329 0.61176 0.60002
## Proportion of Variance 0.00227 0.00205 0.00197 0.00183 0.00174 0.00162 0.00156
## Cumulative Proportion 0.95993 0.96198 0.96395 0.96578 0.96751 0.96913 0.97069
## PC22 PC23 PC24 PC25 PC26 PC27 PC28
## Standard deviation 0.5896 0.51861 0.5037 0.49444 0.47245 0.44525 0.42597
## Proportion of Variance 0.0015 0.00116 0.0011 0.00106 0.00097 0.00086 0.00079
## Cumulative Proportion 0.9722 0.97336 0.9745 0.97552 0.97649 0.97734 0.97813
## PC29 PC30 PC31 PC32 PC33 PC34 PC35
## Standard deviation 0.4017 0.38399 0.37992 0.35745 0.34960 0.3410 0.33176
## Proportion of Variance 0.0007 0.00064 0.00062 0.00055 0.00053 0.0005 0.00048
## Cumulative Proportion 0.9788 0.97947 0.98009 0.98064 0.98117 0.9817 0.98215
## PC36 PC37 PC38 PC39 PC40 PC41 PC42
## Standard deviation 0.32410 0.31647 0.3036 0.3025 0.29992 0.29623 0.28710
## Proportion of Variance 0.00045 0.00043 0.0004 0.0004 0.00039 0.00038 0.00036
## Cumulative Proportion 0.98261 0.98304 0.9834 0.9838 0.98423 0.98461 0.98496
## PC43 PC44 PC45 PC46 PC47 PC48 PC49
## Standard deviation 0.28562 0.28153 0.27951 0.27760 0.26914 0.26786 0.2642
## Proportion of Variance 0.00035 0.00034 0.00034 0.00033 0.00031 0.00031 0.0003
## Cumulative Proportion 0.98532 0.98566 0.98600 0.98633 0.98664 0.98695 0.9873
## PC50 PC51 PC52 PC53 PC54 PC55 PC56
## Standard deviation 0.2620 0.26005 0.25777 0.25601 0.25172 0.24926 0.24317
## Proportion of Variance 0.0003 0.00029 0.00029 0.00028 0.00027 0.00027 0.00026
## Cumulative Proportion 0.9876 0.98785 0.98813 0.98842 0.98869 0.98896 0.98922
## PC57 PC58 PC59 PC60 PC61 PC62 PC63
## Standard deviation 0.24133 0.23914 0.23614 0.23484 0.23417 0.23157 0.22803
## Proportion of Variance 0.00025 0.00025 0.00024 0.00024 0.00024 0.00023 0.00023
## Cumulative Proportion 0.98947 0.98972 0.98996 0.99020 0.99043 0.99067 0.99089
## PC64 PC65 PC66 PC67 PC68 PC69 PC70
## Standard deviation 0.22628 0.22480 0.22427 0.22081 0.21949 0.21762 0.2158
## Proportion of Variance 0.00022 0.00022 0.00022 0.00021 0.00021 0.00021 0.0002
## Cumulative Proportion 0.99111 0.99133 0.99155 0.99176 0.99197 0.99217 0.9924
## PC71 PC72 PC73 PC74 PC75 PC76 PC77
## Standard deviation 0.2133 0.21202 0.21040 0.20913 0.20710 0.20554 0.20181
## Proportion of Variance 0.0002 0.00019 0.00019 0.00019 0.00019 0.00018 0.00018
## Cumulative Proportion 0.9926 0.99277 0.99296 0.99315 0.99333 0.99352 0.99369
## PC78 PC79 PC80 PC81 PC82 PC83 PC84
## Standard deviation 0.20130 0.19720 0.19635 0.19508 0.19375 0.19296 0.19131
## Proportion of Variance 0.00018 0.00017 0.00017 0.00016 0.00016 0.00016 0.00016
## Cumulative Proportion 0.99387 0.99404 0.99420 0.99437 0.99453 0.99469 0.99485
## PC85 PC86 PC87 PC88 PC89 PC90 PC91
## Standard deviation 0.18914 0.18682 0.18638 0.18512 0.18318 0.17950 0.17778
## Proportion of Variance 0.00015 0.00015 0.00015 0.00015 0.00015 0.00014 0.00014
## Cumulative Proportion 0.99501 0.99516 0.99531 0.99546 0.99560 0.99574 0.99588
## PC92 PC93 PC94 PC95 PC96 PC97 PC98
## Standard deviation 0.17723 0.17554 0.17409 0.17325 0.17083 0.16988 0.16704
## Proportion of Variance 0.00014 0.00013 0.00013 0.00013 0.00013 0.00012 0.00012
## Cumulative Proportion 0.99601 0.99615 0.99628 0.99641 0.99653 0.99666 0.99678
## PC99 PC100 PC101 PC102 PC103 PC104 PC105
## Standard deviation 0.16618 0.16495 0.16102 0.15951 0.15883 0.15711 0.1548
## Proportion of Variance 0.00012 0.00012 0.00011 0.00011 0.00011 0.00011 0.0001
## Cumulative Proportion 0.99690 0.99702 0.99713 0.99724 0.99735 0.99746 0.9976
## PC106 PC107 PC108 PC109 PC110 PC111 PC112
## Standard deviation 0.1542 0.1507 0.1503 0.1485 0.14726 0.14518 0.14490
## Proportion of Variance 0.0001 0.0001 0.0001 0.0001 0.00009 0.00009 0.00009
## Cumulative Proportion 0.9977 0.9978 0.9979 0.9980 0.99805 0.99814 0.99823
## PC113 PC114 PC115 PC116 PC117 PC118 PC119
## Standard deviation 0.14221 0.14040 0.14000 0.13647 0.13386 0.13202 0.13027
## Proportion of Variance 0.00009 0.00009 0.00008 0.00008 0.00008 0.00008 0.00007
## Cumulative Proportion 0.99832 0.99840 0.99849 0.99857 0.99865 0.99872 0.99879
## PC120 PC121 PC122 PC123 PC124 PC125 PC126
## Standard deviation 0.12906 0.12810 0.12721 0.12496 0.12119 0.11854 0.11384
## Proportion of Variance 0.00007 0.00007 0.00007 0.00007 0.00006 0.00006 0.00006
## Cumulative Proportion 0.99887 0.99894 0.99901 0.99908 0.99914 0.99920 0.99926
## PC127 PC128 PC129 PC130 PC131 PC132 PC133
## Standard deviation 0.11302 0.11158 0.10910 0.10579 0.10455 0.10130 0.10053
## Proportion of Variance 0.00006 0.00005 0.00005 0.00005 0.00005 0.00004 0.00004
## Cumulative Proportion 0.99931 0.99937 0.99942 0.99947 0.99951 0.99956 0.99960
## PC134 PC135 PC136 PC137 PC138 PC139 PC140
## Standard deviation 0.09555 0.09260 0.08871 0.08313 0.08000 0.07738 0.07621
## Proportion of Variance 0.00004 0.00004 0.00003 0.00003 0.00003 0.00003 0.00003
## Cumulative Proportion 0.99964 0.99968 0.99971 0.99974 0.99977 0.99979 0.99982
## PC141 PC142 PC143 PC144 PC145 PC146 PC147
## Standard deviation 0.07147 0.06681 0.06100 0.05821 0.05436 0.04808 0.04716
## Proportion of Variance 0.00002 0.00002 0.00002 0.00001 0.00001 0.00001 0.00001
## Cumulative Proportion 0.99984 0.99986 0.99988 0.99989 0.99991 0.99992 0.99992
## PC148 PC149 PC150 PC151 PC152 PC153 PC154
## Standard deviation 0.04525 0.04269 0.04051 0.03786 0.03316 0.03029 0.02834
## Proportion of Variance 0.00001 0.00001 0.00001 0.00001 0.00000 0.00000 0.00000
## Cumulative Proportion 0.99993 0.99994 0.99995 0.99995 0.99996 0.99996 0.99997
## PC155 PC156 PC157 PC158 PC159 PC160 PC161
## Standard deviation 0.026 0.02502 0.02338 0.02171 0.01953 0.01891 0.01753
## Proportion of Variance 0.000 0.00000 0.00000 0.00000 0.00000 0.00000 0.00000
## Cumulative Proportion 1.000 0.99997 0.99997 0.99998 0.99998 0.99998 0.99998
## PC162 PC163 PC164 PC165 PC166 PC167 PC168
## Standard deviation 0.01656 0.01469 0.01367 0.01345 0.013 0.01217 0.01131
## Proportion of Variance 0.00000 0.00000 0.00000 0.00000 0.000 0.00000 0.00000
## Cumulative Proportion 0.99998 0.99998 0.99998 0.99999 1.000 0.99999 0.99999
## PC169 PC170 PC171 PC172 PC173 PC174
## Standard deviation 0.01088 0.01061 0.01049 0.01019 0.009964 0.00985
## Proportion of Variance 0.00000 0.00000 0.00000 0.00000 0.000000 0.00000
## Cumulative Proportion 0.99999 0.99999 0.99999 0.99999 0.999990 0.99999
## PC175 PC176 PC177 PC178 PC179 PC180
## Standard deviation 0.009694 0.009498 0.009352 0.009306 0.009112 0.00898
## Proportion of Variance 0.000000 0.000000 0.000000 0.000000 0.000000 0.00000
## Cumulative Proportion 0.999990 0.999990 0.999990 0.999990 0.999990 0.99999
## PC181 PC182 PC183 PC184 PC185 PC186
## Standard deviation 0.008802 0.008614 0.008564 0.008426 0.008241 0.008215
## Proportion of Variance 0.000000 0.000000 0.000000 0.000000 0.000000 0.000000
## Cumulative Proportion 0.999990 0.999990 0.999990 0.999990 0.999990 0.999990
## PC187 PC188 PC189 PC190 PC191 PC192
## Standard deviation 0.00807 0.007925 0.007708 0.007641 0.007622 0.007526
## Proportion of Variance 0.00000 0.000000 0.000000 0.000000 0.000000 0.000000
## Cumulative Proportion 0.99999 0.999990 0.999990 1.000000 1.000000 1.000000
## PC193 PC194 PC195 PC196 PC197 PC198
## Standard deviation 0.007441 0.007312 0.007209 0.00702 0.00687 0.006801
## Proportion of Variance 0.000000 0.000000 0.000000 0.00000 0.00000 0.000000
## Cumulative Proportion 1.000000 1.000000 1.000000 1.00000 1.00000 1.000000
## PC199 PC200 PC201 PC202 PC203 PC204
## Standard deviation 0.006587 0.006512 0.006451 0.006246 0.006189 0.006089
## Proportion of Variance 0.000000 0.000000 0.000000 0.000000 0.000000 0.000000
## Cumulative Proportion 1.000000 1.000000 1.000000 1.000000 1.000000 1.000000
## PC205 PC206 PC207 PC208 PC209 PC210
## Standard deviation 0.005924 0.005853 0.005616 0.005561 0.005418 0.005328
## Proportion of Variance 0.000000 0.000000 0.000000 0.000000 0.000000 0.000000
## Cumulative Proportion 1.000000 1.000000 1.000000 1.000000 1.000000 1.000000
## PC211 PC212 PC213 PC214 PC215 PC216
## Standard deviation 0.005248 0.005085 0.004982 0.004931 0.004767 0.004622
## Proportion of Variance 0.000000 0.000000 0.000000 0.000000 0.000000 0.000000
## Cumulative Proportion 1.000000 1.000000 1.000000 1.000000 1.000000 1.000000
## PC217 PC218 PC219 PC220 PC221 PC222
## Standard deviation 0.004497 0.004312 0.004121 0.00399 0.003597 0.003152
## Proportion of Variance 0.000000 0.000000 0.000000 0.00000 0.000000 0.000000
## Cumulative Proportion 1.000000 1.000000 1.000000 1.00000 1.000000 1.000000
## PC223 PC224 PC225 PC226 PC227 PC228
## Standard deviation 0.003003 0.002857 0.00264 0.002424 0.002023 0.001625
## Proportion of Variance 0.000000 0.000000 0.00000 0.000000 0.000000 0.000000
## Cumulative Proportion 1.000000 1.000000 1.00000 1.000000 1.000000 1.000000
## PC229 PC230 PC231
## Standard deviation 0.00146 0.001295 0.001087
## Proportion of Variance 0.00000 0.000000 0.000000
## Cumulative Proportion 1.00000 1.000000 1.000000

Robust and centralized pca (3D and 2D)

pca_scoresplot2D(ir.management.ds.baseline,ir.pca, "Profundidade",labels=F,pallette=2, ellipses = T)


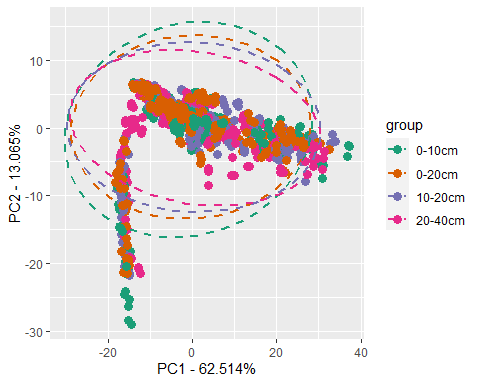


pca_scoresplot2D(ir.management.ds.baseline,ir.pca, "Manejo",labels=F,pallette=2, ellipses = T)


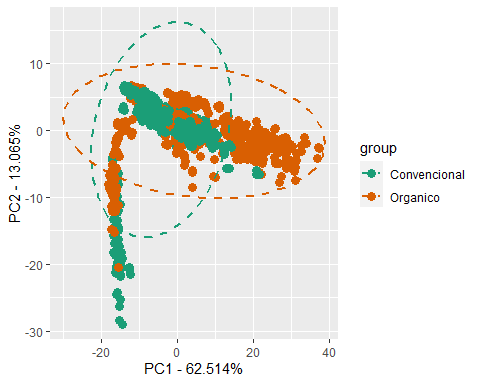


pca_scoresplot2D(ir.management.ds.baseline,ir.pca, "Especie",labels=F,pallette=2, ellipses = T)


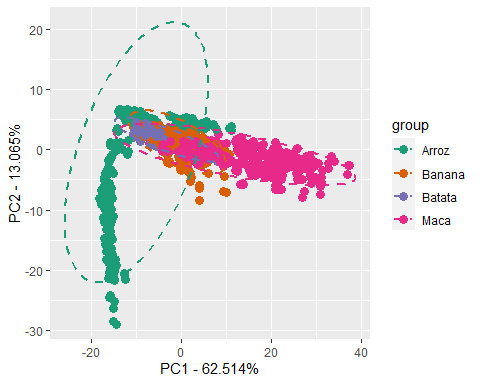


**Hierarchical Cluster Analysis whit baseline**

ir.hc = clustering(ir.management.ds.baseline, method = "hc", distance = "euclidean")
dendrogram_plot(ir.management.ds.baseline, ir.hc, "Profundidade")


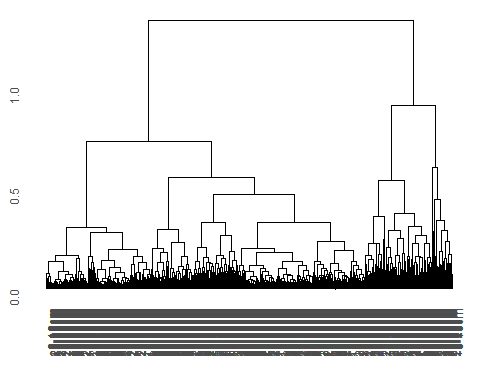


dendrogram_plot_col(ir.management.ds.baseline, ir.hc, "Profundidade")


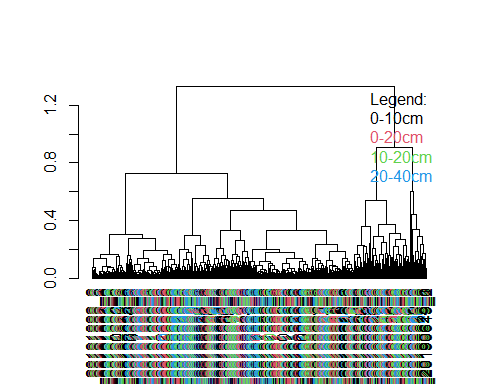


ir.management.ds.baseline.hc = clustering(ir.management.ds.baseline, method = "hc", distance = "euclidean")
dendrogram_plot(ir.management.ds.baseline, ir.management.ds.baseline.hc, "Manejo")


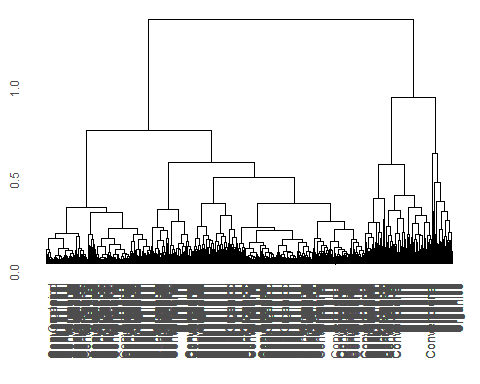


dendrogram_plot_col(ir.management.ds.baseline, ir.management.ds.baseline.hc, "Manejo")


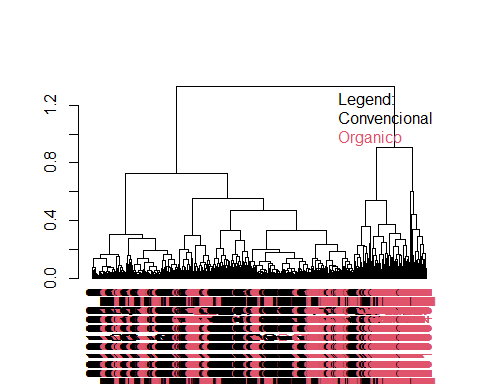


ir.management.ds.baseline.hc = clustering(ir.management.ds.baseline, method = "hc", distance = "euclidean")
dendrogram_plot(ir.management.ds.baseline, ir.management.ds.baseline.hc, "Especie")


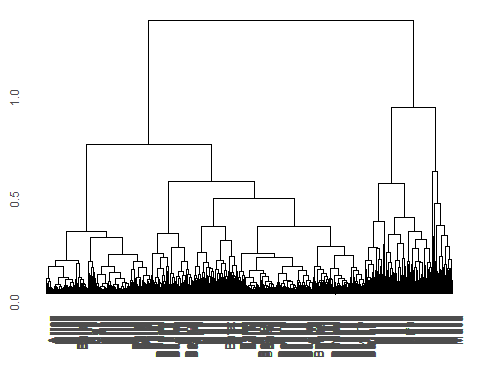


dendrogram_plot_col(ir.management.ds.baseline, ir.management.ds.baseline.hc, "Especie")


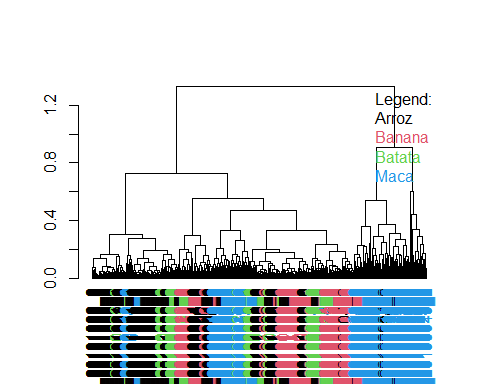


**Management FINGERPRINT REGION (8000.0 4000.0 cm-1)** Importance of components: Proportion of Variance explained in each component

ir.corte = subset_x_values_by_interval(ir.management.ds, min.value = 4000, max.value = 8000)

**Plotting the Spectra**

plot_spectra_simple(ir.corte)


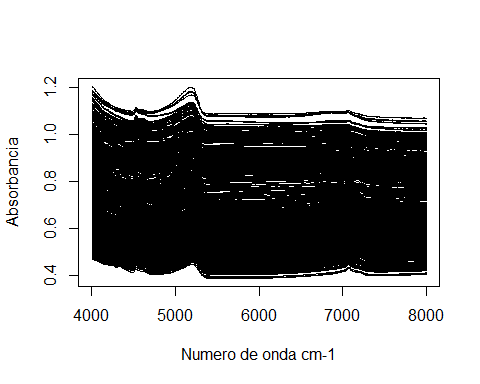


plot_spectra(ir.corte, "Profundidade", legend.place = "topright", cex = 0.76)


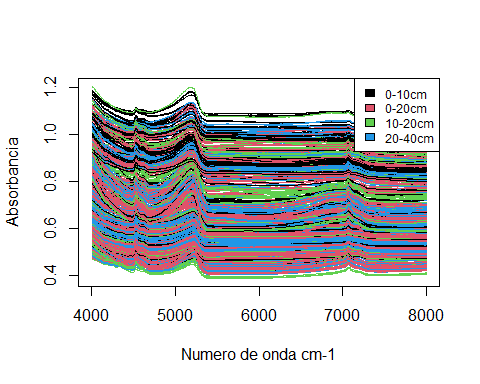


plot_spectra(ir.corte, "Manejo", legend.place = "topright", cex = 0.76)


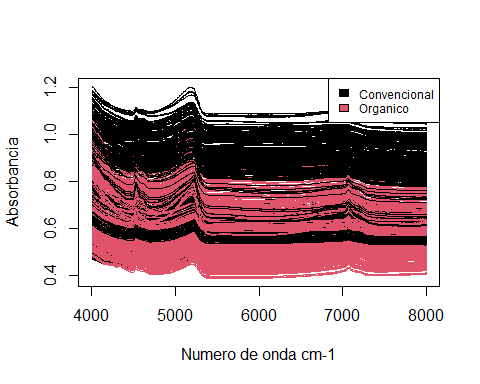


plot_spectra(ir.corte, "Especie", legend.place = "topright", cex = 0.76)


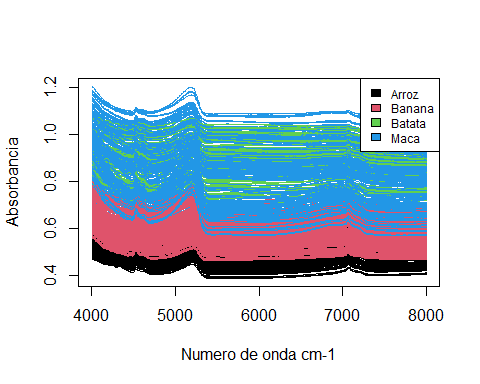


**Principal Components Analysis**

ir.corte.pca = pca_analysis_dataset(ir.corte)
summary(ir.corte.pca)

## Importance of components:
## PC1 PC2 PC3 PC4 PC5 PC6 PC7
## Standard deviation 22.6260 2.5166 0.7215 0.41698 0.14544 0.07063 0.06711
## Proportion of Variance 0.9864 0.0122 0.0010 0.00034 0.00004 0.00001 0.00001
## Cumulative Proportion 0.9864 0.9986 0.9996 0.99993 0.99997 0.99998 0.99999
## PC8 PC9 PC10 PC11 PC12 PC13 PC14
## Standard deviation 0.04878 0.03443 0.02894 0.02289 0.02175 0.0153 0.01248
## Proportion of Variance 0.00000 0.00000 0.00000 0.00000 0.00000 0.0000 0.00000
## Cumulative Proportion 0.99999 0.99999 1.00000 1.00000 1.00000 1.0000 1.00000
## PC15 PC16 PC17 PC18 PC19 PC20
## Standard deviation 0.009741 0.008413 0.006608 0.006514 0.005725 0.004192
## Proportion of Variance 0.000000 0.000000 0.000000 0.000000 0.000000 0.000000
## Cumulative Proportion 1.000000 1.000000 1.000000 1.000000 1.000000 1.000000
## PC21 PC22 PC23 PC24 PC25 PC26
## Standard deviation 0.003594 0.003341 0.003283 0.003241 0.003184 0.003075
## Proportion of Variance 0.000000 0.000000 0.000000 0.000000 0.000000 0.000000
## Cumulative Proportion 1.000000 1.000000 1.000000 1.000000 1.000000 1.000000
## PC27 PC28 PC29 PC30 PC31 PC32
## Standard deviation 0.003064 0.002986 0.002943 0.002854 0.002802 0.002709
## Proportion of Variance 0.000000 0.000000 0.000000 0.000000 0.000000 0.000000
## Cumulative Proportion 1.000000 1.000000 1.000000 1.000000 1.000000 1.000000
## PC33 PC34 PC35 PC36 PC37 PC38
## Standard deviation 0.002705 0.002655 0.002635 0.002586 0.002548 0.002518
## Proportion of Variance 0.000000 0.000000 0.000000 0.000000 0.000000 0.000000
## Cumulative Proportion 1.000000 1.000000 1.000000 1.000000 1.000000 1.000000
## PC39 PC40 PC41 PC42 PC43 PC44
## Standard deviation 0.00247 0.002432 0.002388 0.002369 0.002352 0.002277
## Proportion of Variance 0.00000 0.000000 0.000000 0.000000 0.000000 0.000000
## Cumulative Proportion 1.00000 1.000000 1.000000 1.000000 1.000000 1.000000
## PC45 PC46 PC47 PC48 PC49 PC50
## Standard deviation 0.002215 0.00219 0.002145 0.002122 0.002109 0.002073
## Proportion of Variance 0.000000 0.00000 0.000000 0.000000 0.000000 0.000000
## Cumulative Proportion 1.000000 1.00000 1.000000 1.000000 1.000000 1.000000
## PC51 PC52 PC53 PC54 PC55 PC56
## Standard deviation 0.002042 0.002007 0.001988 0.001966 0.00194 0.001915
## Proportion of Variance 0.000000 0.000000 0.000000 0.000000 0.00000 0.000000
## Cumulative Proportion 1.000000 1.000000 1.000000 1.000000 1.00000 1.000000
## PC57 PC58 PC59 PC60 PC61 PC62
## Standard deviation 0.001897 0.001867 0.001852 0.001831 0.001805 0.001779
## Proportion of Variance 0.000000 0.000000 0.000000 0.000000 0.000000 0.000000
## Cumulative Proportion 1.000000 1.000000 1.000000 1.000000 1.000000 1.000000
## PC63 PC64 PC65 PC66 PC67 PC68
## Standard deviation 0.001768 0.001736 0.001717 0.001714 0.001693 0.001674
## Proportion of Variance 0.000000 0.000000 0.000000 0.000000 0.000000 0.000000
## Cumulative Proportion 1.000000 1.000000 1.000000 1.000000 1.000000 1.000000
## PC69 PC70 PC71 PC72 PC73 PC74
## Standard deviation 0.001654 0.001636 0.00162 0.001607 0.001599 0.001576
## Proportion of Variance 0.000000 0.000000 0.00000 0.000000 0.000000 0.000000
## Cumulative Proportion 1.000000 1.000000 1.00000 1.000000 1.000000 1.000000
## PC75 PC76 PC77 PC78 PC79 PC80
## Standard deviation 0.00156 0.001547 0.001511 0.001503 0.001496 0.001466
## Proportion of Variance 0.00000 0.000000 0.000000 0.000000 0.000000 0.000000
## Cumulative Proportion 1.00000 1.000000 1.000000 1.000000 1.000000 1.000000
## PC81 PC82 PC83 PC84 PC85 PC86
## Standard deviation 0.001455 0.001447 0.001435 0.001414 0.001389 0.001376
## Proportion of Variance 0.000000 0.000000 0.000000 0.000000 0.000000 0.000000
## Cumulative Proportion 1.000000 1.000000 1.000000 1.000000 1.000000 1.000000
## PC87 PC88 PC89 PC90 PC91 PC92
## Standard deviation 0.00137 0.001345 0.001344 0.001333 0.001325 0.001318
## Proportion of Variance 0.00000 0.000000 0.000000 0.000000 0.000000 0.000000
## Cumulative Proportion 1.00000 1.000000 1.000000 1.000000 1.000000 1.000000
## PC93 PC94 PC95 PC96 PC97 PC98
## Standard deviation 0.001304 0.001285 0.001269 0.001257 0.001242 0.001234
## Proportion of Variance 0.000000 0.000000 0.000000 0.000000 0.000000 0.000000
## Cumulative Proportion 1.000000 1.000000 1.000000 1.000000 1.000000 1.000000
## PC99 PC100 PC101 PC102 PC103 PC104
## Standard deviation 0.001213 0.001207 0.001201 0.001194 0.001183 0.001175
## Proportion of Variance 0.000000 0.000000 0.000000 0.000000 0.000000 0.000000
## Cumulative Proportion 1.000000 1.000000 1.000000 1.000000 1.000000 1.000000
## PC105 PC106 PC107 PC108 PC109 PC110
## Standard deviation 0.001169 0.001157 0.001144 0.001133 0.00112 0.001116
## Proportion of Variance 0.000000 0.000000 0.000000 0.000000 0.00000 0.000000
## Cumulative Proportion 1.000000 1.000000 1.000000 1.000000 1.00000 1.000000
## PC111 PC112 PC113 PC114 PC115 PC116
## Standard deviation 0.001108 0.0011 0.001092 0.001079 0.001075 0.001061
## Proportion of Variance 0.000000 0.0000 0.000000 0.000000 0.000000 0.000000
## Cumulative Proportion 1.000000 1.0000 1.000000 1.000000 1.000000 1.000000
## PC117 PC118 PC119 PC120 PC121 PC122
## Standard deviation 0.001057 0.001052 0.001034 0.001025 0.001021 0.001011
## Proportion of Variance 0.000000 0.000000 0.000000 0.000000 0.000000 0.000000
## Cumulative Proportion 1.000000 1.000000 1.000000 1.000000 1.000000 1.000000
## PC123 PC124 PC125 PC126 PC127
## Standard deviation 0.001009 0.001002 0.0009974 0.0009919 0.0009734
## Proportion of Variance 0.000000 0.000000 0.0000000 0.0000000 0.0000000
## Cumulative Proportion 1.000000 1.000000 1.0000000 1.0000000 1.0000000
## PC128 PC129 PC130 PC131 PC132
## Standard deviation 0.0009705 0.0009546 0.0009508 0.0009414 0.000932
## Proportion of Variance 0.0000000 0.0000000 0.0000000 0.0000000 0.000000
## Cumulative Proportion 1.0000000 1.0000000 1.0000000 1.0000000 1.000000
## PC133 PC134 PC135 PC136 PC137
## Standard deviation 0.0009262 0.0009144 0.0009077 0.0009029 0.0008938
## Proportion of Variance 0.0000000 0.0000000 0.0000000 0.0000000 0.0000000
## Cumulative Proportion 1.0000000 1.0000000 1.0000000 1.0000000 1.0000000
## PC138 PC139 PC140 PC141 PC142
## Standard deviation 0.0008858 0.0008752 0.0008683 0.0008674 0.0008564
## Proportion of Variance 0.0000000 0.0000000 0.0000000 0.0000000 0.0000000
## Cumulative Proportion 1.0000000 1.0000000 1.0000000 1.0000000 1.0000000
## PC143 PC144 PC145 PC146 PC147
## Standard deviation 0.0008532 0.0008498 0.0008421 0.0008352 0.0008264
## Proportion of Variance 0.0000000 0.0000000 0.0000000 0.0000000 0.0000000
## Cumulative Proportion 1.0000000 1.0000000 1.0000000 1.0000000 1.0000000
## PC148 PC149 PC150 PC151 PC152
## Standard deviation 0.0008201 0.0008104 0.0008075 0.0008017 0.0007924
## Proportion of Variance 0.0000000 0.0000000 0.0000000 0.0000000 0.0000000
## Cumulative Proportion 1.0000000 1.0000000 1.0000000 1.0000000 1.0000000
## PC153 PC154 PC155 PC156 PC157
## Standard deviation 0.0007863 0.0007836 0.000777 0.0007757 0.0007655
## Proportion of Variance 0.0000000 0.0000000 0.000000 0.0000000 0.0000000
## Cumulative Proportion 1.0000000 1.0000000 1.000000 1.0000000 1.0000000
## PC158 PC159 PC160 PC161 PC162
## Standard deviation 0.0007614 0.0007556 0.0007469 0.0007403 0.0007258
## Proportion of Variance 0.0000000 0.0000000 0.0000000 0.0000000 0.0000000
## Cumulative Proportion 1.0000000 1.0000000 1.0000000 1.0000000 1.0000000
## PC163 PC164 PC165 PC166 PC167
## Standard deviation 0.0007233 0.0007197 0.000712 0.0007052 0.0006987
## Proportion of Variance 0.0000000 0.0000000 0.000000 0.0000000 0.0000000
## Cumulative Proportion 1.0000000 1.0000000 1.000000 1.0000000 1.0000000
## PC168 PC169 PC170 PC171 PC172
## Standard deviation 0.0006967 0.0006917 0.0006832 0.0006751 0.0006702
## Proportion of Variance 0.0000000 0.0000000 0.0000000 0.0000000 0.0000000
## Cumulative Proportion 1.0000000 1.0000000 1.0000000 1.0000000 1.0000000
## PC173 PC174 PC175 PC176 PC177
## Standard deviation 0.0006634 0.0006601 0.0006579 0.0006533 0.0006522
## Proportion of Variance 0.0000000 0.0000000 0.0000000 0.0000000 0.0000000
## Cumulative Proportion 1.0000000 1.0000000 1.0000000 1.0000000 1.0000000
## PC178 PC179 PC180 PC181 PC182
## Standard deviation 0.0006431 0.000635 0.0006346 0.000628 0.0006227
## Proportion of Variance 0.0000000 0.000000 0.0000000 0.000000 0.0000000
## Cumulative Proportion 1.0000000 1.000000 1.0000000 1.000000 1.0000000
## PC183 PC184 PC185 PC186 PC187
## Standard deviation 0.0006178 0.0006164 0.0006075 0.0006022 0.0005964
## Proportion of Variance 0.0000000 0.0000000 0.0000000 0.0000000 0.0000000
## Cumulative Proportion 1.0000000 1.0000000 1.0000000 1.0000000 1.0000000
## PC188 PC189 PC190 PC191 PC192
## Standard deviation 0.0005889 0.0005825 0.0005745 0.0005719 0.0005691
## Proportion of Variance 0.0000000 0.0000000 0.0000000 0.0000000 0.0000000
## Cumulative Proportion 1.0000000 1.0000000 1.0000000 1.0000000 1.0000000
## PC193 PC194 PC195 PC196 PC197
## Standard deviation 0.0005586 0.0005535 0.0005499 0.0005464 0.0005421
## Proportion of Variance 0.0000000 0.0000000 0.0000000 0.0000000 0.0000000
## Cumulative Proportion 1.0000000 1.0000000 1.0000000 1.0000000 1.0000000
## PC198 PC199 PC200 PC201 PC202
## Standard deviation 0.0005363 0.0005299 0.0005275 0.0005242 0.0005127
## Proportion of Variance 0.0000000 0.0000000 0.0000000 0.0000000 0.0000000
## Cumulative Proportion 1.0000000 1.0000000 1.0000000 1.0000000 1.0000000
## PC203 PC204 PC205 PC206 PC207
## Standard deviation 0.0005117 0.0005063 0.0005045 0.0005003 0.0004957
## Proportion of Variance 0.0000000 0.0000000 0.0000000 0.0000000 0.0000000
## Cumulative Proportion 1.0000000 1.0000000 1.0000000 1.0000000 1.0000000
## PC208 PC209 PC210 PC211 PC212
## Standard deviation 0.0004928 0.000485 0.000479 0.0004733 0.0004686
## Proportion of Variance 0.0000000 0.000000 0.000000 0.0000000 0.0000000
## Cumulative Proportion 1.0000000 1.000000 1.000000 1.0000000 1.0000000
## PC213 PC214 PC215 PC216 PC217
## Standard deviation 0.0004648 0.0004612 0.000457 0.0004516 0.0004467
## Proportion of Variance 0.0000000 0.0000000 0.000000 0.0000000 0.0000000
## Cumulative Proportion 1.0000000 1.0000000 1.000000 1.0000000 1.0000000
## PC218 PC219 PC220 PC221 PC222
## Standard deviation 0.0004387 0.0004344 0.0004271 0.000424 0.0004217
## Proportion of Variance 0.0000000 0.0000000 0.0000000 0.000000 0.0000000
## Cumulative Proportion 1.0000000 1.0000000 1.0000000 1.000000 1.0000000
## PC223 PC224 PC225 PC226 PC227
## Standard deviation 0.0004162 0.0004115 0.0004071 0.0004051 0.0004018
## Proportion of Variance 0.0000000 0.0000000 0.0000000 0.0000000 0.0000000
## Cumulative Proportion 1.0000000 1.0000000 1.0000000 1.0000000 1.0000000
## PC228 PC229 PC230 PC231 PC232
## Standard deviation 0.0003989 0.0003903 0.0003876 0.000383 0.000379
## Proportion of Variance 0.0000000 0.0000000 0.0000000 0.000000 0.000000
## Cumulative Proportion 1.0000000 1.0000000 1.0000000 1.000000 1.000000
## PC233 PC234 PC235 PC236 PC237
## Standard deviation 0.0003742 0.0003684 0.0003639 0.0003624 0.0003587
## Proportion of Variance 0.0000000 0.0000000 0.0000000 0.0000000 0.0000000
## Cumulative Proportion 1.0000000 1.0000000 1.0000000 1.0000000 1.0000000
## PC238 PC239 PC240 PC241 PC242
## Standard deviation 0.0003521 0.0003493 0.0003442 0.0003438 0.0003349
## Proportion of Variance 0.0000000 0.0000000 0.0000000 0.0000000 0.0000000
## Cumulative Proportion 1.0000000 1.0000000 1.0000000 1.0000000 1.0000000
## PC243 PC244 PC245 PC246 PC247
## Standard deviation 0.0003333 0.0003293 0.0003262 0.0003234 0.0003177
## Proportion of Variance 0.0000000 0.0000000 0.0000000 0.0000000 0.0000000
## Cumulative Proportion 1.0000000 1.0000000 1.0000000 1.0000000 1.0000000
## PC248 PC249 PC250 PC251 PC252 PC253
## Standard deviation 0.0003127 0.0003088 0.0003075 0.0003043 3e-04 0.0002975
## Proportion of Variance 0.0000000 0.0000000 0.0000000 0.0000000 0e+00 0.0000000
## Cumulative Proportion 1.0000000 1.0000000 1.0000000 1.0000000 1e+00 1.0000000
## PC254 PC255 PC256 PC257 PC258
## Standard deviation 0.0002953 0.0002922 0.0002887 0.0002861 0.000281
## Proportion of Variance 0.0000000 0.0000000 0.0000000 0.0000000 0.000000
## Cumulative Proportion 1.0000000 1.0000000 1.0000000 1.0000000 1.000000
## PC259 PC260 PC261 PC262 PC263 PC264
## Standard deviation 0.000278 0.0002739 0.000272 0.0002638 0.0002614 0.00026
## Proportion of Variance 0.000000 0.0000000 0.000000 0.0000000 0.0000000 0.00000
## Cumulative Proportion 1.000000 1.0000000 1.000000 1.0000000 1.0000000 1.00000
## PC265 PC266 PC267 PC268 PC269
## Standard deviation 0.0002559 0.000251 0.0002494 0.0002466 0.0002432
## Proportion of Variance 0.0000000 0.000000 0.0000000 0.0000000 0.0000000
## Cumulative Proportion 1.0000000 1.000000 1.0000000 1.0000000 1.0000000
## PC270 PC271 PC272 PC273 PC274
## Standard deviation 0.0002395 0.0002364 0.0002336 0.0002306 0.0002285
## Proportion of Variance 0.0000000 0.0000000 0.0000000 0.0000000 0.0000000
## Cumulative Proportion 1.0000000 1.0000000 1.0000000 1.0000000 1.0000000
## PC275 PC276 PC277 PC278 PC279
## Standard deviation 0.0002268 0.0002219 0.00022 0.0002163 0.0002132
## Proportion of Variance 0.0000000 0.0000000 0.00000 0.0000000 0.0000000
## Cumulative Proportion 1.0000000 1.0000000 1.00000 1.0000000 1.0000000
## PC280 PC281 PC282 PC283 PC284
## Standard deviation 0.0002103 0.0002066 0.000204 0.0001992 0.0001984
## Proportion of Variance 0.0000000 0.0000000 0.000000 0.0000000 0.0000000
## Cumulative Proportion 1.0000000 1.0000000 1.000000 1.0000000 1.0000000
## PC285 PC286 PC287 PC288 PC289
## Standard deviation 0.000196 0.0001928 0.0001858 0.0001822 0.0001817
## Proportion of Variance 0.000000 0.0000000 0.0000000 0.0000000 0.0000000
## Cumulative Proportion 1.000000 1.0000000 1.0000000 1.0000000 1.0000000
## PC290 PC291 PC292 PC293 PC294
## Standard deviation 0.0001799 0.0001776 0.0001749 0.0001728 0.0001663
## Proportion of Variance 0.0000000 0.0000000 0.0000000 0.0000000 0.0000000
## Cumulative Proportion 1.0000000 1.0000000 1.0000000 1.0000000 1.0000000
## PC295 PC296 PC297 PC298 PC299
## Standard deviation 0.0001646 0.0001625 0.0001609 0.0001588 0.0001548
## Proportion of Variance 0.0000000 0.0000000 0.0000000 0.0000000 0.0000000
## Cumulative Proportion 1.0000000 1.0000000 1.0000000 1.0000000 1.0000000
## PC300 PC301 PC302 PC303 PC304
## Standard deviation 0.0001524 0.0001518 0.0001481 0.0001455 0.0001436
## Proportion of Variance 0.0000000 0.0000000 0.0000000 0.0000000 0.0000000
## Cumulative Proportion 1.0000000 1.0000000 1.0000000 1.0000000 1.0000000
## PC305 PC306 PC307 PC308 PC309
## Standard deviation 0.0001419 0.0001414 0.0001396 0.0001364 0.0001347
## Proportion of Variance 0.0000000 0.0000000 0.0000000 0.0000000 0.0000000
## Cumulative Proportion 1.0000000 1.0000000 1.0000000 1.0000000 1.0000000
## PC310 PC311 PC312 PC313 PC314
## Standard deviation 0.0001318 0.0001299 0.000129 0.0001265 0.0001251
## Proportion of Variance 0.0000000 0.0000000 0.000000 0.0000000 0.0000000
## Cumulative Proportion 1.0000000 1.0000000 1.000000 1.0000000 1.0000000
## PC315 PC316 PC317 PC318 PC319
## Standard deviation 0.0001226 0.0001206 0.0001158 0.000115 0.0001127
## Proportion of Variance 0.0000000 0.0000000 0.0000000 0.000000 0.0000000
## Cumulative Proportion 1.0000000 1.0000000 1.0000000 1.000000 1.0000000
## PC320 PC321 PC322 PC323 PC324
## Standard deviation 0.0001115 0.0001088 0.0001074 0.0001062 0.0001036
## Proportion of Variance 0.0000000 0.0000000 0.0000000 0.0000000 0.0000000
## Cumulative Proportion 1.0000000 1.0000000 1.0000000 1.0000000 1.0000000
## PC325 PC326 PC327 PC328 PC329
## Standard deviation 0.0001014 9.969e-05 9.824e-05 9.7e-05 9.595e-05
## Proportion of Variance 0.0000000 0.000e+00 0.000e+00 0.0e+00 0.000e+00
## Cumulative Proportion 1.0000000 1.000e+00 1.000e+00 1.0e+00 1.000e+00
## PC330 PC331 PC332 PC333 PC334
## Standard deviation 9.439e-05 9.266e-05 9.003e-05 8.804e-05 8.656e-05
## Proportion of Variance 0.000e+00 0.000e+00 0.000e+00 0.000e+00 0.000e+00
## Cumulative Proportion 1.000e+00 1.000e+00 1.000e+00 1.000e+00 1.000e+00
## PC335 PC336 PC337 PC338 PC339
## Standard deviation 8.43e-05 8.326e-05 8.133e-05 8.047e-05 7.935e-05
## Proportion of Variance 0.00e+00 0.000e+00 0.000e+00 0.000e+00 0.000e+00
## Cumulative Proportion 1.00e+00 1.000e+00 1.000e+00 1.000e+00 1.000e+00
## PC340 PC341 PC342 PC343 PC344
## Standard deviation 7.794e-05 7.497e-05 7.337e-05 7.134e-05 6.969e-05
## Proportion of Variance 0.000e+00 0.000e+00 0.000e+00 0.000e+00 0.000e+00
## Cumulative Proportion 1.000e+00 1.000e+00 1.000e+00 1.000e+00 1.000e+00
## PC345 PC346 PC347 PC348 PC349
## Standard deviation 6.941e-05 6.744e-05 6.521e-05 6.427e-05 6.316e-05
## Proportion of Variance 0.000e+00 0.000e+00 0.000e+00 0.000e+00 0.000e+00
## Cumulative Proportion 1.000e+00 1.000e+00 1.000e+00 1.000e+00 1.000e+00
## PC350 PC351 PC352 PC353 PC354
## Standard deviation 6.165e-05 6.107e-05 6.046e-05 6.03e-05 5.894e-05
## Proportion of Variance 0.000e+00 0.000e+00 0.000e+00 0.00e+00 0.000e+00
## Cumulative Proportion 1.000e+00 1.000e+00 1.000e+00 1.00e+00 1.000e+00
## PC355 PC356 PC357 PC358 PC359
## Standard deviation 5.591e-05 5.545e-05 5.451e-05 5.24e-05 5.081e-05
## Proportion of Variance 0.000e+00 0.000e+00 0.000e+00 0.00e+00 0.000e+00
## Cumulative Proportion 1.000e+00 1.000e+00 1.000e+00 1.00e+00 1.000e+00
## PC360 PC361 PC362 PC363 PC364
## Standard deviation 5.032e-05 4.933e-05 4.839e-05 4.711e-05 4.692e-05
## Proportion of Variance 0.000e+00 0.000e+00 0.000e+00 0.000e+00 0.000e+00
## Cumulative Proportion 1.000e+00 1.000e+00 1.000e+00 1.000e+00 1.000e+00
## PC365 PC366 PC367 PC368 PC369
## Standard deviation 4.565e-05 4.502e-05 4.436e-05 4.223e-05 4.146e-05
## Proportion of Variance 0.000e+00 0.000e+00 0.000e+00 0.000e+00 0.000e+00
## Cumulative Proportion 1.000e+00 1.000e+00 1.000e+00 1.000e+00 1.000e+00
## PC370 PC371 PC372 PC373 PC374
## Standard deviation 4.039e-05 3.966e-05 3.841e-05 3.696e-05 3.645e-05
## Proportion of Variance 0.000e+00 0.000e+00 0.000e+00 0.000e+00 0.000e+00
## Cumulative Proportion 1.000e+00 1.000e+00 1.000e+00 1.000e+00 1.000e+00
## PC375 PC376 PC377 PC378 PC379
## Standard deviation 3.505e-05 3.462e-05 3.39e-05 3.356e-05 3.258e-05
## Proportion of Variance 0.000e+00 0.000e+00 0.00e+00 0.000e+00 0.000e+00
## Cumulative Proportion 1.000e+00 1.000e+00 1.00e+00 1.000e+00 1.000e+00
## PC380 PC381 PC382 PC383 PC384
## Standard deviation 3.079e-05 3.038e-05 3.017e-05 2.899e-05 2.873e-05
## Proportion of Variance 0.000e+00 0.000e+00 0.000e+00 0.000e+00 0.000e+00
## Cumulative Proportion 1.000e+00 1.000e+00 1.000e+00 1.000e+00 1.000e+00
## PC385 PC386 PC387 PC388 PC389
## Standard deviation 2.74e-05 2.617e-05 2.598e-05 2.568e-05 2.542e-05
## Proportion of Variance 0.00e+00 0.000e+00 0.000e+00 0.000e+00 0.000e+00
## Cumulative Proportion 1.00e+00 1.000e+00 1.000e+00 1.000e+00 1.000e+00
## PC390 PC391 PC392 PC393 PC394
## Standard deviation 2.395e-05 2.35e-05 2.282e-05 2.187e-05 2.154e-05
## Proportion of Variance 0.000e+00 0.00e+00 0.000e+00 0.000e+00 0.000e+00
## Cumulative Proportion 1.000e+00 1.00e+00 1.000e+00 1.000e+00 1.000e+00
## PC395 PC396 PC397 PC398 PC399
## Standard deviation 2.085e-05 2.059e-05 1.971e-05 1.91e-05 1.874e-05
## Proportion of Variance 0.000e+00 0.000e+00 0.000e+00 0.00e+00 0.000e+00
## Cumulative Proportion 1.000e+00 1.000e+00 1.000e+00 1.00e+00 1.000e+00
## PC400 PC401 PC402 PC403 PC404
## Standard deviation 1.808e-05 1.765e-05 1.703e-05 1.685e-05 1.641e-05
## Proportion of Variance 0.000e+00 0.000e+00 0.000e+00 0.000e+00 0.000e+00
## Cumulative Proportion 1.000e+00 1.000e+00 1.000e+00 1.000e+00 1.000e+00
## PC405 PC406 PC407 PC408 PC409
## Standard deviation 1.624e-05 1.595e-05 1.523e-05 1.455e-05 1.413e-05
## Proportion of Variance 0.000e+00 0.000e+00 0.000e+00 0.000e+00 0.000e+00
## Cumulative Proportion 1.000e+00 1.000e+00 1.000e+00 1.000e+00 1.000e+00
## PC410 PC411 PC412 PC413 PC414
## Standard deviation 1.349e-05 1.318e-05 1.285e-05 1.25e-05 1.211e-05
## Proportion of Variance 0.000e+00 0.000e+00 0.000e+00 0.00e+00 0.000e+00
## Cumulative Proportion 1.000e+00 1.000e+00 1.000e+00 1.00e+00 1.000e+00
## PC415 PC416 PC417 PC418 PC419
## Standard deviation 1.162e-05 1.117e-05 1.075e-05 1.065e-05 1.052e-05
## Proportion of Variance 0.000e+00 0.000e+00 0.000e+00 0.000e+00 0.000e+00
## Cumulative Proportion 1.000e+00 1.000e+00 1.000e+00 1.000e+00 1.000e+00
## PC420 PC421 PC422 PC423 PC424
## Standard deviation 1.038e-05 9.855e-06 9.583e-06 9.229e-06 8.954e-06
## Proportion of Variance 0.000e+00 0.000e+00 0.000e+00 0.000e+00 0.000e+00
## Cumulative Proportion 1.000e+00 1.000e+00 1.000e+00 1.000e+00 1.000e+00
## PC425 PC426 PC427 PC428 PC429
## Standard deviation 8.575e-06 8.201e-06 7.874e-06 7.766e-06 7.147e-06
## Proportion of Variance 0.000e+00 0.000e+00 0.000e+00 0.000e+00 0.000e+00
## Cumulative Proportion 1.000e+00 1.000e+00 1.000e+00 1.000e+00 1.000e+00
## PC430 PC431 PC432 PC433 PC434
## Standard deviation 6.934e-06 6.826e-06 6.519e-06 6.247e-06 6.083e-06
## Proportion of Variance 0.000e+00 0.000e+00 0.000e+00 0.000e+00 0.000e+00
## Cumulative Proportion 1.000e+00 1.000e+00 1.000e+00 1.000e+00 1.000e+00
## PC435 PC436 PC437 PC438 PC439
## Standard deviation 5.913e-06 5.813e-06 5.534e-06 5.159e-06 5.107e-06
## Proportion of Variance 0.000e+00 0.000e+00 0.000e+00 0.000e+00 0.000e+00
## Cumulative Proportion 1.000e+00 1.000e+00 1.000e+00 1.000e+00 1.000e+00
## PC440 PC441 PC442 PC443 PC444
## Standard deviation 4.768e-06 4.636e-06 4.493e-06 4.458e-06 4.225e-06
## Proportion of Variance 0.000e+00 0.000e+00 0.000e+00 0.000e+00 0.000e+00
## Cumulative Proportion 1.000e+00 1.000e+00 1.000e+00 1.000e+00 1.000e+00
## PC445 PC446 PC447 PC448 PC449
## Standard deviation 4.02e-06 3.656e-06 3.378e-06 2.994e-06 2.564e-06
## Proportion of Variance 0.00e+00 0.000e+00 0.000e+00 0.000e+00 0.000e+00
## Cumulative Proportion 1.00e+00 1.000e+00 1.000e+00 1.000e+00 1.000e+00
## PC450 PC451 PC452 PC453 PC454
## Standard deviation 2.264e-06 1.935e-06 1.228e-06 1.114e-06 8.272e-07
## Proportion of Variance 0.000e+00 0.000e+00 0.000e+00 0.000e+00 0.000e+00
## Cumulative Proportion 1.000e+00 1.000e+00 1.000e+00 1.000e+00 1.000e+00
## PC455 PC456 PC457 PC458 PC459
## Standard deviation 7.242e-07 5.323e-07 4.714e-07 4.502e-07 4.29e-07
## Proportion of Variance 0.000e+00 0.000e+00 0.000e+00 0.000e+00 0.00e+00
## Cumulative Proportion 1.000e+00 1.000e+00 1.000e+00 1.000e+00 1.00e+00
## PC460 PC461 PC462 PC463 PC464
## Standard deviation 4.014e-07 3.794e-07 3.69e-07 3.408e-07 3.305e-07
## Proportion of Variance 0.000e+00 0.000e+00 0.00e+00 0.000e+00 0.000e+00
## Cumulative Proportion 1.000e+00 1.000e+00 1.00e+00 1.000e+00 1.000e+00
## PC465 PC466 PC467 PC468 PC469
## Standard deviation 3.228e-07 3.2e-07 3.126e-07 3.031e-07 2.949e-07
## Proportion of Variance 0.000e+00 0.0e+00 0.000e+00 0.000e+00 0.000e+00
## Cumulative Proportion 1.000e+00 1.0e+00 1.000e+00 1.000e+00 1.000e+00
## PC470 PC471 PC472 PC473 PC474
## Standard deviation 2.855e-07 2.811e-07 2.748e-07 2.73e-07 2.703e-07
## Proportion of Variance 0.000e+00 0.000e+00 0.000e+00 0.00e+00 0.000e+00
## Cumulative Proportion 1.000e+00 1.000e+00 1.000e+00 1.00e+00 1.000e+00
## PC475 PC476 PC477 PC478 PC479
## Standard deviation 2.595e-07 2.555e-07 2.497e-07 2.475e-07 2.426e-07
## Proportion of Variance 0.000e+00 0.000e+00 0.000e+00 0.000e+00 0.000e+00
## Cumulative Proportion 1.000e+00 1.000e+00 1.000e+00 1.000e+00 1.000e+00
## PC480 PC481 PC482 PC483 PC484
## Standard deviation 2.414e-07 2.405e-07 2.384e-07 2.359e-07 2.297e-07
## Proportion of Variance 0.000e+00 0.000e+00 0.000e+00 0.000e+00 0.000e+00
## Cumulative Proportion 1.000e+00 1.000e+00 1.000e+00 1.000e+00 1.000e+00
## PC485 PC486 PC487 PC488 PC489
## Standard deviation 2.273e-07 2.262e-07 2.221e-07 2.188e-07 2.168e-07
## Proportion of Variance 0.000e+00 0.000e+00 0.000e+00 0.000e+00 0.000e+00
## Cumulative Proportion 1.000e+00 1.000e+00 1.000e+00 1.000e+00 1.000e+00
## PC490 PC491 PC492 PC493 PC494
## Standard deviation 2.148e-07 2.122e-07 2.102e-07 2.091e-07 2.052e-07
## Proportion of Variance 0.000e+00 0.000e+00 0.000e+00 0.000e+00 0.000e+00
## Cumulative Proportion 1.000e+00 1.000e+00 1.000e+00 1.000e+00 1.000e+00
## PC495 PC496 PC497 PC498 PC499
## Standard deviation 2.038e-07 2.011e-07 1.984e-07 1.972e-07 1.929e-07
## Proportion of Variance 0.000e+00 0.000e+00 0.000e+00 0.000e+00 0.000e+00
## Cumulative Proportion 1.000e+00 1.000e+00 1.000e+00 1.000e+00 1.000e+00
## PC500 PC501 PC502 PC503 PC504
## Standard deviation 1.916e-07 1.897e-07 1.862e-07 1.846e-07 1.828e-07
## Proportion of Variance 0.000e+00 0.000e+00 0.000e+00 0.000e+00 0.000e+00
## Cumulative Proportion 1.000e+00 1.000e+00 1.000e+00 1.000e+00 1.000e+00
## PC505 PC506 PC507 PC508 PC509
## Standard deviation 1.814e-07 1.798e-07 1.762e-07 1.752e-07 1.732e-07
## Proportion of Variance 0.000e+00 0.000e+00 0.000e+00 0.000e+00 0.000e+00
## Cumulative Proportion 1.000e+00 1.000e+00 1.000e+00 1.000e+00 1.000e+00
## PC510 PC511 PC512 PC513 PC514
## Standard deviation 1.703e-07 1.698e-07 1.678e-07 1.656e-07 1.643e-07
## Proportion of Variance 0.000e+00 0.000e+00 0.000e+00 0.000e+00 0.000e+00
## Cumulative Proportion 1.000e+00 1.000e+00 1.000e+00 1.000e+00 1.000e+00
## PC515 PC516 PC517 PC518 PC519
## Standard deviation 1.615e-07 1.582e-07 1.548e-07 1.537e-07 1.518e-07
## Proportion of Variance 0.000e+00 0.000e+00 0.000e+00 0.000e+00 0.000e+00
## Cumulative Proportion 1.000e+00 1.000e+00 1.000e+00 1.000e+00 1.000e+00

pca_scoresplot2D(ir.management.ds,ir.corte.pca, "Profundidade",labels=F,pallette=2, ellipses = T)


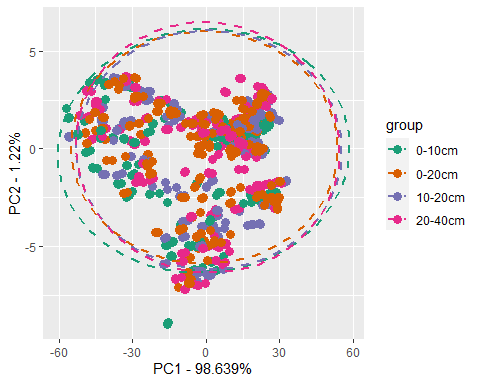


pca_scoresplot2D(ir.management.ds,ir.corte.pca, "Manejo",labels=F,pallette=2, ellipses = T)


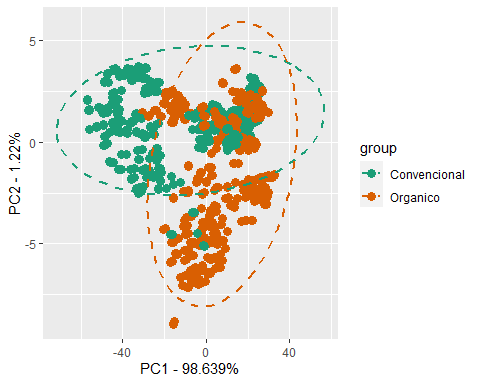


pca_scoresplot2D(ir.management.ds,ir.corte.pca, "Especie",labels=F,pallette=2, ellipses = T)


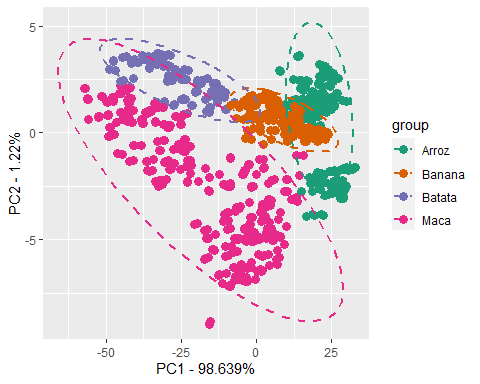


**Hierarchical Cluster Analysis** ir.hc = clustering(ir.corte, method = “hc”, distance = “euclidean”) dendrogram_plot(ir.corte, ir.hc, “Profundidade”) dendrogram_plot_col(ir.corte, ir.hc, “Profundidade”)

```r
ir.hc = clustering(ir.corte, method = "hc", distance = "euclidean")
dendrogram_plot(ir.corte, ir.hc, "Manejo")


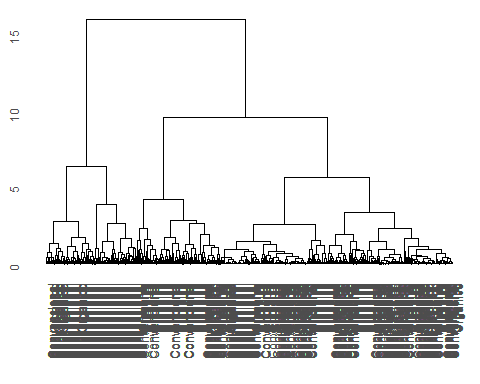


dendrogram_plot_col(ir.corte, ir.hc, "Manejo")


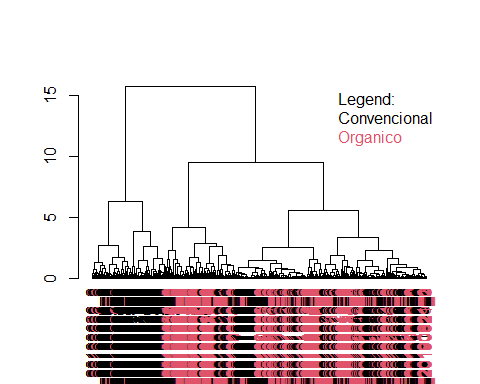


ir.hc = clustering(ir.corte, method = "hc", distance = "euclidean")
dendrogram_plot(ir.corte, ir.hc, "Especie")


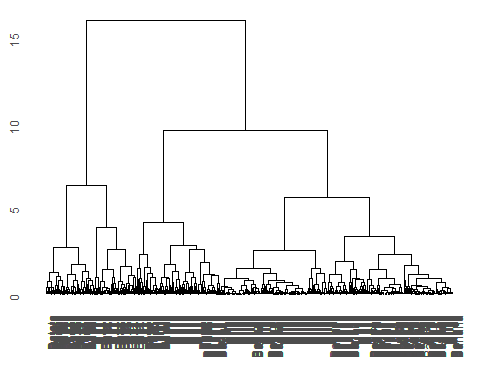


dendrogram_plot_col(ir.corte, ir.hc, "Especie")


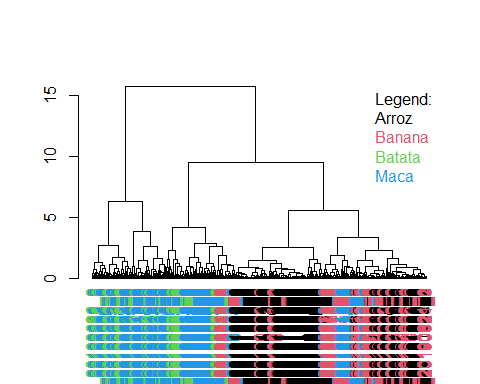


##UNIVARIATE ANALYSIS##

ir.anova = aov_all_vars(ir.corte,"Profundidade")
ir.anova[1:10,]

## pvalues logs fdr tukey
## 7999.7287028621 0.3964775 0.4017815 0.6588703
## 7992.01440338248 0.3971833 0.4010090 0.6588703
## 7984.30010390286 0.3983838 0.3996983 0.6588703
## 7976.58580442324 0.4001056 0.3978254 0.6588703
## 7968.87150494363 0.4015816 0.3962261 0.6588703
## 7961.15720546401 0.4024601 0.3952771 0.6588703
## 7953.44290598439 0.4033849 0.3942804 0.6588703
## 7945.72860650477 0.4048135 0.3927450 0.6588703
## 7938.01430702515 0.4066640 0.3907643 0.6588703
## 7930.30000754553 0.4083940 0.3889207 0.6588703

ir.anova = aov_all_vars(ir.corte,"Manejo")
ir.anova[1:10,]

## pvalues logs fdr tukey
## 5947.72504128361 1.069980e-44 43.97062 2.584291e-43 Organico-Convencional
## 5955.43934076323 1.079041e-44 43.96696 2.584291e-43 Organico-Convencional
## 5940.01074180399 1.082447e-44 43.96559 2.584291e-43 Organico-Convencional
## 5963.15364024284 1.100388e-44 43.95845 2.584291e-43 Organico-Convencional
## 5932.29644232437 1.105821e-44 43.95632 2.584291e-43 Organico-Convencional
## 5970.86793972246 1.119556e-44 43.95095 2.584291e-43 Organico-Convencional
## 5924.58214284475 1.120817e-44 43.95047 2.584291e-43 Organico-Convencional
## 5978.58223920208 1.131758e-44 43.94625 2.584291e-43 Organico-Convencional
## 5916.86784336513 1.132047e-44 43.94614 2.584291e-43 Organico-Convencional
## 5986.2965386817 1.146076e-44 43.94079 2.584291e-43 Organico-Convencional

ir.anova = aov_all_vars(ir.corte,"Especie")
ir.anova[1:10,]

## pvalues logs fdr
## 7089.44136426713 0 Inf 0
## 7081.72706478751 0 Inf 0
## 7074.01276530789 0 Inf 0
## 7066.29846582827 0 Inf 0
## 7058.58416634866 0 Inf 0
## 7050.86986686904 0 Inf 0
## 5307.43818447528 0 Inf 0
## 5299.72388499566 0 Inf 0
## 5292.00958551604 0 Inf 0
## 5284.29528603643 0 Inf 0
## tukey
## 7089.44136426713 Banana-Arroz; Batata-Arroz; Maca-Arroz; Batata-Banana; Maca-Banana; Maca-Batata
## 7081.72706478751 Banana-Arroz; Batata-Arroz; Maca-Arroz; Batata-Banana; Maca-Banana; Maca-Batata
## 7074.01276530789 Banana-Arroz; Batata-Arroz; Maca-Arroz; Batata-Banana; Maca-Banana; Maca-Batata
## 7066.29846582827 Banana-Arroz; Batata-Arroz; Maca-Arroz; Batata-Banana; Maca-Banana; Maca-Batata
## 7058.58416634866 Banana-Arroz; Batata-Arroz; Maca-Arroz; Batata-Banana; Maca-Banana; Maca-Batata
## 7050.86986686904 Banana-Arroz; Batata-Arroz; Maca-Arroz; Batata-Banana; Maca-Banana; Maca-Batata
## 5307.43818447528 Banana-Arroz; Batata-Arroz; Maca-Arroz; Batata-Banana; Maca-Banana; Maca-Batata
## 5299.72388499566 Banana-Arroz; Batata-Arroz; Maca-Arroz; Batata-Banana; Maca-Banana; Maca-Batata
## 5292.00958551604 Banana-Arroz; Batata-Arroz; Maca-Arroz; Batata-Banana; Maca-Banana; Maca-Batata
## 5284.29528603643 Banana-Arroz; Batata-Arroz; Maca-Arroz; Batata-Banana; Maca-Banana

#T test

ir.convencional.organico = subset_samples_by_metadata_values(ir.corte, "Manejo", values = c("Convencional","Organico"))
ir.ttest = tTests_dataset(ir.convencional.organico, "Manejo")
ir.ttest[1:10,]

## p.value -log10 fdr
## 5947.72504128361 1.069980e-44 43.97062 2.584291e-43
## 5955.43934076323 1.079041e-44 43.96696 2.584291e-43
## 5940.01074180399 1.082447e-44 43.96559 2.584291e-43
## 5963.15364024284 1.100388e-44 43.95845 2.584291e-43
## 5932.29644232437 1.105821e-44 43.95632 2.584291e-43
## 5970.86793972246 1.119556e-44 43.95095 2.584291e-43
## 5924.58214284475 1.120817e-44 43.95047 2.584291e-43
## 5978.58223920208 1.131758e-44 43.94625 2.584291e-43
## 5916.86784336513 1.132047e-44 43.94614 2.584291e-43
## 5986.2965386817 1.146076e-44 43.94079 2.584291e-43

plot_ttests(ir.convencional.organico, ir.ttest, tt.threshold = 0.05)


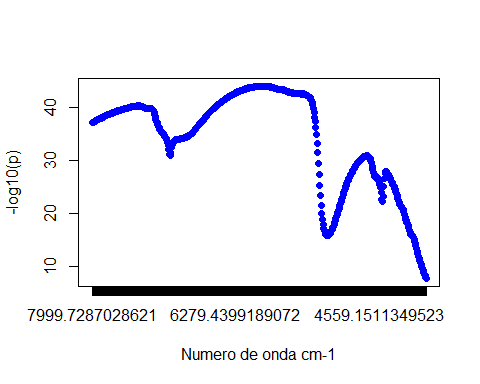


ir.convencional.organico = subset_samples_by_metadata_values(ir.corte, "Manejo", values = c("Convencional","Organico"))

ir.banana.batata = subset_samples_by_metadata_values(ir.corte, "Especie", values = c("Banana","Batata"))
ir.ttest = tTests_dataset(ir.banana.batata, "Especie")
ir.ttest[1:10,]

## p.value -log10 fdr
## 7999.7287028621 1.334579e-141 140.8747 2.579217e-139
## 7992.01440338248 1.738644e-141 140.7598 2.579217e-139
## 7984.30010390286 2.099737e-141 140.6778 2.579217e-139
## 7976.58580442324 2.345859e-141 140.6297 2.579217e-139
## 7968.87150494363 2.639236e-141 140.5785 2.579217e-139
## 7961.15720546401 3.134308e-141 140.5039 2.579217e-139
## 7953.44290598439 3.636608e-141 140.4393 2.579217e-139
## 7945.72860650477 3.975671e-141 140.4006 2.579217e-139
## 7938.01430702515 4.513545e-141 140.3455 2.602811e-139
## 7930.30000754553 5.640857e-141 140.2487 2.927605e-139

plot_ttests(ir.banana.batata, ir.ttest, tt.threshold = 0.05)


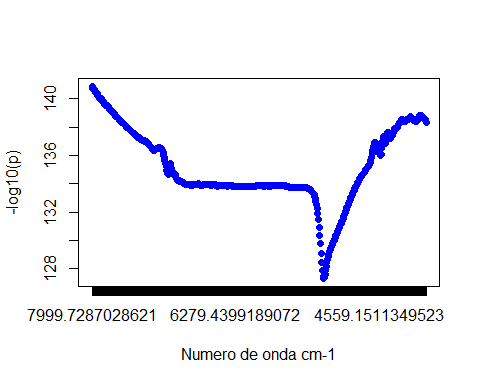


ir.banana.batata = subset_samples_by_metadata_values(ir.corte, "Especie", values = c("Banana","Batata"))

ir.maca.batata = subset_samples_by_metadata_values(ir.corte, "Especie", values = c("Maca","Batata"))
ir.ttest = tTests_dataset(ir.maca.batata, "Especie")
ir.ttest[1:10,]

## p.value -log10 fdr
## 4003.72157241977 4.232241e-10 9.373430 9.043211e-08
## 4011.43587189939 1.460776e-09 8.835417 9.043211e-08
## 4019.15017137901 5.503051e-09 8.259397 9.043211e-08
## 7590.87083044232 1.179241e-08 7.928397 9.043211e-08
## 7598.58512992194 1.186134e-08 7.925866 9.043211e-08
## 7583.15653096271 1.194589e-08 7.922781 9.043211e-08
## 7560.01363252385 1.199125e-08 7.921135 9.043211e-08
## 7552.29933304423 1.202527e-08 7.919905 9.043211e-08
## 7567.72793200347 1.204586e-08 7.919162 9.043211e-08
## 7575.44223148309 1.207965e-08 7.917946 9.043211e-08

plot_ttests(ir.banana.batata, ir.ttest, tt.threshold = 0.05)


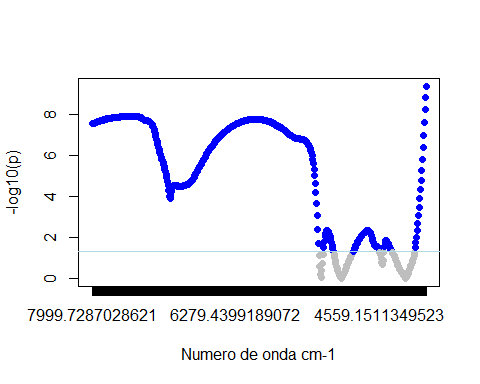


ir.maca.batata = subset_samples_by_metadata_values(ir.corte, "Especie", values = c("Maca","Batata"))

ir.banana.arroz = subset_samples_by_metadata_values(ir.corte, "Especie", values = c("Banana","Batata"))
ir.ttest = tTests_dataset(ir.banana.arroz, "Especie")
ir.ttest[1:10,]

## p.value -log10 fdr
## 7999.7287028621 1.334579e-141 140.8747 2.579217e-139
## 7992.01440338248 1.738644e-141 140.7598 2.579217e-139
## 7984.30010390286 2.099737e-141 140.6778 2.579217e-139
## 7976.58580442324 2.345859e-141 140.6297 2.579217e-139
## 7968.87150494363 2.639236e-141 140.5785 2.579217e-139
## 7961.15720546401 3.134308e-141 140.5039 2.579217e-139
## 7953.44290598439 3.636608e-141 140.4393 2.579217e-139
## 7945.72860650477 3.975671e-141 140.4006 2.579217e-139
## 7938.01430702515 4.513545e-141 140.3455 2.602811e-139
## 7930.30000754553 5.640857e-141 140.2487 2.927605e-139

plot_ttests(ir.banana.arroz, ir.ttest, tt.threshold = 0.05)


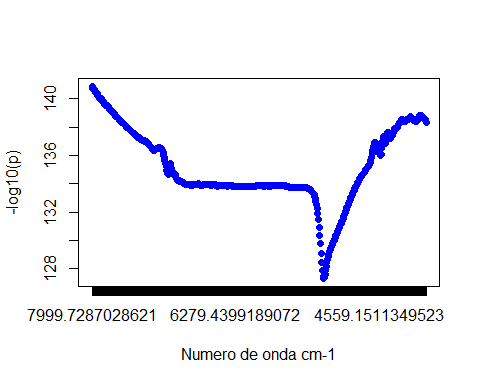


ir.banana.arroz = subset_samples_by_metadata_values(ir.corte, "Especie", values = c("Banana","Batata"))

#FoldChange Analysis with normalized data**

ir.fc = fold_change(ir.convencional.organico, "Manejo", ref.value = "Convencional")
plot_fold_change(ir.corte, ir.fc, fc.threshold = 2)


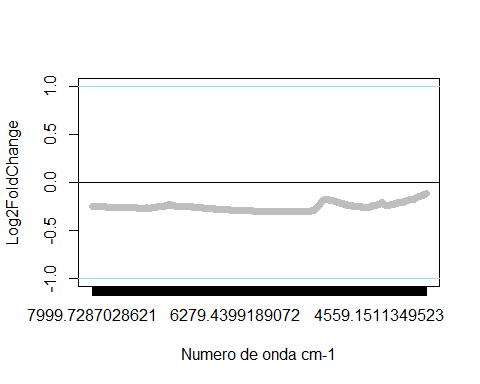


ir.fc = fold_change(ir.convencional.organico, "Manejo", ref.value = "Organico")
plot_fold_change(ir.corte, ir.fc, fc.threshold = 2)


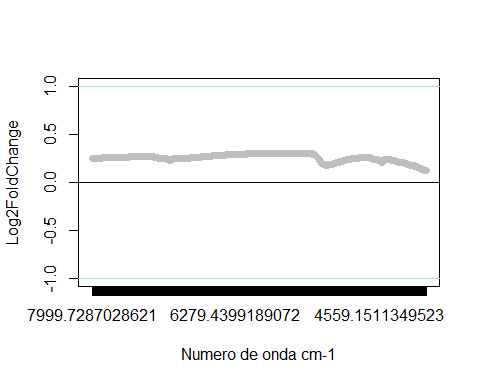


**Management FINGERPRINT REGION (4700.0 3500.0 cm-1)** Importance of components: Proportion of Variance explained in each component

ir.cortee = subset_x_values_by_interval(ir.management.ds, min.value = 3500, max.value = 4700)

**Plotting the Spectra**

plot_spectra_simple(ir.cortee)


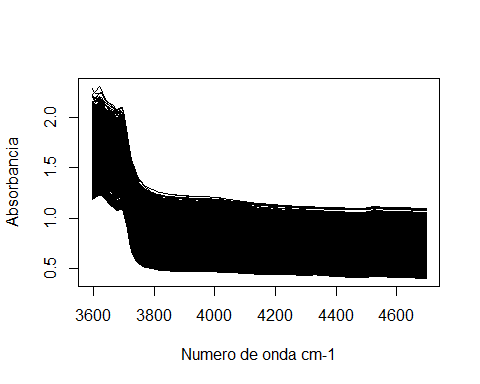


plot_spectra(ir.cortee, "Profundidade", legend.place = "topright", cex = 0.76)


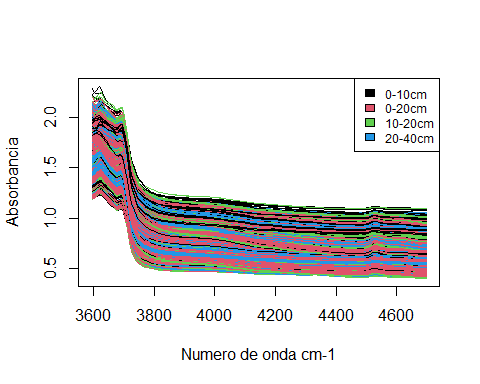


plot_spectra(ir.cortee, "Manejo", legend.place = "topright", cex = 0.76)


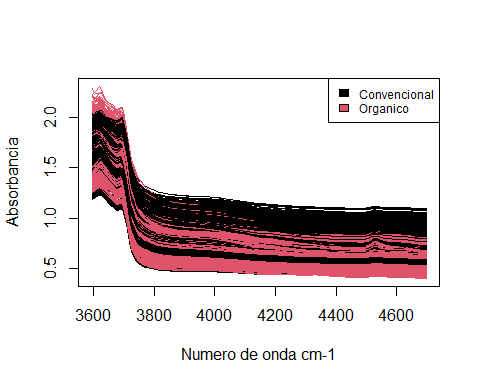


plot_spectra(ir.cortee, "Especie", legend.place = "topright", cex = 0.76)


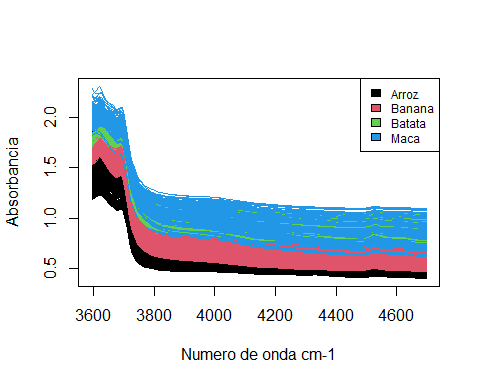


**Principal Components Analysis**

ir.cortee.pca = pca_analysis_dataset(ir.cortee)
summary(ir.cortee.pca)

## Importance of components:
## PC1 PC2 PC3 PC4 PC5 PC6 PC7
## Standard deviation 11.7184 2.45705 0.70233 0.32655 0.10892 0.09443 0.08233
## Proportion of Variance 0.9536 0.04192 0.00343 0.00074 0.00008 0.00006 0.00005
## Cumulative Proportion 0.9536 0.99554 0.99896 0.99971 0.99979 0.99985 0.99990
## PC8 PC9 PC10 PC11 PC12 PC13 PC14
## Standard deviation 0.07098 0.05204 0.04558 0.03542 0.03423 0.02568 0.02253
## Proportion of Variance 0.00003 0.00002 0.00001 0.00001 0.00001 0.00000 0.00000
## Cumulative Proportion 0.99993 0.99995 0.99997 0.99997 0.99998 0.99999 0.99999
## PC15 PC16 PC17 PC18 PC19 PC20
## Standard deviation 0.01884 0.01665 0.01441 0.01286 0.009631 0.009494
## Proportion of Variance 0.00000 0.00000 0.00000 0.00000 0.000000 0.000000
## Cumulative Proportion 0.99999 0.99999 1.00000 1.00000 1.000000 1.000000
## PC21 PC22 PC23 PC24 PC25 PC26
## Standard deviation 0.007033 0.006282 0.005492 0.00469 0.004156 0.003983
## Proportion of Variance 0.000000 0.000000 0.000000 0.00000 0.000000 0.000000
## Cumulative Proportion 1.000000 1.000000 1.000000 1.00000 1.000000 1.000000
## PC27 PC28 PC29 PC30 PC31 PC32
## Standard deviation 0.003235 0.002797 0.002668 0.002301 0.002102 0.002035
## Proportion of Variance 0.000000 0.000000 0.000000 0.000000 0.000000 0.000000
## Cumulative Proportion 1.000000 1.000000 1.000000 1.000000 1.000000 1.000000
## PC33 PC34 PC35 PC36 PC37 PC38
## Standard deviation 0.001702 0.001436 0.001362 0.001273 0.001187 0.001127
## Proportion of Variance 0.000000 0.000000 0.000000 0.000000 0.000000 0.000000
## Cumulative Proportion 1.000000 1.000000 1.000000 1.000000 1.000000 1.000000
## PC39 PC40 PC41 PC42 PC43 PC44
## Standard deviation 0.00111 0.001061 0.001049 0.001013 0.000968 0.0009562
## Proportion of Variance 0.00000 0.000000 0.000000 0.000000 0.000000 0.0000000
## Cumulative Proportion 1.00000 1.000000 1.000000 1.000000 1.000000 1.0000000
## PC45 PC46 PC47 PC48 PC49
## Standard deviation 0.0009176 0.0008587 0.0008477 0.0008232 0.000797
## Proportion of Variance 0.0000000 0.0000000 0.0000000 0.0000000 0.000000
## Cumulative Proportion 1.0000000 1.0000000 1.0000000 1.0000000 1.000000
## PC50 PC51 PC52 PC53 PC54
## Standard deviation 0.0007758 0.0007483 0.000738 0.0007031 0.0006905
## Proportion of Variance 0.0000000 0.0000000 0.000000 0.0000000 0.0000000
## Cumulative Proportion 1.0000000 1.0000000 1.000000 1.0000000 1.0000000
## PC55 PC56 PC57 PC58 PC59
## Standard deviation 0.0006757 0.000638 0.0006283 0.0006027 0.0005776
## Proportion of Variance 0.0000000 0.000000 0.0000000 0.0000000 0.0000000
## Cumulative Proportion 1.0000000 1.000000 1.0000000 1.0000000 1.0000000
## PC60 PC61 PC62 PC63 PC64
## Standard deviation 0.0005675 0.0005438 0.0005258 0.0005176 0.0004935
## Proportion of Variance 0.0000000 0.0000000 0.0000000 0.0000000 0.0000000
## Cumulative Proportion 1.0000000 1.0000000 1.0000000 1.0000000 1.0000000
## PC65 PC66 PC67 PC68 PC69
## Standard deviation 0.0004836 0.0004716 0.0004363 0.0004283 0.0004077
## Proportion of Variance 0.0000000 0.0000000 0.0000000 0.0000000 0.0000000
## Cumulative Proportion 1.0000000 1.0000000 1.0000000 1.0000000 1.0000000
## PC70 PC71 PC72 PC73 PC74
## Standard deviation 0.0003981 0.0003746 0.0003595 0.000347 0.000335
## Proportion of Variance 0.0000000 0.0000000 0.0000000 0.000000 0.000000
## Cumulative Proportion 1.0000000 1.0000000 1.0000000 1.000000 1.000000
## PC75 PC76 PC77 PC78 PC79
## Standard deviation 0.0003298 0.0003042 0.000299 0.0002821 0.0002691
## Proportion of Variance 0.0000000 0.0000000 0.000000 0.0000000 0.0000000
## Cumulative Proportion 1.0000000 1.0000000 1.000000 1.0000000 1.0000000
## PC80 PC81 PC82 PC83 PC84
## Standard deviation 0.000259 0.0002464 0.0002247 0.0002196 0.0002081
## Proportion of Variance 0.000000 0.0000000 0.0000000 0.0000000 0.0000000
## Cumulative Proportion 1.000000 1.0000000 1.0000000 1.0000000 1.0000000
## PC85 PC86 PC87 PC88 PC89
## Standard deviation 0.0001938 0.0001851 0.0001803 0.0001685 0.0001629
## Proportion of Variance 0.0000000 0.0000000 0.0000000 0.0000000 0.0000000
## Cumulative Proportion 1.0000000 1.0000000 1.0000000 1.0000000 1.0000000
## PC90 PC91 PC92 PC93 PC94
## Standard deviation 0.0001496 0.000136 0.0001323 0.0001262 0.0001169
## Proportion of Variance 0.0000000 0.000000 0.0000000 0.0000000 0.0000000
## Cumulative Proportion 1.0000000 1.000000 1.0000000 1.0000000 1.0000000
## PC95 PC96 PC97 PC98 PC99 PC100
## Standard deviation 0.0001137 0.000104 9.68e-05 9.142e-05 8.535e-05 7.83e-05
## Proportion of Variance 0.0000000 0.000000 0.00e+00 0.000e+00 0.000e+00 0.00e+00
## Cumulative Proportion 1.0000000 1.000000 1.00e+00 1.000e+00 1.000e+00 1.00e+00
## PC101 PC102 PC103 PC104 PC105
## Standard deviation 6.995e-05 6.844e-05 6.18e-05 5.779e-05 5.274e-05
## Proportion of Variance 0.000e+00 0.000e+00 0.00e+00 0.000e+00 0.000e+00
## Cumulative Proportion 1.000e+00 1.000e+00 1.00e+00 1.000e+00 1.000e+00
## PC106 PC107 PC108 PC109 PC110
## Standard deviation 4.852e-05 4.552e-05 4.038e-05 3.624e-05 3.412e-05
## Proportion of Variance 0.000e+00 0.000e+00 0.000e+00 0.000e+00 0.000e+00
## Cumulative Proportion 1.000e+00 1.000e+00 1.000e+00 1.000e+00 1.000e+00
## PC111 PC112 PC113 PC114 PC115
## Standard deviation 3.025e-05 2.673e-05 2.542e-05 2.315e-05 2.061e-05
## Proportion of Variance 0.000e+00 0.000e+00 0.000e+00 0.000e+00 0.000e+00
## Cumulative Proportion 1.000e+00 1.000e+00 1.000e+00 1.000e+00 1.000e+00
## PC116 PC117 PC118 PC119 PC120
## Standard deviation 1.811e-05 1.62e-05 1.516e-05 1.286e-05 1.14e-05
## Proportion of Variance 0.000e+00 0.00e+00 0.000e+00 0.000e+00 0.00e+00
## Cumulative Proportion 1.000e+00 1.00e+00 1.000e+00 1.000e+00 1.00e+00
## PC121 PC122 PC123 PC124 PC125
## Standard deviation 1.022e-05 8.766e-06 8.111e-06 6.873e-06 6.381e-06
## Proportion of Variance 0.000e+00 0.000e+00 0.000e+00 0.000e+00 0.000e+00
## Cumulative Proportion 1.000e+00 1.000e+00 1.000e+00 1.000e+00 1.000e+00
## PC126 PC127 PC128 PC129 PC130
## Standard deviation 5.387e-06 4.581e-06 3.208e-06 2.114e-06 1.371e-06
## Proportion of Variance 0.000e+00 0.000e+00 0.000e+00 0.000e+00 0.000e+00
## Cumulative Proportion 1.000e+00 1.000e+00 1.000e+00 1.000e+00 1.000e+00
## PC131 PC132 PC133 PC134 PC135
## Standard deviation 1.072e-06 7.908e-07 6.956e-07 5.237e-07 3.516e-07
## Proportion of Variance 0.000e+00 0.000e+00 0.000e+00 0.000e+00 0.000e+00
## Cumulative Proportion 1.000e+00 1.000e+00 1.000e+00 1.000e+00 1.000e+00
## PC136 PC137 PC138 PC139 PC140
## Standard deviation 3.026e-07 2.596e-07 2.488e-07 2.448e-07 2.382e-07
## Proportion of Variance 0.000e+00 0.000e+00 0.000e+00 0.000e+00 0.000e+00
## Cumulative Proportion 1.000e+00 1.000e+00 1.000e+00 1.000e+00 1.000e+00
## PC141 PC142 PC143 PC144
## Standard deviation 2.31e-07 2.302e-07 2.211e-07 2.155e-07
## Proportion of Variance 0.00e+00 0.000e+00 0.000e+00 0.000e+00
## Cumulative Proportion 1.00e+00 1.000e+00 1.000e+00 1.000e+00

pca_scoresplot2D(ir.management.ds,ir.cortee.pca, "Profundidade",labels=F,pallette=2, ellipses = T)


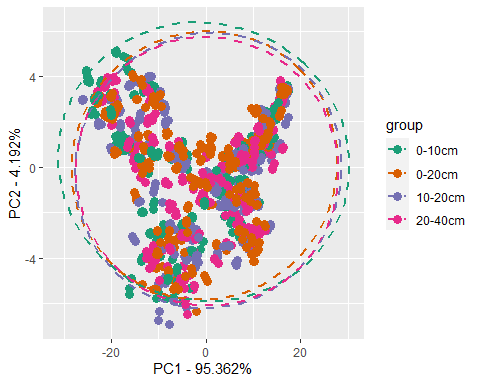


pca_scoresplot2D(ir.management.ds,ir.cortee.pca, "Manejo",labels=F,pallette=2, ellipses = T)


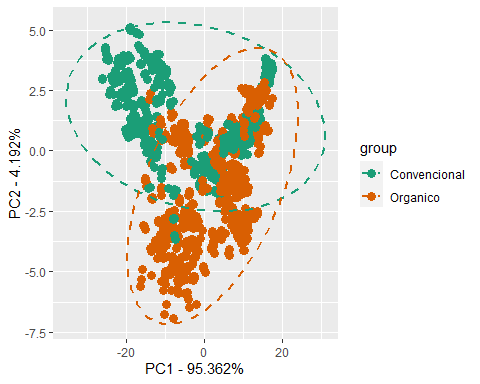


pca_scoresplot2D(ir.management.ds,ir.cortee.pca, "Especie",labels=F,pallette=2, ellipses = T)


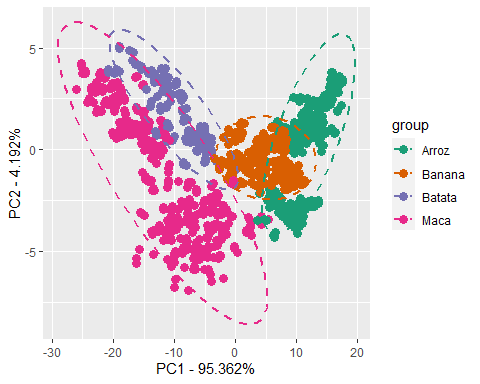


**Hierarchical Cluster Analysis** ir.hc = clustering(ir.cortee, method = “hc”, distance = “euclidean”) dendrogram_plot(ir.cortee, ir.hc, “Profundidade”) dendrogram_plot_col(ir.cortee, ir.hc, “Profundidade”)

```r
ir.hc = clustering(ir.cortee, method = "hc", distance = "euclidean")
dendrogram_plot(ir.cortee, ir.hc, "Manejo")


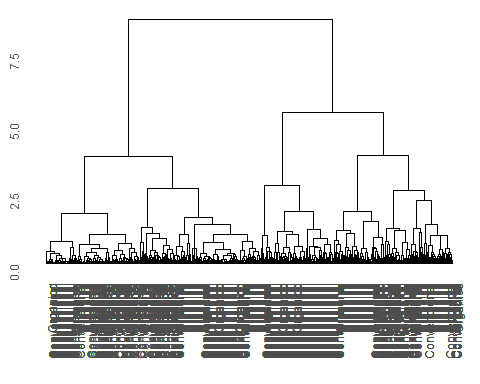


dendrogram_plot_col(ir.cortee, ir.hc, "Manejo")


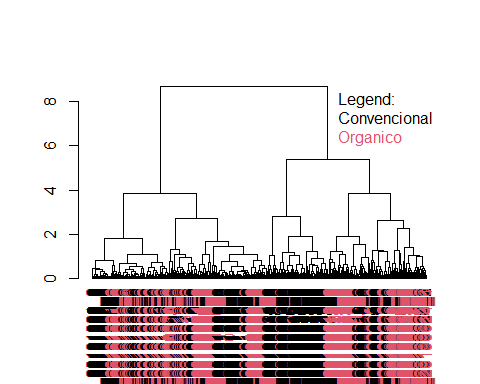


ir.hc = clustering(ir.cortee, method = "hc", distance = "euclidean")
dendrogram_plot(ir.corte, ir.hc, "Especie")


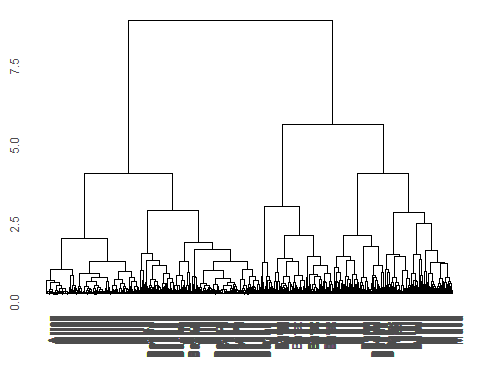


dendrogram_plot_col(ir.cortee, ir.hc, "Especie")


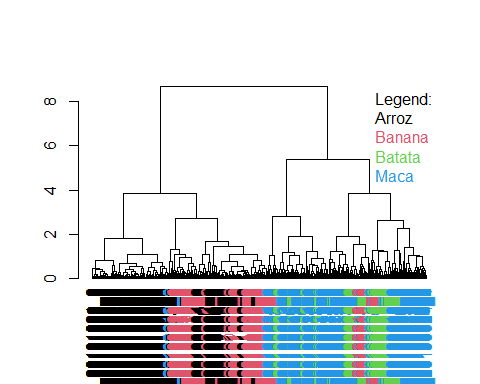


##UNIVARIATE ANALYSIS##

ir.anova = aov_all_vars(ir.cortee,"Profundidade")
ir.anova[1:10,]

## pvalues logs fdr tukey
## 4050.00736929748 0.4946856 0.3056708 0.8667824
## 4042.29306981787 0.4953821 0.3050597 0.8667824
## 4057.7216687771 0.4963545 0.3042080 0.8667824
## 4034.57877033825 0.4975798 0.3031373 0.8667824
## 4065.43596825672 0.4999649 0.3010605 0.8667824
## 4026.86447085863 0.5000663 0.3009724 0.8667824
## 4019.15017137901 0.5021334 0.2991809 0.8667824
## 4011.43587189939 0.5039775 0.2975888 0.8667824
## 4350.8650490026 0.5041200 0.2974661 0.8667824
## 4358.57934848222 0.5042443 0.2973590 0.8667824

ir.anova = aov_all_vars(ir.cortee,"Manejo")
ir.anova[1:10,]

## pvalues logs fdr tukey
## 4698.00852558543 1.533243e-31 30.81439 1.224416e-29 Organico-Convencional
## 4690.29422610581 1.820087e-31 30.73991 1.224416e-29 Organico-Convencional
## 4682.57992662619 2.550866e-31 30.59331 1.224416e-29 Organico-Convencional
## 4674.86562714657 4.426628e-31 30.35393 1.593586e-29 Organico-Convencional
## 4667.15132766696 1.005625e-30 29.99756 2.896199e-29 Organico-Convencional
## 4659.43702818734 3.130523e-30 29.50438 7.513256e-29 Organico-Convencional
## 4651.72272870772 1.265894e-29 28.89760 2.604125e-28 Organico-Convencional
## 4644.0084292281 5.526597e-29 28.25754 9.947874e-28 Organico-Convencional
## 4482.00814015611 1.183303e-28 27.92690 1.865717e-27 Organico-Convencional
## 4489.72243963573 1.295637e-28 27.88752 1.865717e-27 Organico-Convencional

ir.anova = aov_all_vars(ir.cortee,"Especie")
ir.anova[1:10,]

## pvalues logs fdr
## 4698.00852558543 0 Inf 0
## 4690.29422610581 0 Inf 0
## 4682.57992662619 0 Inf 0
## 4674.86562714657 0 Inf 0
## 4667.15132766696 0 Inf 0
## 4659.43702818734 0 Inf 0
## 4651.72272870772 0 Inf 0
## 4644.0084292281 0 Inf 0
## 4636.29412974848 0 Inf 0
## 4628.57983026886 0 Inf 0
## tukey
## 4698.00852558543 Banana-Arroz; Batata-Arroz; Maca-Arroz; Batata-Banana; Maca-Banana; Maca-Batata
## 4690.29422610581 Banana-Arroz; Batata-Arroz; Maca-Arroz; Batata-Banana; Maca-Banana; Maca-Batata
## 4682.57992662619 Banana-Arroz; Batata-Arroz; Maca-Arroz; Batata-Banana; Maca-Banana; Maca-Batata
## 4674.86562714657 Banana-Arroz; Batata-Arroz; Maca-Arroz; Batata-Banana; Maca-Banana; Maca-Batata
## 4667.15132766696 Banana-Arroz; Batata-Arroz; Maca-Arroz; Batata-Banana; Maca-Banana; Maca-Batata
## 4659.43702818734 Banana-Arroz; Batata-Arroz; Maca-Arroz; Batata-Banana; Maca-Banana; Maca-Batata
## 4651.72272870772 Banana-Arroz; Batata-Arroz; Maca-Arroz; Batata-Banana; Maca-Banana; Maca-Batata
## 4644.0084292281 Banana-Arroz; Batata-Arroz; Maca-Arroz; Batata-Banana; Maca-Banana; Maca-Batata
## 4636.29412974848 Banana-Arroz; Batata-Arroz; Maca-Arroz; Batata-Banana; Maca-Banana; Maca-Batata
## 4628.57983026886 Banana-Arroz; Batata-Arroz; Maca-Arroz; Batata-Banana; Maca-Banana; Maca-Batata

**Machine Learning**

```{r}

#set.seed(543217)

install.packages('pls')

install.packages('randomForest')

install.packages('ggplot2')

library("pls")

library("randomForest")

library("ggplot2")

ir.management.ml.soil = train_models_performance(ir.management.ds, c("pls","rf","knn"), "Manejo", "repeatedcv")

ir.management.ml.soil$performance

ir.management.ml.soil$confusion.matrices

ir.management.ml.soil$vips$pls[1:10,]

ir.management.ml.soil$vips$rf[1:10,]

sink()

```

> ir.management.ml.soil$performance

Accuracy Kappa AccuracySD KappaSD

pls 0.8785982 0.7713518 0.01877524 0.03533675

rf 0.9762622 0.9566950 0.01490779 0.02732670

knn 0.9593465 0.9262049 0.01951122 0.03536628

> ir.management.ml.soil$confusion.matrices

$pls

Cross-Validated (10 fold, repeated 10 times) Confusion Matrix

(entries are percentual average cell counts across resamples)

Reference

Prediction AduboEsterco Convencional Organico

AduboEsterco 0.4 0.0 0.0

Convencional 4.7 45.4 6.7

Organico 0.3 0.4 42.1

Accuracy (average): 0.8786

$rf

Cross-Validated (10 fold, repeated 10 times) Confusion Matrix

(entries are percentual average cell counts across resamples)

Reference

Prediction AduboEsterco Convencional Organico

AduboEsterco 5.0 0.2 0.0

Convencional 0.4 44.7 0.8

Organico 0.0 0.9 47.9

Accuracy (average): 0.9763

$knn

Cross-Validated (10 fold, repeated 10 times) Confusion Matrix

(entries are percentual average cell counts across resamples)

Reference

Prediction AduboEsterco Convencional Organico

AduboEsterco 4.5 1.0 0.2

Convencional 0.9 43.9 1.0

Organico 0.0 0.9 47.6

Accuracy (average) : 0.9593

> ir.management.ml.soil$vips$pls[1:10,]

AduboEsterco Convencional Organico Mean

3710.57819219428 28.84542 100.00000 37.48540 55.44361

4543.72253599306 27.52790 86.08740 28.92389 47.51306

4536.00823651344 26.59954 83.41963 27.96643 45.99520

4551.43683547268 25.22219 78.86830 26.63387 43.57479

3718.29249167389 21.96570 78.43424 30.14275 43.51423

4559.1511349523 22.26605 69.79740 23.77712 38.61352

4528.29393703382 21.82688 68.75719 23.08109 37.88838

3702.86389271466 18.50314 66.70569 25.88086 37.02990

4566.86543443192 20.00744 62.92499 21.64725 34.85989

4574.57973391153 18.35984 57.92461 20.10968 32.13138

C:\Users\lindi\AppData\Local\Temp\Rtmp6TcaH6\downloaded_packages

Accuracy Kappa AccuracySD KappaSD

pls 0.8787951 0.7717875 0.02318267 0.04339979

rf 0.9774597 0.9589017 0.01319306 0.02405435

knn 0.9606273 0.9285615 0.01491324 0.02693506

$pls

Cross-Validated (10 fold, repeated 10 times) Confusion Matrix

(entries are percentual average cell counts across resamples)

Reference

Prediction AduboEsterco Convencional Organico

AduboEsterco 0.4 0.0 0.0

Convencional 4.7 45.4 6.7

Organico 0.3 0.4 42.1

Accuracy (average): 0.8788

$rf

Cross-Validated (10 fold, repeated 10 times) Confusion Matrix

(entries are percentual average cell counts across resamples)

Reference

Prediction AduboEsterco Convencional Organico

AduboEsterco 5.1 0.2 0.0

Convencional 0.4 44.6 0.7

Organico 0.0 0.9 48.0

Accuracy (average): 0.9775

$knn

Cross-Validated (10 fold, repeated 10 times) Confusion Matrix

(entries are percentual average cell counts across resamples)

Reference

Prediction AduboEsterco Convencional Organico

AduboEsterco 4.6 0.9 0.3

Convencional 0.9 44.1 1.0

Organico 0.0 0.8 47.4

Accuracy (average): 0.9606

AduboEsterco Convencional Organico Mean

3710.57819219428 28.84542 100.00000 37.48540 55.44361

4543.72253599306 27.52790 86.08740 28.92389 47.51306

4536.00823651344 26.59954 83.41963 27.96643 45.99520

4551.43683547268 25.22219 78.86830 26.63387 43.57479

3718.29249167389 21.96570 78.43424 30.14275 43.51423

4559.1511349523 22.26605 69.79740 23.77712 38.61352

4528.29393703382 21.82688 68.75719 23.08109 37.88838

3702.86389271466 18.50314 66.70569 25.88086 37.02990

4566.86543443192 20.00744 62.92499 21.64725 34.85989

4574.57973391153 18.35984 57.92461 20.10968 32.13138

Overall Mean

3672.00669479618 100.00000 100.00000

3687.43529375542 93.49601 93.49601

3679.7209942758 81.43897 81.43897

3664.29239531656 75.67600 75.67600

3656.57809583695 75.55303 75.55303

3648.86379635733 72.68930 72.68930

3702.86389271466 62.07210 62.07210

3633.43519739809 58.54310 58.54310

3695.14959323504 58.34804 58.34804

3641.14949687771 56.59214 56.59214
